# Global air quality and human health impacts of growing aircraft emissions

**DOI:** 10.1038/s43247-026-03732-4

**Published:** 2026-06-16

**Authors:** Flávio D. A. Quadros, Rick Nelen, Mirjam Snellen, Irene C. Dedoussi

**Affiliations:** 1 Operations and Environment section, Faculty of Aerospace Engineering, Delft University of Technology, Delft, the Netherlands; 2 Whittle Laboratory, Department of Engineering, University of Cambridge, Cambridge, UK

**Keywords:** Environmental impact, Atmospheric chemistry

## Abstract

Aviation is a growing source of atmospheric emissions that degrade air quality both near and far from airports, increasing human mortality. These impacts can vary significantly over time as emissions from aviation and other sectors evolve. Here, we project global aircraft emissions from the year 2019 to 2040, and use an atmospheric chemistry transport model to estimate their air quality and human health impacts. In 2019, aircraft emissions are estimated to result in 33,900 (95% confidence interval: 23,500–45,600) deaths due to fine particulate matter and 24,600 (15,500–34,200) due to ozone. These two impact metrics increase in three 2040 scenarios of aircraft emissions: by 58% and 82% (low), 119% and 148% (baseline), 169% and 205% (high). Different socioeconomic scenarios for non-aviation emissions change aircraft impacts by up to 44%. These results provide a comprehensive and up-to-date quantification of the global human health burden of aviation and its potential increase in the next two decades.

## Abstract (web summary)

Aircraft emissions can degrade air quality and result in excess deaths due to particulate matter and ozone, according to atmospheric chemistry transport model projections to 2040.

## Introduction

Aircraft emissions contribute to multiple environmental problems including climate change^1^, excessive nitrogen deposition^2^, and air quality^3,4^. Degradation of air quality attributed to aircraft, in the form of higher ground-level concentrations of fine particulate matter (PM_2.5_) and ozone, has been estimated to lead from hundreds to tens of thousands of additional human deaths annually^3,5–8^. The fast pace of aviation’s growth, resulting in 3–4% average yearly increases in global jet fuel use over the past two decades^9^, poses a significant challenge to its environmental sustainability. Currently, pledges of carbon neutrality, generally aiming for the year 2050, take a prominent position in the sector’s outlook for the upcoming decades^10–15^. In these plans, carbon neutral flights are to be achieved largely by the use of sustainable aviation fuels^16^, which does not substantially change NO_*x*_ emissions^17^. As aircraft impact air quality mainly through NO_*x*_ emissions^7,18^, these impacts will increase significantly over time if their sensitivity to emissions remains similar or increases. Potential savings in emissions by shifts to land transportation and by battery electric propulsion are currently limited to short routes and small aircraft, which comprise a small portion of overall emissions^19^. Likewise, hydrogen propulsion is expected to contribute less than 2% of aircraft energy consumption by 2050^16^. Given that aviation is a particularly difficult sector to reduce emissions during the twenty-first century^20,21^, its environmental impacts, including air quality degradation, might also increase in relative importance compared to other human activities.

Aircraft operations can be split into two groups of flight phases: landing and takeoff (LTO), defined as operations up to 3000 ft above ground (an approximation of the planetary boundary layer height), and the remaining (non-LTO) portions of flight. The majority of full-flight emissions (LTO + non-LTO) are released in the upper troposphere and lower stratosphere, impacting air quality at up to intercontinental distances^8^, largely through secondary pollutants that can be transported to the surface far from the initial point of emission. Because of this, the sensitivity of air quality and human health impacts to aircraft emissions is spatially nonuniform and varies with background concentrations that depend on emissions from other sectors, in addition to meteorological conditions. This makes assessing these impacts particularly challenging considering the spatial nonuniformity in temporal changes of air traffic^9^, population distribution^22,23^, other anthropogenic emissions^24^, and climate^25^.

Previous studies have quantified the air quality impacts of aircraft emissions within local^26,27^, regional^8,28–31^, and global scopes^3,5–7,32–37^. The magnitude of results has shown significant variation across different atmospheric models, including both those with online and offline meteorology, and with different methods of modeling chemistry and aerosol microphysics, among other parameters^36^. Some of these studies also quantify the human health impacts associated with this degradation of air quality, with results varying according to what is considered with regards to pollutants (most commonly PM_2.5_, and often ozone), mortality endpoints, and concentration-response functions (CRFs). The effect of these modeling choices is reflected on the range of past estimates of annual excess mortality attributed to aviation. For emissions in 2005 or 2006, mortality has been estimated as (nominal values due to PM_2.5_ | ozone): 9200 | 6400 by ref. ^6^, 13,900 | 2100 by ref. ^3^, and 20,300 | 38,300 by ref. ^8^. A more recent global assessment using different CRFs than adopted here found that 2015 aviation emissions resulted in 21,200 (95% confidence interval, CI: 19,400–22,900) premature deaths due to PM_2.5_ and 53,100 (36,000–69,900) due to ozone^37^.

Comparing aviation’s impacts on air quality between 2006 and 2050, one study found that non-LTO impacts increase over the northern hemisphere by ~150% (PM_2.5_) and ~50% (ozone)^38^, and another found that global mortality associated with PM_2.5_ increases by 465%^35^. Recently, there have been some efforts to evaluate future atmospheric impacts of aviation with a focus on climate^39,40^, but these largely disregard the associated air quality impacts, which have been shown to result in substantial marginal societal damages related to aircraft emissions^41^.

In this study, the GEOS-Chem chemistry transport model is used to quantify the past, present and future global air quality impacts of aviation. We evaluate future impacts for the year 2040 by combining forecasts of aircraft emissions under different market and technology development scenarios, with atmospheric simulations using non-aviation emissions consistent with seven different Shared Socioeconomic Pathways (SSP)^42^. Impacts in 2040 are also evaluated using meteorological inputs from a global climate model under different SSPs.

## Results

### Present-day impacts of aircraft emissions

**Fig. 1:**
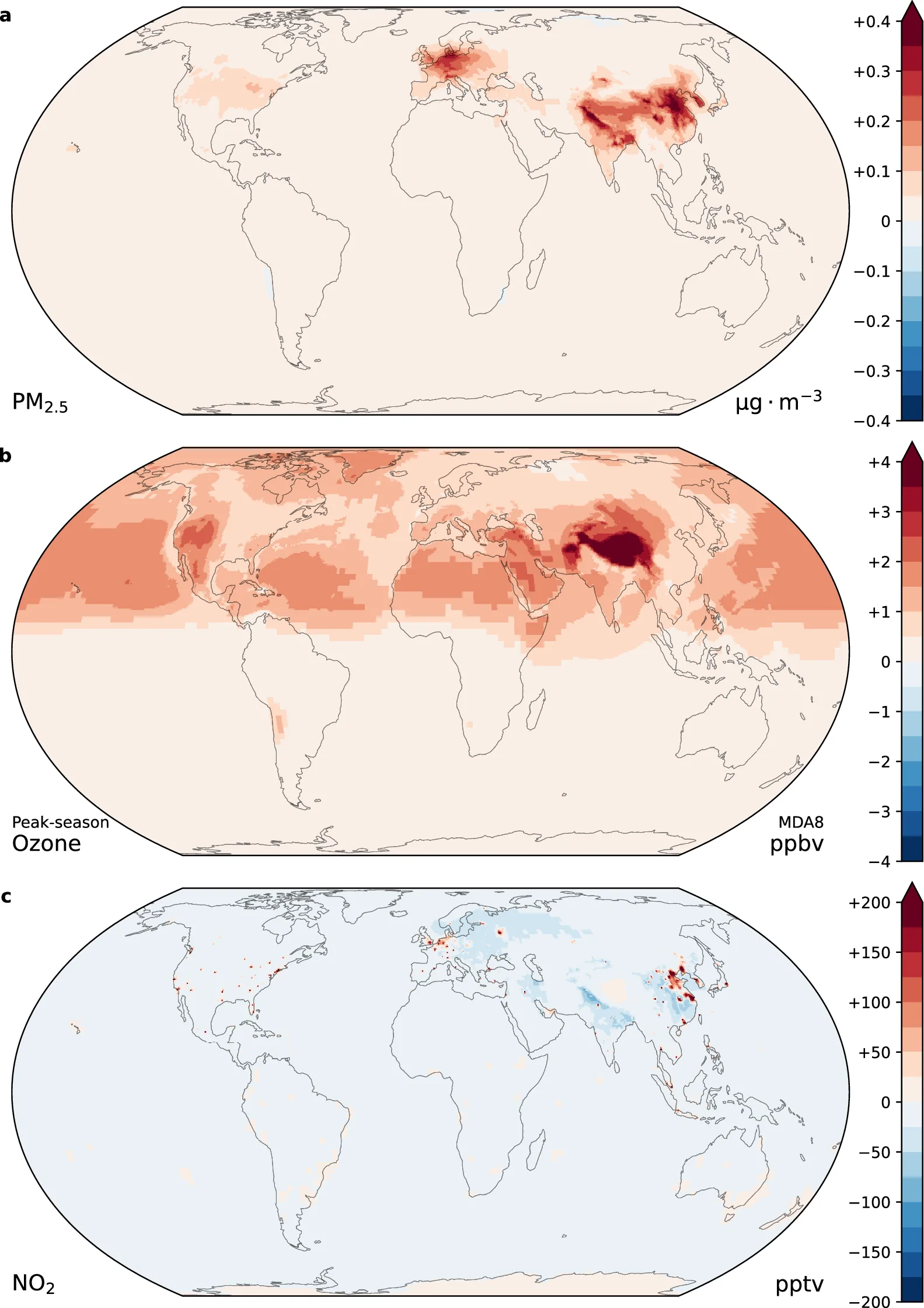
Ground-level changes in 2019 attributed to full-flight emissions. **a** PM_2.5_ mass concentration. **b** Peak-season ozone mixing ratio maximum daily 8-h averages. **c** NO_2_ mixing ratio.

In 2019, full-flight emissions lead to overall increases in annual PM_2.5_ and ozone ground-level concentrations, and both increases near large airports and decreases elsewhere in NO_2_ concentrations (Fig. 1). NO_2_ increases close to emission source and decreases far from it are observed both vertically in the zonal means of full-flight impacts (Supplementary Figs. 14 and 15) and horizontally on the LTO ground-level impacts (Supplementary Fig. 16). Whereas low-altitude NO_*x*_ emissions increase NO_2_ concentrations locally near airports, we find that high-altitude NO_*x*_ largely affects the atmospheric composition at the surface by way of producing ozone, which then drives additional conversion of ground-level NO_2_ into HNO_3_ and nitrate aerosol (Supplementary Figs. 17 and 18). This pattern is observed both in boreal winter and summer (Supplementary Figs. 19 and 20). Such ozone-mediated reductions in ground-level NO_*x*_ due to high-altitude NO_*x*_ emissions have been observed in previous atmospheric modeling studies^6,38^.

Most of the global air quality impacts from full-flight emissions occur in the Northern Hemisphere, where 92% of fuel burn and 87% of the world population are located: 99% (PM_2.5_), 97% (peak-season ozone maximum daily 8-h averages, MDA8), and 95% (NO_2_) of global net increases in population exposure occur north of the equator. Considering area-weighted averages over populated grid cells in the Northern Hemisphere, full-flight emissions increase the PM_2.5_ concentration by 0.050 µg m^−3^, peak-season ozone MDA8 by 1.2 ppbv, and decrease NO_2_ by 8.6 pptv. In the same areas, these aircraft-attributable changes represent 0.2% (PM_2.5_), 2.6% (peak-season ozone), and −0.7% (NO_2_) of (all-source) background pollutant levels, i.e., both due to natural and anthropogenic emissions. The share of pollutant levels attributable to aircraft varies regionally; for example, aircraft emissions are responsible for more than 2% of all-source PM_2.5_ concentrations over large areas in Europe and North America (Supplementary Fig. 21).

The comparison of Northern Hemisphere area-weighted air quality impacts to the population-weighted averages over different regions highlights differences in how pollutants are affected by aviation (Table 1). While ozone impacts are more spatially uniform, PM_2.5_ impacts from aviation are greater in more populated regions, affecting people in Asia more than in the other regions considered, with over 1.5 billion people in Asia experiencing an increase of more than 0.2 µg m^−3^ in annual PM_2.5_ (Supplementary Fig. 22). Globally, 95% of area (Fig. 1) and 71% of population see a decrease in NO_2_ due to full-flight emissions (driven by non-LTO), but large concentration increases in populated regions cause the average net exposure change to be positive (Table 1). NO_2_ average exposure increases significantly more in North America than in the other regions, which is consistent with the greater relative importance of LTO emissions to overall air quality impacts in that region^8^. Globally, aviation represents 0.3% (PM_2.5_), 1.6% (peak-season ozone), and 0.2% (NO_2_) of all-source population exposure.

**Table 1:** Modeled background and aircraft-attributable population-weighted concentrations in 2019

		AS	EU	NA	World
PM_2.5_ annual mean, µg m^−3^	Full-flight	0.163	0.116	0.053	0.112
	LTO-only	0.014	0.021	0.011	0.012
	Background	51.5	18.2	9.3	38.1
Ozone peak-season mean MDA8, ppbv	Full-flight	0.980	1.101	1.082	0.918
	LTO-only	0.022	0.062	0.092	0.032
	Background	62.8	49.9	49.9	56.1
NO_2_ annual mean, ppbv	Full-flight	0.010	0.021	0.049	0.011
	LTO-only	0.017	0.030	0.053	0.018
	Background	6.32	3.37	3.18	4.44

Averages shown for Asia (AS), Europe (EU), North America (NA), and the whole world.

LTO emissions are responsible for 10.9% (PM_2.5_), 3.5% (peak-season ozone MDA8), and 159% (NO_2_) of global exposure attributed to full-flight emissions. The latter means that global exposure is higher in the scenario with only LTO emissions, indicating that non-LTO emissions have a net negative effect on global NO_2_ exposure. The share of full-flight impacts due to LTO emissions is not uniform spatially. For example, in some areas close to large airports half of aircraft-attributable PM_2.5_ is caused by LTO emissions (Supplementary Fig. 23). The seasonality of LTO and non-LTO impacts is also different. Due to lower temperatures and solar radiation, the atmospheric lifetimes of NO_*x*_ released at cruise altitude and its products are longer during winter, which allows them ultimately to affect the surface before they are removed by wet deposition^6^. This causes the average global concentration changes of both PM_2.5_ and ozone due to full-flight emissions to be twice as high during boreal winter compared to summer (Supplementary Fig. 24), while the global net NO_2_ reduction goes from ~0.4 pptv in June-July-August to ~5.4 pptv in December-January-February. Impacts from LTO emissions are less dependent on seasonal differences in tropospheric lifetime.

Increased global annual mean mass of ground-level PM_2.5_ attributed to full-flight emissions consists of 52% nitrate, 23% sulfate, 19% ammonium, 4.5% organics, and 0.6% black carbon (Supplementary Table 8). Only including NO_*x*_ emissions from aircraft in the simulation leads to 97.5% of aircraft-attributed PM_2.5_ being ammonium and nitrate, with the remaining 2.5% being sulfate aerosol formed due to the increased oxidizing capacity of the atmosphere promoting its formation from SO_2_^43^. The omission of aircraft emissions of SO_*x*_, hydrocarbons, CO, and nonvolatile particulate matter (nvPM) in the simulation reduces global annual PM_2.5_ changes by 30% (area-weighted) and 13% (population-weighted). NO_*x*_ is responsible for almost all of ozone and NO_2_ impacts, such that these impacts (averaged globally) change by less than 1% by the inclusion of non-NO_*x*_ emissions from aircraft.

**Fig. 2:**
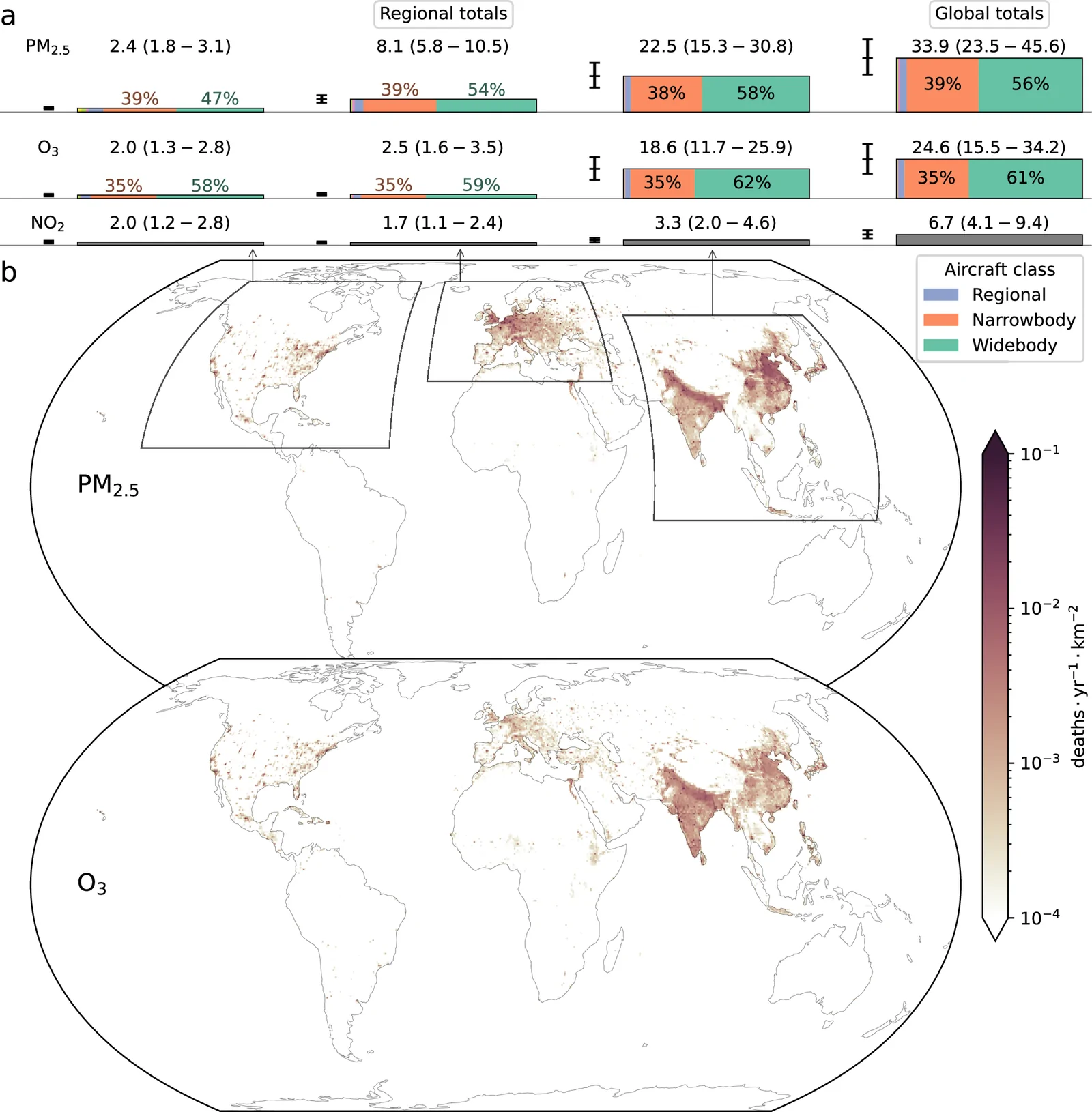
Modeled excess mortality attributed to aircraft full-flight emissions in 2019. **a** Mortality in thousands per region, pollutant, and aircraft class (for PM_2.5_ and ozone). Values within parentheses are the 95% confidence interval considering the uncertainty associated with each CRF. **b** Mortality from PM_2.5_ and ozone per area from all aircraft classes.

Globally, we find that aircraft emissions in the year 2019 are associated with a total excess mortality of 33,900 (95% CI: 23,500–45,600) due to PM_2.5_, 24,600 (15,500–34,200) due to ozone, and 6,700 (4,100–9,400) due to NO_2_, as shown in Fig. 2. Mortality estimates are larger compared to previous studies^3,5,6,37^, except for ozone in ref. ^37^, due to the combined effect of increases in aircraft emissions, population count, and modeled ground-level ozone sensitivity to NO_*x*_ emissions, as well as the CRF considered. Health impacts calculated using alternative CRFs are presented in Supplementary Tables 9–11. As an example, using the same ozone CRF as Eastham et al.^37^, increases our estimate of ozone mortality due to full-flight emissions by 142%, making our estimate 12% larger than theirs. Therefore, the choice of CRF can also significantly affect the share of overall impacts attributed to each pollutant; while the ratio between PM_2.5_ and ozone aircraft-attributable mortality in our main estimate for 2019 is 58:42, this ratio was 87:13 in the estimate by Yim et al.^3^, 60:40 in that by Eastham and Barrett^6^, and 29:71 in Eastham et al.^37^. 66% (PM_2.5_) and 76% (ozone) of aircraft-attributable mortality estimated here for 2019 occurs in Asia, where 57% of the global population over age 30 is located. Emissions from LTO are responsible for 12.5% (PM_2.5_) and 3.3% (ozone) of total mortality attributed to full-flight emissions, thus having a greater health impact associated with PM_2.5_ per mass of fuel burned than non-LTO emissions, and smaller with ozone. LTO emissions lead to a decrease in ozone during winter near regions of large aircraft emissions, which is associated with conditions changing into an ozone production regime limited by volatile organic compounds^8^. If ozone health impacts are estimated using a CRF considering annual concentration averages instead of peak-season averages, such as the relative risk of death by chronic obstructive pulmonary disease (COPD) of 1.14 per 10 ppbv found by Turner et al.^44^, a lower fraction of global aircraft-attributable ozone mortality is due to LTO: 2.0% instead of 3.3%. This difference highlights the fact that LTO impacts on ozone are in phase with the seasonal variation of background surface concentration, i.e., higher during peak ozone season, while non-LTO impacts are larger during winter, when concentrations are lower. Mortality associated with NO_2_ (Supplementary Fig. 25), which has not been evaluated in previous studies of global aviation impacts, is estimated here as 10% the sum of mortality from PM_2.5_, ozone, and NO_2_ attributed to full-flight emissions, but NO_2_ represents 63% the mortality sum associated with LTO emissions, highlighting its potential importance at local scales.

We find that widebody aircraft operations are responsible for 56% (PM_2.5_) and 61% (ozone) of global aviation-attributable premature mortality, and narrowbody aircraft for 39% (PM_2.5_) and 35% (ozone), as shown in Fig. 2. The share of all aircraft NO_*x*_ emitted by widebodies and narrowbodies match the share of ozone mortality: 61% and 35%, respectively. Although some regional variation exists, these aircraft categories are associated with the majority of impacts in all regions evaluated. We estimate that 12% (PM_2.5_) and 9% (ozone) of all global aircraft-attributable mortality is associated with flights shorter than 750 km.

### Future emissions scenarios

**Fig. 3:**
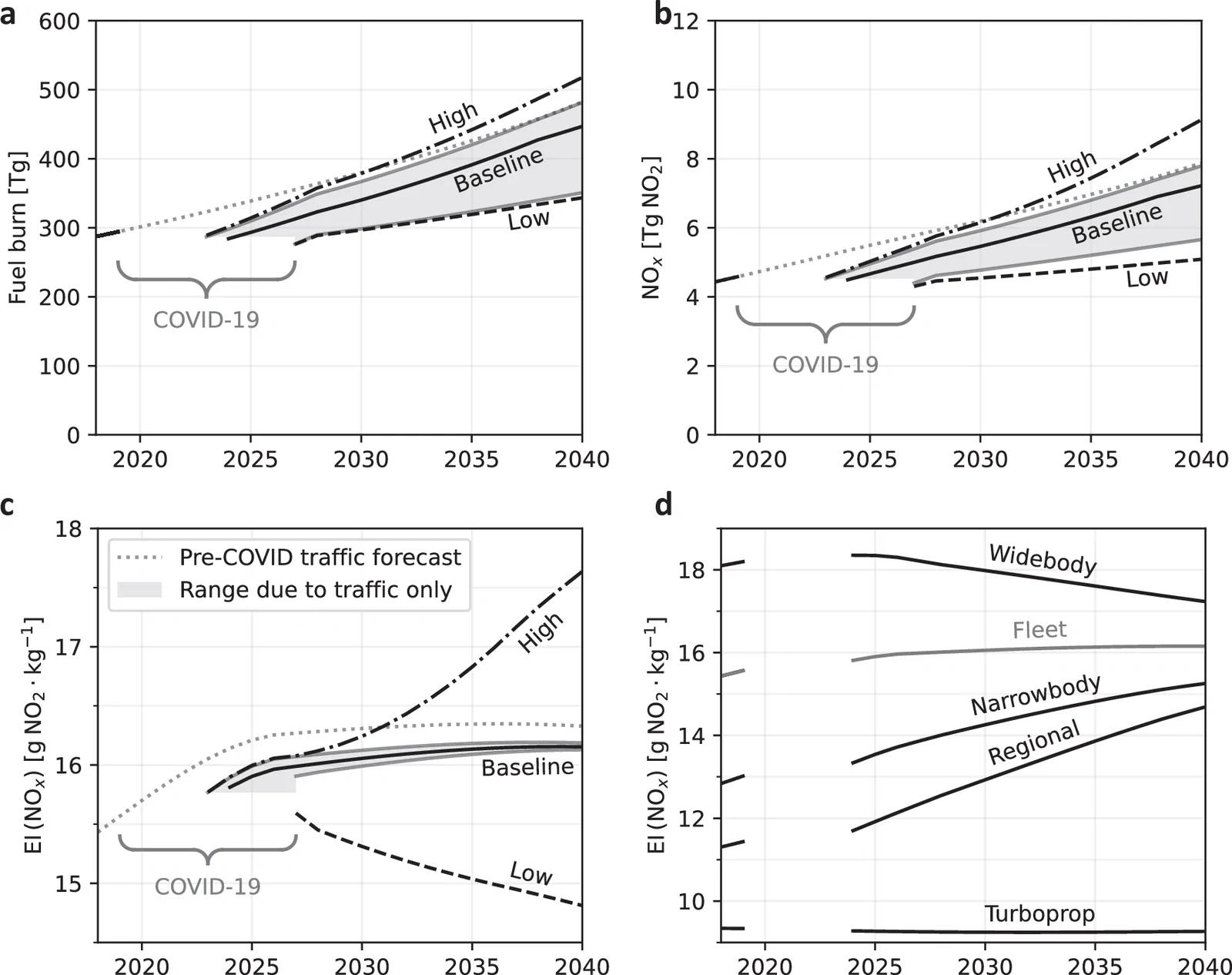
Projected changes in global aircraft fuel burn and NO_*x*_ emissions. **a** Annual fuel burn mass. **b** NO_*x*_ emissions mass. **c** Fleet average NO_*x*_ emission index. **d** Average EI (NO_*x*_) per aircraft class for the baseline scenario. In (**a**–**c**), curves for the three overall scenarios considered are shown with black lines, curves for different traffic scenarios combined with baseline parameters are shown with solid gray lines closer to the baseline and with the dotted gray line for the Pre-COVID traffic forecast.

**Fig. 4:**
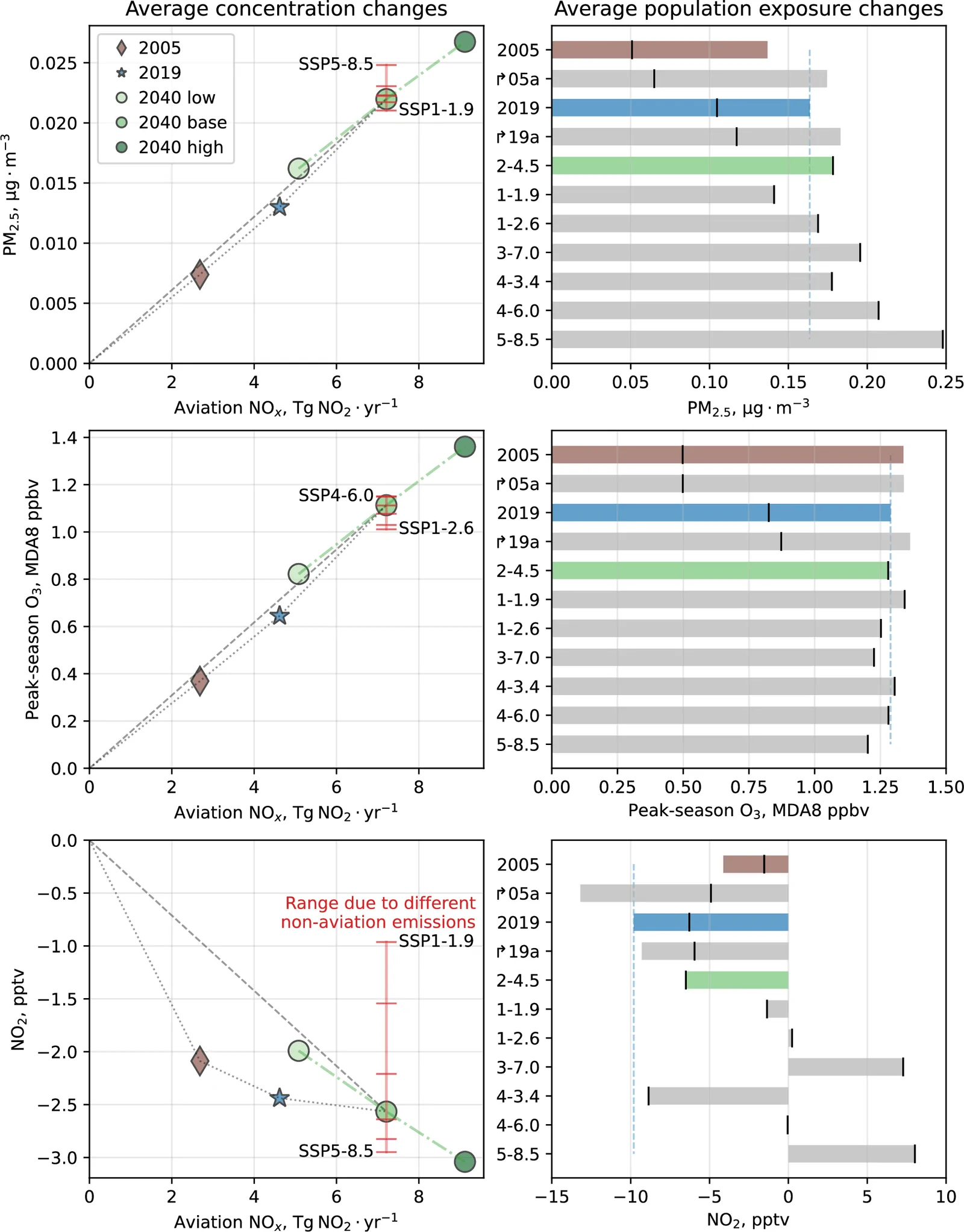
Sensitivity of global air quality to full-flight emissions. Left: global annual area-weighted mean full-flight attributable ground-level pollution versus NO_*x*_ emitted. Future estimates indicated by green circles consider SSP2-4.5 non-aviation emissions. Gray and green lines connecting the markers are shown to highlight changes in sensitivity. Right: global annual population-weighted mean ground-level concentrations attributable to full-flight emissions for different socioeconomic scenarios. Future socioeconomic scenarios all utilize the baseline 2040 aircraft emissions. Filled horizontal bars indicate values normalized according to the amount of aviation NO_*x*_ to 2040 levels; unnormalized values are indicated by black vertical dashes. “05a” and “19a” combine 2019 non-aviation with 2005 aircraft emissions, and 2040 (SSP2-4.5) non-aviation with 2019 aircraft emissions, respectively. 2019 results consider only the same resolution as the other simulations, excluding nested domain simulations at finer resolution.

We estimate that global aircraft NO_*x*_ emissions and average emission indices (EI, shown between parentheses) will change between 2019 and 2040 by +10% (−4.9%), +56% (+3.8%), and +97% (+ 13.3%) in the low, baseline, and high projection models, respectively (Table 2 and Fig. 3). This follows the realized increases of 72% (+4.6%) between 2005 and 2019. On the global average, surface aircraft-attributable PM_2.5_ and ozone concentrations increase approximately in proportion to the mass of aircraft NO_*x*_ emissions in the perturbation (Fig. 4), with sensitivity decreasing as aircraft NO_*x*_ emissions go from the “low” to the “high” scenario by 8% (PM_2.5_ and ozone) and 15% (NO_2_). Across different future socioeconomic scenarios for non-aviation emissions, mean concentration changes due to full-flight emissions vary relative to SSP2-4.5 from −4% to +13% for PM_2.5_, and from −9% to +3% for ozone. NO_2_ net decreases, on the other hand, have lower sensitivity to full-flight aircraft emissions in the future than in 2019, which in turn have decreased sensitivity compared to 2005. Relative to the other pollutants, there is also more spread in NO_2_ mean concentration changes depending on future scenarios: from −62% to +15% relative to SSP2-4.5.

**Table 2:** Annual global aircraft full-flight fuel burn and emissions

Year	Scenario	Fuel [Tg]	NO_*x*_ [Tg NO_2_] ([g NO_2_ kg^−1^])	SO_*x*_ [Gg S] ([µg S kg^−1^])	Hydrocarbons [Gg CH_4_] ([g CH_4_ kg^−1^])	CO [Tg] ([g kg^−1^])	nvPM [Gg] ([mg kg^−1^])
2005	Historical	181	2.69 (14.9)	108 (600)	187 (1.04)	0.75 (4.13)	5.9 (32.8)
2019	Historical	297	4.62 (15.6)	178 (600)	43 (0.14)	0.81 (2.74)	9.7 (32.6)
2040	Low	343	5.09 (14.8)	206 (600)	44 (0.13)	0.76 (2.23)	11.8 (34.5)
2040	Baseline	447	7.22 (16.2)	268 (600)	57 (0.13)	0.98 (2.21)	15.4 (34.6)
2040	High	518	9.13 (17.6)	311 (600)	66 (0.13)	1.13 (2.20)	17.8 (34.5)

Values are given for bottom-up inventories for 2005 and 2019, and three projection scenarios for 2040. Average emission indices are shown in parentheses.

As can be seen in the plots on the right of Fig. 4, mean population exposure also increases with the amount of full-flight NO_*x*_ emissions. From 2005 to 2019, exposure sensitivity to the amount of aircraft NO_*x*_ increases for PM_2.5_ (20%) and NO_2_ (137%), and decreases for ozone (4%). From 2019 to 2040 SSP2-4.5, exposure sensitivity changes by +9% (PM_2.5_), −1% (ozone), and −34% (NO_2_). Normalized exposure for all three pollutants increases when changing just aircraft emissions to those of a previous year (“05a” vs. “2019”, and “19a” vs. “(SSP)2-4.5” in Fig. 4). These changes in sensitivity might not only be caused by nonlinearity in the atmospheric response, but also due to the fact that the scenarios with more aircraft emissions have a higher percentage of those emissions over Asia and lower over Europe, the latter having a stronger impact on pollutant exposure^8^. Across non-aviation emissions corresponding to different future socioeconomic scenarios, the change in exposure sensitivity to aircraft NO_*x*_ relative to 2019 ranges from −14% to +52% (PM_2.5_), and from −7% to +4% (ozone). While for NO_2_ the global average concentration changes are negative in all scenarios, due to countering effects of NO_2_ increases in population-dense areas near airports and decreases far from them, the global average net exposure associated with full-flight emissions is positive in 3 of the 7 future scenarios (Fig. 4). Notably, while net exposure to NO_2_ in 2019 is positive when including the higher resolution simulations over the nested domains (Fig. 2), it is negative when only considering the coarser resolution global simulations (Fig. 4). Higher resolutions are better able to capture near-airport conditions, where typically population density is higher and low-altitude aircraft emissions increase NO_2_ concentrations, leading to higher estimates of LTO NO_2_ impacts, potentially tipping the balance towards a global net positive change in population exposure.

The sensitivities of aircraft-attributable global exposure to aircraft LTO-only emissions (Supplementary Fig. 26) change from 2019 to SSP2-4.5 2040 by −58% (PM_2.5_), +36% (ozone), and +8% (NO_2_). Opposite to full-flight impacts, different non-aviation emission scenarios affect more the LTO impacts on PM_2.5_ and ozone, and less the impacts on NO_2_. Relative to the baseline 2040 scenario (SSP2-4.5), global sensitivities of ozone and NO_2_ exposure to LTO emissions increase in the less polluted scenario (SSP1-1.9) by 43% and 9%, respectively, and decrease in the more polluted scenario (SSP3-7.0) by 28% and 6%, in the same order. Both alternative future socioeconomic scenarios have more PM_2.5_ exposure attributed to LTO (25% in SSP1-1.9, and 127% in SSP3-7.0). These net global changes are not uniform spatially, particularly for PM_2.5_, with different regions experiencing either higher or lower sensitivity to aircraft emissions across the three scenarios (Supplementary Fig. 27). Similarly, there is also spatial variability in the sensitivities to full-flight emissions across different socioeconomic scenarios for 2040 non-aviation emissions (Supplementary Fig. 28). For example, in the less polluted scenario (SSP1-1.9), PM_2.5_ sensitivity to full-flight emissions is lower in India but higher in China compared to SSP2-4.5. Also compared to this SSP2-4.5 scenario, in the more polluted future scenario (SSP3-7.0), ozone sensitivity is higher by less than 5% in most populated areas in the Northern Hemisphere, but lower by more than 20% or 30% in areas in eastern Asia. We note that these changes in sensitivities are the net result of emissions of different species, changing at different rates (and possibly signs) in different locations, together with the resulting effects of atmospheric transport on their impacts.

Aircraft-attributable mortality in the year 2040, considering the baseline emissions scenario with SSP2-4.5 population and non-aviation emissions, are estimated as 68,000 (95% CI: 47,100–91,900) deaths from PM_2.5_ and 54,100 (34,100–75,100) from ozone (Table 3). These respectively represent 119% and 148% global increases relative to 2019 when simulated at the same resolution (i.e., without incorporating the higher resolution simulations available only for 2019). This is the result of the combined effects of a 56% increase in aircraft NO_*x*_ emissions, a 13% increase in global population over age 30, increases in globally-averaged baseline mortality rates of 22% (non-communicable diseases and lower respiratory infections) and 49% (COPD), and a similar sensitivity of ground-level pollution to aircraft emissions. For the same future population and non-aviation emissions, changing only aircraft emissions from 2019 to 2040 (baseline) increases global PM_2.5_ and ozone mortality by 49% and 47%, respectively. For the same non-aviation emissions scenario, the “low” and “high” aircraft emissions projections, in this order, result in annual deaths of 49,200 (34,100–66,400) and 83,800 (58,000–113,300) from PM_2.5_, and 39,600 (25,000–55,100) and 66,300 (41,900–92,100) from ozone. These represent increases of 58% and 169% (PM_2.5_) and 82% and 205% (ozone) relative to 2019. Non-NO_*x*_ aircraft emissions contribute the same share of full-flight health impacts in 2019 and 2040 (SSP2-4.5): their exclusion in the simulations reduces PM_2.5_ impacts by 14%, while impacts from ozone and NO_2_ are not significantly affected (Supplementary Table 12). Changing population and non-aviation emissions from the baseline SSP2-4.5 to each of the other six socioeconomic scenarios results in aircraft-attributable mortality changes between −6% and +44% for PM_2.5_ and −5% and +14% for ozone. If population and baseline mortality rates are kept the same as SSP2 and only non-aviation emissions are changed, aircraft-attributable PM_2.5_ and ozone mortality change by a narrower range of −7% to +6%, highlighting the potential importance of socio-economic factors for such estimates, consistent with previous observations on projections of health impacts of all-source air pollution^45^. Aircraft-attributable health impact estimates using alternative future baseline mortality rates are presented in Supplementary Tables 13 and 14. Of the two alternative LTO-only perturbation future scenarios, SSP1-1.9 results in the largest change in impacts relative to the baseline, with mortality increasing by 57% for PM_2.5_ and by 66% for ozone.

**Table 3:** Annual global premature mortality attributed to aircraft emissions for different scenarios

Population and non-aviation emissions	Aircraft emissions	Early deaths (×10^3^)		
		PM_2.5_	Ozone	NO_2_
2005	2005	12.6	11.1	−0.3
2019	2005	19.5	13.1	−1.5
2019*	2019*	31.1*	21.8*	−1.9*
2019†	2019†	33.9†	24.6†	6.7†
SSP2-4.5	2019	45.7	36.7	−1.2
SSP2-4.5	2040	68.0	54.1	−0.9
SSP2-4.5	2040 low	49.2	39.6	−1.0
SSP2-4.5	2040 high	83.8	66.3	−0.8
SSP1-1.9	2040	74.4	61.9	2.3
SSP1-2.6	2040	82.7	56.9	4.8
SSP3-7.0	2040	66.8	53.0	7.6
SSP4-3.4	2040	63.8	52.7	−2.2
SSP4-6.0	2040	65.6	51.2	3.7
SSP5-8.5	2040	97.9	53.4	8.5
2019*	2019 LTO-only*	9.7*	1.1*	3.9*
2019†	2019 LTO-only†	4.2†	0.8†	8.7†
SSP2-4.5	2040 LTO-only	11.5	3.6	8.9
SSP1-1.9	2040 LTO-only	18.0	6.1	10.7
SSP3-7.0	2040 LTO-only	17.4	2.3	8.4

Negative values indicate higher mortality when aircraft emissions are excluded. To allow for comparison, for the two 2019 scenarios also simulated at higher resolution with nested domains, both the values from solely the coarse global simulations (marked with *), which are more directly comparable to the rest of the table, and the values including the higher resolution results (marked with †) are shown.

### Different meteorological scenarios

The impact of different future climate scenarios is evaluated with atmospheric simulations driven by SSP-specific meteorological fields for 2040 from the Global Change and Air Pollution framework (GCAP 2.0)^46^ generated by the global climate model E2.1 from the NASA Goddard Institute for Space Studies (GISS)^47,48^, as opposed to the 2005 and 2019 fields from the MERRA-2 reanalysis product used in the simulations discussed in the previous sections. For this, the scenarios SSP1-2.6, SSP2-4.5, and SSP5-8.5 are considered, covering a wide range of expected radiative forcing. The resulting global PM_2.5_ exposure attributable to the same 2040 baseline full-flight emissions is lower relative to the scenarios using 2019 meteorology: −49% for SSP1-2.6, −51% for SSP2-4.5, and −61% for SSP5-8.5. Aircraft-attributable global peak-season ozone exposures differ between the two by −2% to +2%, and NO_2_ exposures amount to larger net global decreases in GCAP 2.0. This suggests that in GCAP 2.0 for 2040, full-flight aircraft emissions affect ground-level air relatively more in the form of ozone compared to MERRA-2 for 2019, which also tends to lead to a decrease of the NO_2_ concentration.

**Fig. 5:**
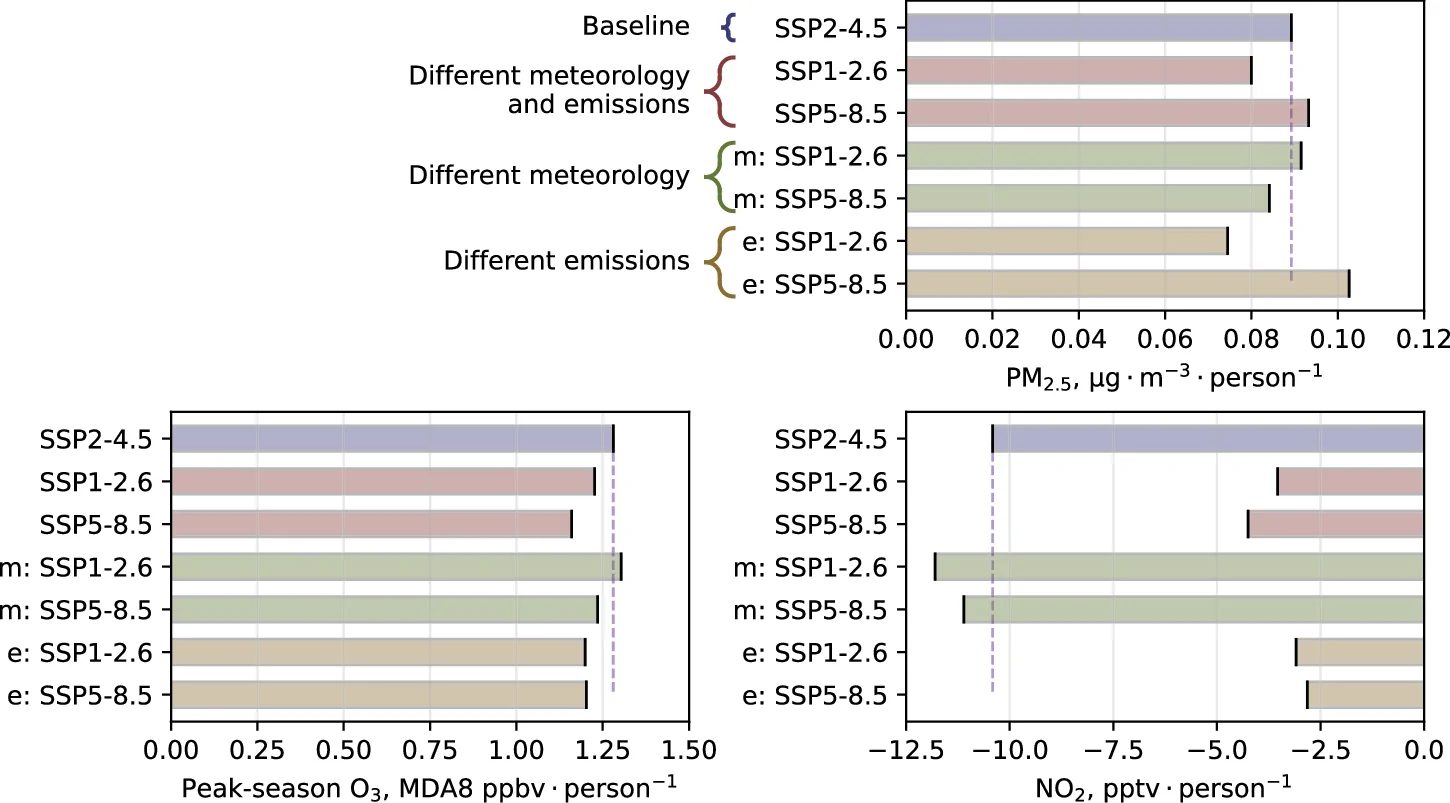
Exposure attributed to full-flight emissions in 2040 for different socioeconomic scenarios of non-aviation emissions and meteorological fields from GCAP 2.0. Full-flight attributable global mean exposure of PM_2.5_, peak-season ozone, and NO_2_. For all cases, SSP2 population for the year 2040 is used.

Figure 5 shows how different SSP scenario non-aviation emissions and resulting meteorology, consistently within the GCAP framework, impact global full-flight attributable exposure for PM_2.5_, ozone, and NO_2_: the effect of switching only meteorology fields (exposure magnitude changes up to 6%, 4%, 13% for PM_2.5_, ozone, and NO_2_, respectively) is smaller than that of switching only non-aviation emissions (exposure magnitude changes up to 17%, 6%, and 73%, in the same order). Different meteorology scenarios change background exposure (from all sources) to the three pollutants by up to 3% relative to SSP2-4.5, which means that the effect of meteorology is greater on the impacts from aircraft emissions compared to other sources, as is expected from their higher release altitude. We note that the combined impacts of non-aviation emissions and meteorology (again using global exposure due to full-flight emissions as the metric) are in the expected direction of the combination of their impacts evaluated separately: when changing just meteorology increases impacts relative to the baseline, then the combined effects are larger than just changing emissions, and vice versa.

## Discussion

The quantification of human health effects from aircraft emissions presented here is further evidence that the increase of NO_*x*_ emissions in this sector, of 72% between 2005 and 2019, and an expected further 56% to 2040, is accompanied by a corresponding increase in human health impacts. In fact, as the results for different future scenarios show, the combined effects of spatially nonuniform changing population counts and age structure, non-aviation emissions, and aircraft emissions can lead to increases in the sensitivity of global exposure to aircraft emissions, such that health impacts from aviation increase faster than emissions. Uncertainty in the CRF propagates to the health impacts of aviation, so care must be taken when considering the absolute magnitude of impacts, as well as in their monetary valuations, which are necessary for comprehensive environmental mitigation efforts^41,49^. However, the relative evolution of these impacts over time in our study would not be affected by a systematic bias in the CRF. Additionally, the absolute numbers presented reflect the modeled atmospheric changes resulting from the elimination of all aircraft emissions. Given the non-linearities of the processes involved, our modeling atmospheric changes, that reflect the complete removal of aircraft emissions, may not necessarily scale linearly (beyond first order) to realistic mitigation scenarios, where emissions are partially reduced. In a previous assessment of the sensitivity of air quality to aircraft emissions in GEOS-Chem (SI of ref. ^8^), it was found that sensitivities were lower (particularly of ozone) for larger perturbations in emissions, such that we would expect the marginal impacts per mass of emissions of small changes to be larger than those estimated here.

The share of full-flight health impacts due to non-LTO emissions varies significantly by pollutant: for 2019, we find it to be 87% for PM_2.5_ and 97% for ozone, such that the overall average depends to a great extent on the choice of CRF. These values compare to the combined PM_2.5_ + ozone share of 75% found in ref. ^3^, and 80% in ref. ^5^. LTO impacts might be underestimated here due to not considering other ground sources of aviation-related emissions, not considering the potentially higher toxicity of black carbon and ultrafine particles among PM_2.5_^50–53^, and due to limitations of the global atmospheric model in resolving individual urban areas and capturing effects of emission co-location^3^. Full-flight mortality estimates for 2019 are higher when including higher resolution nested simulations (Table 3), in line with previous studies that used GEOS-Chem^8,37^, but LTO-attributable PM_2.5_ and ozone mortality are lower, indicating their larger sensitivity to local background concentrations. The global aircraft impacts associated with long-term NO_2_ exposure were also quantified in the present study, and while non-LTO impacts are negative (the inclusion of these emissions in the simulation leads to reductions in mortality), within LTO impacts, NO_2_ leads to a comparable amount of mortality compared to PM_2.5_ and ozone for the CRFs chosen. Overall for NO_2_, we note the dependency on model resolution, with higher impacts when the nested simulations are included, and high sensitivity to changes in non-aviation emissions. As with a previous estimate that NO_2_ contributes 91% of LTO-attributable mortality in the United States^30^, our results suggest that impacts on this pollutant, particularly from LTO emissions, should be investigated further. A growing body of evidence from meta-analyses of epidemiological studies indicate that long-term exposure to ozone and NO_2_ might have negative health impacts independent from PM_2.5_ and each other^54,55^, although a single multi-pollutant metric for mortality quantification is still uncertain.

Assuming that air traffic increases as projected and aircraft technology continues present trends of development, it is likely that global aircraft NO_*x*_ emissions will be significantly higher in 2040 than in 2019. In our baseline scenario for the year 2040, which does not consider potential mitigating effects of expanded biofuel usage or aircraft not powered by hydrocarbon fuel, aircraft emissions are responsible for 68,000 (47,100–91,900) annual deaths from PM_2.5_ and 54,100 (34,100–75,100) from ozone, respectively 119% and 148% increases over the estimate for 2019 using the same model resolution. And as other transportation sectors have more viable solutions to reduce emissions^20^, impacts from aviation are likely to also increase in relative terms: the fraction of modeled population exposure due to aircraft emissions increases between 2019 and 2040 (baseline scenario) from 0.28% to 0.47% for PM_2.5_, and 1.5% to 2.2% for peak-season ozone (MDA8). The dependence of future air quality impacts from aircraft emissions on future population changes and emissions from other sectors is apparent in the results presented, as exemplified by global PM_2.5_ mortality being 44% higher for the same aircraft full-flight emissions when released in an SSP5-8.5 scenario instead of the baseline SSP2-4.5, and by the LTO-attributable mortality being 57% (PM_2.5_) and 66% (ozone) higher in the SSP1-1.9 scenario compared to SSP2-4.5.

The air quality impacts associated with aviation emissions are likely to remain an environmental concern for the coming decades. Potential carbon-free aircraft propulsion technologies can more easily be developed first on smaller classes of aircraft^12^. Technological and market-based efforts to displace short-distance flights, such as battery electric aircraft or other modes of transportation, would likewise have limited impact, as we estimate that flights shorter than 750 km account for 12% (PM_2.5_) and 9% (ozone) of all global aircraft-attributable mortality. Current proposals for achieving carbon neutrality in the larger aircraft classes rely primarily on extensive use of biomass-derived or synthetic kerosene, which do not significantly impact NO_*x*_ emissions, but may lead to a reduction in SO_*x*_ and primary particulate matter emissions, depending on the specific technologies employed^17,56,57^. As modeled here, sulfate, organic aerosol, and black carbon represent 23%, 4.5%, and 0.6% of the total mass of ground-level aircraft-attributable PM_2.5_ (Supplementary Table 8). Comparison of our simulations including and excluding aircraft non-NO_*x*_ emissions suggests that, for both 2019 and 2040, NO_*x*_ alone causes 86% of PM_2.5_ and more than 99% of ozone mortality attributed to full-flight emissions of all species (Supplementary Table 12), which is in line with a previous finding that ultra-low sulfur jet fuel could reduce up to 17% of global aviation-attributed PM_2.5_ mortality^7^. Based on those results, technologies that prioritize NO_*x*_ emissions reduction would have the largest impact in reducing aviation’s air quality impacts. Most of these benefits would originate from cruise-level aviation NO_*x*_ emissions, which are not explicitly included in the regulated emissions presently. However, if higher toxicity of black carbon or ultrafine particles is considered instead of a single CRF for total mass of PM_2.5_, then the relative importance of non-NO_*x*_ emissions would be higher. In that case, the estimated health benefits of replacing jet fuel with an alternative that lowers emissions of black carbon and ultrafine particles would be larger. Reductions in particulate matter emissions from alternative fuels are dependent on the specific properties of the fuel and the engines burning it^56–58^, therefore, estimates of the consequent changes on air quality and human health impacts would require further considerations. The relative importance of directly emitted (primary) particulate matter compared to NO_*x*_ emissions is larger for air quality at local scales than at global scale, such that measures reducing non-NO_*x*_ pollutants can have more significant local impacts than suggested by global-scale simulations.

We find that impacts of changes in meteorology between different SSP scenarios are lower than those due to changes in non-aviation emissions, suggesting that the might not be a significant contributor to uncertainty in estimating future scenarios of aircraft air quality impacts, at least for the ~20-year future timescale. However, compared to population exposure to all-source background pollution, aircraft-attributable exposure is more affected by meteorology changes, with variations of up to 3% across the three pollutants. Changes in atmospheric climate are also expected to continue to increase beyond 2040^25^, so these effects might become more significant in a longer timescale. We note that air quality sensitivity to aircraft emissions is shown here to be sensitive to the source of meteorological data used. Particularly, using meteorology generated from a general circulation model (GCAP 2.0) instead of a reanalysis model (MERRA-2) yields around 50–60% lower sensitivity of global population PM_2.5_ exposure to full-flight emissions. Additionally, the atmospheric model used here simulates aerosol microphysics in a simplified manner (described by refs. ^59,60^) and is driven by prescribed meteorology, such that the interaction between clouds and aircraft exhaust (and the secondary pollutants formed from it) is not considered, and the feedback mechanisms between meteorology and aerosol formation are not captured.

## Methods

The GEOS-Chem model version 13.3.3 is used to quantify the impacts of aircraft emissions in the atmosphere at a global scale. Simulations are performed with and without aircraft emissions, and the difference in air quality across the simulation runs is taken as aviation’s contribution to ground-level air pollution. The scope of this study includes only emissions from fixed-wing civil aircraft, so the terms “aviation” and “aircraft” are used interchangeably to refer to them. In some scenarios, aircraft emissions from LTO are evaluated separately to isolate their contribution to impacts from full-flight emissions. Likewise, as a way of estimating how much of the overall impacts of full-flight emissions could be attributed more specifically to NO_*x*_, we perform simulations excluding only aircraft emissions of species other than NO_*x*_. Human health impacts are quantified as the expected increase in mortality associated with long-term exposure due to changes in the surface-level concentrations of PM_2.5_, ozone, and NO_2_.

Three sets of scenarios are considered, with different aviation emissions, non-aviation emissions, and meteorological fields. Firstly, “present day” impacts on air quality and human health from aircraft emissions from different aircraft categories are investigated by performing simulations of the year 2019, as the most recent representative year of aviation activity before the COVID-19 pandemic. Secondly, the effects of the evolution of both aviation and non-aviation emissions are evaluated by comparing results from simulations for the years 2005, 2019, and 2040. And thirdly, simulations using different meteorological fields for the year 2040 are compared in order to evaluate the sensitivity of aviation’s impacts on air quality to a range of atmospheric conditions representative of possible future SSP scenarios.

Each of the seven future socioeconomic scenarios is labeled as “SSP*x*-*y*”, where *x* indicates one of five socioeconomic pathways associated with a specific narrative that drives trends in emissions, population, and climate (among other things)^42^; and *y* is an approximation of the radiative forcing in the year 2100 (in W m^−2^) for that scenario. While a higher “*y*” is generally indicative of more greenhouse gases being emitted, each scenario has different trends of emissions over time for different pollutants. Riahi et al. offer a summary of each SSP narrative^42^, and the overall evolution of emissions over time for each scenario is presented by Murray et al.^46^. Within the context of this study, we consider SSP2-4.5 as a baseline socioeconomic scenario, while SSP1-1.9 and SSP1-2.6 have broadly lower emissions, SSP3-7.0 and SSP4-6.0 have broadly higher emissions, and SSP4-3.4 and SSP5-8.5 have mostly higher emissions with emissions of some species being lower than in SSP2-4.5. Note that, separately to a baseline within socioeconomic scenarios (SSP2-4.5), we also consider a baseline within the three future aircraft emission scenarios evaluated, as described in the next subsection.

### Aircraft emissions

Civil aircraft emissions for all simulated years are modeled as three-dimensional fields of monthly-averaged emission rates of NO_*x*_ (as NO_2_), CO, hydrocarbons (speciated according to the U.S. EPA’s profile #5565^61^), SO_*x*_ (based on a constant 600 ppm fuel sulfur content), nonvolatile particulate matter mass (as hydrophilic black carbon and organic carbon aerosol). Emission rates for 2019 are calculated from the openAVEM bottom-up inventory^62^ described in ref. ^9^, totaling 4.62 Tg of NO_*x*_ (in base NO_2_) over the year. Fuel burn estimates are based on average time-in-mode values for LTO^63^, and on the BADA aircraft performance model^64^ for the non-LTO portions of flights. Emission indices (EI, mass ratio of emission and fuel burned) are calculated using data from the Engine Emissions Databank maintained by ICAO (International Civil Aviation Organization)^65^, according to the Boeing Fuel Flow Method 2^66^ and FOA 4 methods^67,68^. Following the methods and engine assignments presented in ref. ^9^, emission indices of jet airliners are calculated for each aircraft type by adopting one representative engine model number for each engine family; and, in case of multiple options available (e.g., CFM LEAP and PW1100G for type A20N), taking an average of values between families weighted by an estimate of their market share. Emission rates for 2005 are calculated in the same manner, using data published by Simone et al.^69^. For both years, only emissions from aircraft’s main engines and auxiliary power units are considered, without taking into account emissions from ground support equipment, tire and brake dust, and emissions from airport operations.

**Fig. 6:**
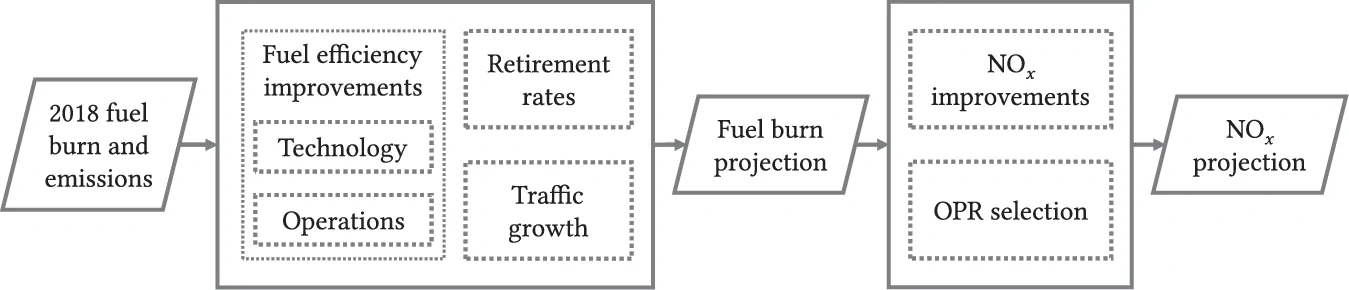
Overview of the process of projecting fuel burn and NO_*x*_ emissions. The diagram illustrates the first iteration of the projection, which is then repeated annually until 2040, for both passenger and cargo operations.

Aircraft NO_*x*_ emissions for the year 2040 are calculated using a method based on ref. ^70^, for three scenarios of emissions: “low”, “baseline”, and “high”. While “baseline” is intended to represent a likely future, “low” and “high” have the goal of establishing a range of plausible outcomes instead of representing specific pathways of technological evolution with low or high emissions. As such, they consider parameter choices that simultaneously reduce or increase emissions (such as low fuel efficiency improvements and high engine overall pressure ratios), even though some technological pathways would have opposing effects on fuel efficiency and NO_*x*_ EI. This approach with separate parameters for the projection is based on the methodology used by Cumpsty et al.^71^. An overview of how the emission projections are produced is given in Fig. 6. The relevant factors in the projections are described in more detail in the Supplementary Note 1, which also contains an evaluation of the sensitivity of the projection to the choice of values for those factors.

The starting point of the projection is 2018, as traffic data with scheduled seats or freight capacity per flight was only available for that year, and the ICAO traffic forecasts used have 2018 as their starting point^72^. Scheduled flight data used for 2018 was obtained from market intelligence company OAG. The traffic growth rates applied in the first iteration are such that the global sum of traffic for 2019 matches the values reported by the International Air Transport Association. This calculation pipeline is performed twice in parallel, once for passenger services, and another for cargo services. In this setup, “cargo” includes exclusively freight and mail services, while mixed services that carry both passenger and cargo are included in the “passenger” part of calculations. For cargo flights, only three classes of aircraft are considered, with the omission of a regional jet class.

Aircraft types are grouped into the classes of widebody (twin aisle), narrowbody (single aisle), regional jet, and turboprop based on form and engine type. Transport jet aircraft not based on a passenger aircraft, which constitute less than ~0.1% of global fuel burn, are classified ad hoc as widebody or narrowbody for this analysis, according to their size. Emissions from business jets and piston engine aircraft, which are estimated to contribute ∼1–2% of global fuel burn^9^, are not included in this analysis of future emissions due to unscheduled flight movement data not being available. As simplifications due to the unavailability of data, engine degradation is disregarded, and a single specific engine model is assigned for each engine family for aircraft type instead of considering all engine options.

For each of the four aircraft classes, projections are calculated in yearly steps up to 2040 by considering that new passenger and cargo traffic demand for each world region, according to ICAO’s long-term passenger and cargo traffic forecasts from 2021^72^, is met by a representative aircraft. These newly added aircraft also cover the traffic demand associated with the retirement expected for each aircraft type given an initial age distribution, and class-specific retirement rates from ref. ^73^, which do not vary across our three scenarios. Due to limitations of data availability, no regional differences are considered for initial age distributions of each aircraft type or for retirement rate curves of each aircraft class.

These generic representative aircraft start from the performance of (real) reference aircraft types in 2018 and each year the performance of newly introduced aircraft is progressively increased based on the engine fuel burn and NO_*x*_ emission goals for new aircraft identified by ICAO’s 2017 independent expert integrated review panel^71^ and those used in ICAO’s environmental trends analysis in 2019^74^. The emission goals defined by the expert panel relate to engines which enter into service by the target dates of 2027 and 2037. In this study, the use of conventional (fossil-based) jet fuel is assumed, and future engine performance is modeled with a continuous improvement, with piecewise linear interpolation between those goal points, extrapolating improvements from 2037 to 2040. From this, we adopt annual fuel burn reductions of new aircraft through technology of 1.16% (low emissions), 0.96% (baseline), and 0.57% (high emissions). Transformative engine or aircraft technologies, such as electric or hydrogen propulsion, beyond those considered relevant until 2037 by the ICAO panel, are not contemplated in the analysis. Fuel consumption per traffic level is reduced each year considering operational improvement targets by ICAO^75^, which amounts to 9.0% between 2010 and 2040 in the baseline and low emissions scenarios and 5.8% in the high emissions scenario. Both fuel burn and emission indices of each pollutant is modeled using values specific to each aircraft type and region. Improvements in NO_*x*_ emissions are modeled such that in all cases aircraft reach the 2027 goal in that year, with a further reduction of 10% in emission indices by 2037 applied in the low emissions scenario. A continuous increase in overall engine pressure ratio is adopted in the baseline projection until the year 2027 to values of 70 (widebodies), 65 (narrowbodies), and 60 (regional jets). For the low emissions scenario, pressure ratios reach those values in 2037 instead. For the high emissions scenario, pressure ratios are the same as the baseline case until 2027, but then increase up to 90 (widebodies), 80 (narrowbodies), and 70 (regional) in the year 2037.

Future aircraft emissions of CO, hydrocarbons, and nonvolatile particulate matter are estimated by multiplying projected fuel burn with the emission indices found for 2018, using values specific to each region and aircraft type. Simulations including only NO_*x*_ emissions from aircraft provide an indication of the limit on the impact of a potential overestimation of non-NO_*x*_ emissions due to this assumption of constant EI.

### Atmospheric modeling

The atmosphere is modeled with the GEOS-Chem Classic v13.3 model^76^, driven by MERRA-2 reanalysis meteorological fields for the years 2005 and 2019^77^ and driven by meteorological fields for the year 2040 from GCAP 2.0^46^, which are generated for CMIP6 (Coupled Model Intercomparison Project, phase 6) scenarios^78^ using the global climate model E2.1 from the NASA Goddard Institute for Space Studies (GISS)^47,48^. GEOS-Chem simulates tropospheric and stratospheric photochemistry as described by Eastham et al.^79^. Sulfate-nitrate-ammonium aerosol thermodynamics modeling is described by Park et al.^80^ and uses the ISORROPIA thermodynamic module^81^. Non-aviation anthropogenic emissions for the years 2005 and 2019 are from the CEDS v2 inventory^82^. Emissions and surface boundary conditions for each 2040 scenario are from CMIP6^42^, processed by GCAP 2.0 as described in ref. ^46^. In addition to ammonium, sulfate, and nitrate, the model’s PM_2.5_ definition^76^ also includes hydroxymethanesulfonate (HMS), hydrophilic and hydrophobic black carbon, hydrophilic and hydrophobic organic carbon aerosol, simplified secondary organic aerosol, accumulation mode sea salt aerosol, and mineral dust with reference effective radii of 0.7 and 1.4 μm. Modeled PM_2.5_ concentrations are corrected for standard temperature and pressure conditions, as is customary in air quality data used in epidemiological studies. A comparison of the 2019 GEOS-Chem simulation performed here against observations of air quality monitoring stations and ozonesondes is presented in Supplementary Note 2. GEOS-Chem underestimates background ozone mixing ratios relative to ozonesondes by seasonal averages of between 3% and 28%. Modeled concentrations show lower variability than ground measurement stations, consistent with large grid box sizes of a global model. Over Europe and North America, the model has median biases of −8% to +9% for ozone, 0% to +28% for PM_2.5_, and −62% to −44% for NO_2_ (Supplementary Fig. 9). Over Asia, median biases are −2% to +147% for ozone, +3% to +40% for PM_2.5_, and −65% to −44% for NO_2_ (Supplementary Fig. 10). These comparisons of background concentrations are not a direct assessment of performance in quantifying aviation’s impacts, which have marginal effects on that background, but are still affected by possible biases in the model’s representation of the atmosphere. Model output from the GEOS-Chem runs underlying this study is also part of a separate model intercomparison concerning specifically the atmospheric impacts of aircraft emissions^83^.

Simulations are performed at 2° latitude and 2.5° longitude resolution globally, with either 72 (MERRA-2 meteorology) or 40 (GCAP 2.0 meteorology) vertical levels from the surface up to 0.01 hPa. We note that results obtained with GCAP 2.0 and those obtained with MERRA-2 fields are not to be directly compared, as each set of fields leads to significantly different vertical concentration profiles of both background and aircraft attributable species (as shown in Supplementary Fig. 29). Results for the three scenarios quantifying impacts of all aircraft emissions in 2019—full-flight, LTO-only, and no aviation emissions—are further refined by nested simulations using 0.5° × 0.625° grids over Asia (60°E–150°E, 11°S–55°N), Europe (30°W–50°E, 30°N–70°N), and North America (140°W–40°W, 10°N–70°N). The nested simulations use the global coarse resolution runs for boundary conditions. In the results section on future emissions scenarios, these higher resolution refinements for 2019 are excluded from analysis in order to match the settings of the simulations of future scenarios. At all spatial resolutions, the timestep for transport and convection is 10 min and for chemistry and emissions it is 20 min.

Simulations with only aircraft emissions of specific classes of aircraft or from flights of specific lengths are performed, without nested domains, to estimate the share of health impacts attributed to each subset of emissions. Therefore, Fig. 2 presents mortality counts estimated with the higher resolution nested simulations, but percentages attributed to each aircraft classes from simulations without them. Since aircraft impacts on ground-level NO_2_ are more sensitive to model resolution, their breakdown per aircraft class is omitted in Fig. 2.

### Human health impacts

Exposure to pollutants is calculated using population maps for 2005 and 2019 from LandScan, totaling 6.44 and 7.56 billion people, respectively^84,85^. Population distributions for the year 2040 from Jones and O’Neil are used^22^, which take into account different national population and urbanization projections consistent for each SSP^23^. Global population count in billions is 8.35 (SSP1), 8.81 (SSP2), 9.28 (SSP3), 8.76 (SSP4), and 8.41 (SSP5). Health impacts are quantified as the increase in yearly mortality of persons older than 30 years due to long-term exposure to PM_2.5_, ozone, and NO_2_, using non-age-specific CRFs for the entire adult population. The fraction of the population that is aged 30 or above is taken as country-specific values according to the Global Health Estimates (GHE) from the World Health Organization for the years 2005 (46.0% globally) and 2019 (50.1%)^86,87^; the country-specific fractions for the year 2040 are given by the SSP definitions^23^, with global averages ranging between 48.6% (SSP3) and 50.1% (SSP5). Cause-specific baseline mortality rates are also taken from the GHE for 2005 and 2019, with values for age brackets of 30–49, 50–59, 60–69, and 70+ years being combined for each country into an average of the whole population of 30+ years using the fraction of the country’s population in each bracket at the given year. Mortality rates for 2040 specific for each cause, country, age, and SSP are taken from the International Futures (IFs) model v8.28^88^, and are likewise combined using SSP-specific future age distributions^23,42^. Health impacts for 2040 estimated using baseline mortality rates from 2019 are shown in Supplementary Table 13, and estimates using rates for 2040 from a different, more recent, projection that is not SSP-specific are given in Supplementary Table 14. Exposure and health impact are calculated with a global 0.5° × 0.625° grid (edge-aligned with the atmospheric model’s 2° × 2.5°) in which a country is assigned to each cell based on geocoding data from OpenStreetMaps, accessed via the Nominatim search tool.

Mortality due to noncommunicable diseases and lower respiratory infections associated with annual average exposure to PM_2.5_ is quantified using the GEMM nonlinear CRF, which was derived from a meta-analysis including a wide range of concentration values^89^. COPD mortality associated with peak-season average daily maximum 8-hour average (MDA8) ozone is quantified using the relative risk of 1.074 per 10 ppbv, as done by Brauer et al.^90^ for the Global Burden of Disease (GBD) 2021. The 2005 and 2019 COPD baseline mortality rates consist of GHE codes 1180 and 1190 (COPD and asthma). For 2040, COPD mortality rates are taken as the non-communicable respiratory diseases corrected by the country, sex, and age-specific fraction of those that fall under COPD in GHE 2019 (i.e., rates of codes 1180 + 1190 divided by rate of code 1170). Peak-season ozone is taken as the grid-cell local highest 6-month rolling average MDA8 concentration, looping over December and January. All-cause nonaccidental mortality associated with annual average NO_2_ is quantified using the relative risk of 1.05 per 10 μg m^−3^, based on a meta-analysis from ref. ^55^. The same constant factors of 1.996 (ozone) and 1.914 (NO_2_) used by ref. ^53^ are adopted to convert mixing ratios in ppb to mass concentrations in μg m^−3^. The CRFs adopted here for ozone and NO_2_ have no counterfactual concentration, and the CRF for PM_2.5_ has a value (2.4 µg m^−3^) that is lower than the background concentration for 99.92% of the population (in the 2019 case), such that almost all of aircraft-attributable increases in pollutant concentrations contribute to the health impact estimates.

Uncertainty in mortality estimates is calculated considering only the uncertainty associated with the CRFs. For PM_2.5_, a normal distribution of the theta parameter in the GEMM model is considered, using the standard deviation provided with the CRF^89^. For ozone and NO_2_, triangular distributions are used for the hazard ratios, defined by the central values and the 95% confidence intervals reported with the CRFs^55,90^. The uncertainties are calculated using a Monte Carlo approach with 10^4^ samples, with 2.5th and 97.5th percentiles reported along with the results from nominal input values.

## Supplementary information

Transparent Peer Review file

Supplemental Material

## Data Availability

The processed data directly used in the analyses, figures, and tables of the article are available in the following public repository: https://doi.org/10.4121/79395594-9c12-451a-9f59-081db67605e0. This dataset includes netCDF files with the aircraft emissions used as input to the atmospheric simulations, atmospheric model outputs used for impact quantification, and the baseline mortality rates considered.
