# Supplementary material for: Global air quality and human health impacts of growing aircraft emissions
Source: Commun Earth Environ. 2026 Jun 16;7(1):806. doi: 10.1038/s43247-026-03732-4 (PMC13630550; doi:10.1038/s43247-026-03732-4)
Supplement: Supplementary file 1 [file 43247_2026_3732_MOESM1_ESM.pdf]

nature portfolio

Peer Review File

Global air quality and human health impacts of growing aircraft emissions

Corresponding Author: Dr Irene Dedoussi

This manuscript has been previously reviewed at another journal. This document only contains information relating to versions considered at Communications Earth & Environment.

This file contains all editorial decision letters in order by version, followed by all author rebuttals in order by version.

Version 0:

Decision Letter:

** Please ensure you delete the link to your author home page in this e-mail if you wish to forward it to your coauthors **

Dear Dr Dedoussi,

Thank you for submitting your manuscript titled "Global air quality and human health impacts of growing aircraft emissions" to Communications Earth and Environment.

We note that the manuscript was not yet revised based upon the most recent round of reviewer reports at Nature Communications. The reviewers raised earlier substantial concerns that must be addressed. In light of these comments, extensive revisions will be required before we can further consider the manuscript for publication. We would, however, be interested in considering a revised version that fully addresses these concerns.

Moving forward, we plan to seek independent advice from an adjudicating referee.
But before we do so, please revise your manuscript in response to the remaining concerns and supply us with a point-by-point response to the referees' reports. We will send your responses, the reports and the revised manuscript to the adjudicator.
We would also appreciate suggestions for adjudicators that you have not collaborated with over the past 5 years or so, and who you would trust to be fair as well as competent to evaluate your manuscript. We cannot promise to use any of them, but we will do so if it seems possible and appropriate.

When resubmitting, please provide a point-by-point response to the reviewers' comments. Please submit your responses as a separate file, distinct from your cover letter where you can add responses to the Editors' comments that you do not want to be made available to the reviewers. Word files are preferred. We recommend that any figures, tables or graphs that are included in the response to reviewers are also included in the main article or Supplementary Information.

If the revision process takes significantly longer than three months, we will be happy to reconsider your paper at a later date, as long as nothing similar has been accepted for publication at Communications Earth & Environment or published elsewhere in the meantime.

We are committed to providing a fair and constructive peer-review process. Please do not hesitate to contact us if you wish to discuss the revision in more detail.

Please use the following link to submit your revised manuscript, point-by-point response to the reviewers' comments with a list of your changes to the manuscript text (which should be in a separate document to any cover letter), a tracked-changes version of the manuscript (as a PDF file) and any completed checklist:

Link Redacted

** This url links to your confidential home page and associated information about manuscripts you may have submitted or be reviewing for us. If you wish to forward this email to co-authors, please delete the link to your homepage first **

Please do not hesitate to contact us if you have any questions or would like to discuss the required revisions further. Thank you for the opportunity to review your work.

Best regards,

Alice Drinkwater, PhD
Associate Editor
Communications Earth & Environment
Consulting Editor
Communications Sustainability

EDITORIAL POLICIES AND FORMAT

If you decide to resubmit your paper, please ensure that your manuscript complies with our editorial policies and complete and upload the checklist below as a Related Manuscript file type with the revised article:

For Manuscripts that fall into the following fields:
• Behavioural and social science
• Ecological, evolutionary & environmental sciences
• Life sciences
An updated and completed version of our Reporting Summary must be uploaded with the revised manuscript
You can download the form here:
https://www.nature.com/documents/nr-reporting-summary.pdf

For your information, you can find some guidance regarding format requirements summarized on the following checklist:
(https://www.nature.com/documents/commsj-phys-style-formatting-checklist-article.pdf) and formatting guide
(https://www.nature.com/documents/commsj-phys-style-formatting-guide-accept.pdf).

** Visit Nature Portfolio's author and referees' website at www.nature.com/authors for information about policies, services and author benefits**

Communications Earth & Environment is committed to improving transparency in authorship. As part of our efforts in this direction, we are now requesting that all authors identified as 'corresponding author' create and link their Open Researcher and Contributor Identifier (ORCID) with their account on the Manuscript Tracking System prior to acceptance. ORCID helps the scientific community achieve unambiguous attribution of all scholarly contributions. You can create and link your ORCID from the home page of the Manuscript Tracking System by clicking on 'Modify my Springer Nature account' and following the instructions in the link below. Please also inform all co-authors that they can add their ORCIDs to their accounts and that they must do so prior to acceptance.
https://www.springernature.com/gp/researchers/orcid/orcid-for-nature-research

For more information please visit http://www.springernature.com/orcid

If you experience problems in linking your ORCID, please contact the Platform Support Helpdesk.

Version 1:

Decision Letter:

** Please ensure you delete the link to your author home page in this e-mail if you wish to forward it to your coauthors **

Dear Dr Dedoussi,

Your manuscript titled "Global air quality and human health impacts of growing aircraft emissions" has now been seen by our reviewers, whose comments appear below. In light of their advice we are delighted to say that we are happy, in principle, to publish a suitably revised version in Communications Earth & Environment, provided remaining reviewer concerns are met.

We therefore invite you to revise your paper one last time to address the remaining concerns of our reviewers, which

pertains to additional justification of the Concentration-Response functon(CRF) approach for calculating additional deaths, and a thorough discussion of related uncertainties. At the same time we ask that you edit your manuscript to comply with our format requirements and to maximise the accessibility and therefore the impact of your work.

EDITORIAL REQUESTS:

Please review our specific editorial comments and requests regarding your manuscript in the attached "Editorial Requests Table".

*****Please take care to match our formatting and policy requirements. We will check revised manuscript and return manuscripts that do not comply. Such requests will lead to delays. *****

Please outline your response to each request in the right hand column. Please upload the completed table with your manuscript files as a Related Manuscript file.

If you have any questions or concerns about any of our requests, please do not hesitate to contact me.

SUBMISSION INFORMATION:

In order to accept your paper, we require the files listed at the end of the Editorial Requests Table; the list of required files is also available at https://www.nature.com/documents/commsj-file-checklist.pdf .

OPEN ACCESS:

Communications Earth & Environment is a fully open access journal. Articles are made freely accessible on publication. For further information about article processing charges, open access funding, and advice and support from Nature Portfolio, please visit https://www.nature.com/commsenv/open-access

At acceptance, you will be provided with instructions for completing the open access licence agreement on behalf of all authors. This grants us the necessary permissions to publish your paper. Additionally, you will be asked to declare that all required third party permissions have been obtained, and to provide billing information in order to pay the article-processing charge (APC).

Please use the following link to submit the above items:
Link Redacted
** This url links to your confidential home page and associated information about manuscripts you may have submitted or be reviewing for us. If you wish to forward this email to co-authors, please delete the link to your homepage first **

We hope to hear from you within two weeks; please let us know if you need more time.

Best regards,

Alice Drinkwater, PhD
Associate Editor
Communications Earth & Environment
Consulting Editor
Communications Sustainability

REVIEWERS' COMMENTS:

Reviewer #1 (Remarks to the Author):

Note that reference line numbers are according to the revised manuscript file.
Lines 56 to 66 describing other studies is hard to read and could be simplified here. The question that springs to mind when reading this section is why such different results from the Eastham et al (2024) study which also uses the GEOS-Chem model and 2018 aircraft data and would be expected to be comparable. One must read on to page 9 (lines 154 to 156) to find the explanation lies in the different CRF for O3 used - illustrating the impact and uncertainty of the particular CRFs used.
Line 67 refers to Ref 38 which is a study that also considered aviation's emissions and air quality impacts out to 2050. Interestingly, this study by Phoenix et al (2019) a very different interpretation to the modelled perturbations was made. They did not apply CRF approach to estimate the health impacts through mortality, they instead assessed the aviation impact on the frequency of extreme air quality events (the frequency of exceedances of the EPA NAAQS standard due to aviation emissions for O3 and PM2.5). They estimated surface O3 and PM2.5 aviation-induced perturbations at similar levels (although the PM2.5 was not so spatially dispersed as in this study). Overall, they concluded that "aviation has a very small impact on increasing the frequency of O3 extreme air quality events and has essentially no impact on the frequency of

PM2.5 extreme air quality events”. I find it hard to understand these very different interpretations as you would think that there should be some correlation with extreme air quality events and mortality. I think it would be useful for the authors to explain how the relatively small perturbations from aviation emissions could impact on air quality events as well as the straight application of a CRF. The CRF approach does seem to suggest that very low additional concentrations can be equally damaging irrespective of the total absolute concentrations including backgrounds?
Lines 94 to 98: this could be condensed and the various % values are hard to read.
Lines 98 to 105: “Averaging within only populated areas of the Northern Hemisphere, full-flight emissions increase the PM2.5 concentration by 0.051 µg·m–3.” The values here are different to the World values for population weighted concentrations given further down in Table 1 (0.121 ug/m3 of PM2.5) what am I missing? It seems a bit confusing.
Fig 1 is very useful and informative. It is clear from the figure that the largest contributions from aviation to PM2.5 coincide with very populated regions.
Lines 137 to 146: the role of non-Nox emissions in the PM2.5 estimates in interesting and particularly the role of S. The study looks at how different the estimates are if non-Nox emissions are not included but how significant are the non-S/non-Nox emissions? This is particularly interesting in the case of future very low or zero S fuels including some SAFs.
Lines 179 to 184 and the figure 2 do not add much to the understanding without a better explanation of how much of the emissions of Nox come from these categories etc.
I found Figure 4 hard to understand. On the RHS as the average population exposure changes for 2019 didn’t match up with the data in table 1. Should they? I liked the sensitivity approach though and the way that the different future scenarios impact the results.
Lines 260 to 294 Actual aviation emissions estimates used for each of the years (2005, 2019, and 2040 base/high/low) in a table would have helped understand this section. Even in the methods section there is no aviation emission estimate data.
Lines 329 to 426 Discussion section
The authors duly note that “Uncertainty in the CRF propagates to the health impacts of aviation, so care must be taken when considering the absolute magnitude of impacts”. And I agree that the relative evolution of the impacts over time in this study would not be affected by CRF. However, the statement that “the absolute numbers presented reflect the atmospheric changes that could be expected if all aircraft emissions were to be eliminated” is only true based on this particular modelling study with all the caveats applied.
Lines 394 to 401 Here reference is made to ultra-low S fuel and this would be a very useful link to the significance of Sox emissions in the PM2.5 aerosol predictions made earlier.
Lines 401 to 410 This is a very important point regarding the relative toxicity of BC. The local air quality impacts of PM2.5 are based on mass, the contribution of nvPM or BC to mass concentrations is likely to be very low due to their very small size (aviation nvPM are less that 30nm). The toxicity of the various components of PM2.5 are not generally considered in these large epidemiological studies. The measurement of UFP is at an early stage compared to the ambient measurement of mass concentrations and as knowledge progresses the nature of the PM may become more relevant rather than purely the mass concentration.
Methods section on aircraft emissions
This would really benefit from a table of emissions (either here or in the results section) rather than just a reference to another paper (I had to look it up). There is rather a lot of information that could be transferred to the SI too to improve readability. The input data on aviation emissions are pretty critical to the understanding of the paper. The data would also benefit from some contextual comparison to assess how the data compare with other studies.

** Visit Nature Portfolio's author and referees' website at www.nature.com/authors for information about policies, services and author benefits**

Open Access This Peer Review File is licensed under a Creative Commons Attribution 4.0 International License, which permits use, sharing, adaptation, distribution and reproduction in any medium or format, as long as you give appropriate credit to the original author(s) and the source, provide a link to the Creative Commons license, and indicate if changes were made.
In cases where reviewers are anonymous, credit should be given to 'Anonymous Referee' and the source.
The images or other third party material in this Peer Review File are included in the article's Creative Commons license, unless indicated otherwise in a credit line to the material. If material is not included in the article's Creative Commons license and your intended use is not permitted by statutory regulation or exceeds the permitted use, you will need to obtain permission directly from the copyright holder.
To view a copy of this license, visit https://creativecommons.org/licenses/by/4.0/

Dear editor, reviewers,

We thank the reviewers for taking the time to provide constructive feedback to our manuscript. We have carefully considered and addressed the reviewers' feedback and believe that this has considerably improved the quality of our work. In this document you will find our response to the reviewers' comments, answering their questions and specifying how their feedback is integrated into the revised manuscript. Reviewer comments are shown in *italics*, and our response is in **bold**. Line, figure and table numbers refer to the updated manuscript with tracked changes.

Overall, we have recalculated all the health impact estimates to ensure we are applying to most up-to-date and appropriate human health response functions, as encouraged by Reviewer #1. As we mention in the Discussion in the manuscript, care must be taken when interpreting the magnitudes of quantified health impacts (and the relative importance of impacts of one pollutant over another) as they are highly dependent on the choice of CRF. This is also explored in Supplementary Tables 9–11, where we present results calculated with 40 different CRFs. The updated choice of "baseline" CRFs reduced quantifications of mortality in 2019 due to ozone by 69% and increase those due to NO_2 by 146%. We have also revised the presentation of the human health estimates to avoid any misinterpretation that the impacts of different species can be summed. Furthermore, we have clarified aspects of our modelling of future aviation emissions and reaffirmed GEOS-Chem's suitability for this work.

Thank you again for considering our submission to *Communications Earth & Environment*, we look forward to hearing from you in due course.

Sincerely,

Irene Dedoussi, on behalf of all authors

Reviewer #1 (Remarks to the Author):

I appreciate that the authors have addressed some of my previous comments. However, several questions regarding future emissions inventories and health impacts remain unresolved. The authors could consider to address these comments by modifying modeling parameters and statements, or add useful discussions.

1. I appreciate the authors' explanation of the scenario design. However, I am still concerned about modeling fuel burn and NOx emissions separately, particularly in future projections. Both depend strongly on engine technologies, and the combination of high fuel efficiency with low OPR creates a technical inconsistency. While the authors describe this as an extreme case, in practice fuel efficiency gains are closely linked to higher OPR, which has historically been a primary pathway for improving engine performance. Although airframe and operational changes can also contribute, in scenarios with the largest efficiency gains it is unrealistic to assume that high-OPR engines would not play a central role. Thus, combining high fuel efficiency with low OPR does not align with technological reality and introduces inconsistency into the modeling framework.

We thank the reviewer for raising this important point regarding the historical correlation between fuel efficiency and OPR. While we agree that past fuel efficiency gains have largely been driven by higher OPR (and consequently higher NO_x), given the large uncertainties in predicting technological advancement over decades, we opted for parameter combinations that cover a wide range of future NO_x emission totals. Because alternative technological pathways exist to improve efficiency without relying solely on OPR increases, we want the range of our results to reflect these possibilities, such as aircraft aerodynamic efficiency improvements, airframe structure mass reductions, reduced fan pressure ratios, and improved integration of propulsion system with the airframe. Furthermore, our projection aligns with the approach taken by the ICAO independent expert review [1], which likewise evaluates these parameters separately (fuel efficiency through technology, fuel efficiency through operation, and NO_x emission "intensity" through technology). We have added text on lines 484–487 clarifying our rationale and its alignment with the ICAO approach.

2. I have some doubts about the assumptions used to project future aircraft emission. First, the study assumes the same rate of fuel efficiency improvement across all aircraft with the distinctions between different air craft types. Countries and regions have very different fleet compositions. For example, wide-body aircrafts are the majority in the UAE and regional jets are common in the United States. This uniform assumption reduces accuracy. Second, the study assumes constant fleet composition over time. This overlooks current market shifts where wide-body demand is declining while narrow-body aircraft are expanding. Because these categories differ in both fuel use and NOx emissions, ignoring this trend can bias the results. Third, the analysis does not consider regional differences in aircraft age and fleet renewal. Average fleet age varies widely, such as 9.7 versus 16.5 years according to IATA, and regions adopt new technologies at different paces. Treating them as uniform misrepresents the timing of emissions reductions.

Overall, the method assumes identical emissions factor trends across all regions, with traffic growth as the only country-specific parameter. This oversimplifies aviation systems and may lead to significant errors. For instance, developed countries may have slower growth but will likely reduce emissions faster due to active fleet renewal. In contrast, many emerging countries are growing quickly but already have newer fleets that may not improve much in the short term. These countries often have higher background pollution and denser populations, so any underestimation of aircraft emissions could lead to underestimating the actual population exposure. These factors can strongly influence the results and should be better reflected in the model.

We appreciate the reviewer detailing these nuances regarding the future fleet modeling assumptions. We address the three primary concerns below:

Fuel efficiency: Our assumption of "uniform" fuel efficiency improvement aligns with the long-term trends analysis by ICAO (Fleming and de Lépinay [2]), which adopts the same value for all aircraft classes. While we considered one of the alternative options for the fuel burn technology parameter (k_{tec}), based on Cumpsty et al. [1], which consists of different values for each aircraft class (see Supplementary Fig. 3 and Supplementary Table 3), we adopted the estimates from Fleming and de Lépinay as they considered multiple levels of fuel efficiency, more closely matching our methodology.

Fleet composition: Differences in fleet compositions in terms of aircraft classes are captured by our initial list of flights. These are kept constant for simplicity, and as we did

not want to impart judgement on our estimates with regard to future changes in the relative class makeup of aircraft fleets. There is indeed a recent trend towards more narrowbodies in favor of widebodies, as noted on lines 41–42 in the Supplementary Note 1. But whether such a trend can be expected over the timeline of our projection requires deliberations about the global market and inter-regional relations that are outside the scope of this study.

Aircraft retirement rates: As described in the Supplementary Note 1, a different (initial) age distribution is considered for each aircraft type. Due to a lack of more granular data, differences across regions in age distribution of the same aircraft type are not considered. Inter-regional differences in fleet compositions, however, capture some of that effect; i.e. some regions have more/less newer/older aircraft types at the start of the projection, and that effect propagates through each annual step. This, combined with region-specific traffic growth, means that the example given by the reviewer is indeed captured by our projection: newer fleets, reflected by newer aircraft types and more aircraft introduced to meet traffic growth, have lower retirement rates. Lastly, we also note that our sensitivity study showed that even a significant change of globally reducing or delaying median retirement ages by 10 years (Supplementary Fig. 1) results in a smaller change in NO_x emissions over the period 2027–2040 (−4.5%, +3.4%) compared to changing traffic growth scenario (low: −17.0%, high: +7.9%), as shown in Supplementary Table 3.

We have added a sentence on the main text (lines 496–498), further highlighting the simplifications adopted in our modeling.

3. I appreciate that the authors have revised the results by removing NO2 from the main results. However, I remain concerned that NO2-related health impacts are still included. The causal relationship between NO2 and mortality remains highly uncertain, and the CRFs cited (https://doi.org/10.1016/j.envint.2020.105998) were not intended for direct quantification. The authors of that study clearly state: "This review of associations between NO2 and O3 and mortality in epidemiological cohort studies provides evidence for the assessment of the strength of evidence for associations only. It has focused on results form single pollutant models. The question of the independence of these associations from other pollutants requires careful consideration. A separate causal determination is required to proceed to quantification of health impacts." Given this statement, I do not think the CRFs in that paper are suitable for quantifying the independent effects of NO2 and O3, especially without controlling for PM2.5.

We thank the reviewer for this feedback. While there is still uncertainty about the magnitude of impacts of pollutants on mortality and the interdependency of those effects, all three pollutants analyzed here are both of concern to human health and significantly impacted by aircraft emissions. Because of that, we present health impact quantifications for the three pollutants when discussing the outcomes of the various scenarios evaluated.

However, the reviewer has raised a valid point. To address it, we have redone all health impact quantifications using an ozone CRF that has been used before in the Global Burden of Disease 2021 with the specific purpose of quantifying mortality impacts independently from $PM_{2.5}$ (Brauer et al. [3]). Likewise, NO_2 impacts are recalculated using a CRF from Kasdagli et al. [4], who conducted an analysis as an update from Huangfu and Atkinson [5]. Although this NO_2 CRF is still based on single-pollutant models, given the availability of evidence for the meta-analysis, Kasdagli et al. also investigated studies that adjusted NO_2 impacts for other pollutants and found that "In most of the studies, further adjustment for other pollutants did not affect the effect estimate of interest." Additionally, we have

changed the presentation of mortality quantifications due to each pollutant such that they are always separate, to not imply an assumption that $PM_{2.5}$ and ozone impacts are completely independent and could thus be summed together. These changes are seen in Fig. 2, Table 2, and throughout the text. The dependance of health impacts (primarily in terms of their magnitude) on the choice of CRF is noted on lines 150–160, 167–171, 336–340, 348–351, 357–361, 366–369, 404–406. The effect of changing CRFs on our health impact quantifications is shown in Supplementary Tables 9–11, which have been updated and expanded to include 15 additional CRFs and 3 additional references.

Reviewer #2 / #3 (Remarks to the Author):

I thank the authors for revising the manuscript and addressing previous round of comments. The only remaining concern I have with is the new text below, and the associated table.

Supplemental Lines 273 – 277: I don't agree with this statement. "Therefore, lower or higher modeled background concentrations do not necessarily point to lower or higher sensitivity of concentrations to (marginal) emissions from aircraft." If GEOS-Chem underpredicts background concentrations, the corresponding sensitivities to marginal emissions are also underestimated. I suggest that the authors reword this, and provide a cautionary statement on the implication of these underestimates in the main manuscript.

To address this concern and improve clarity, we have rewritten the lines in question in the SI and added further information in the main text (lines 563–572) about the comparisons made against ozonesondes and ground-level air quality monitoring stations and their implications.

Supplemental Table 4: This comparison against ozone sondes points to a systematic underestimate and potential weakness in GEOSChem to study emissions sources aloft, such as aircraft emissions, which is the focus of this study. Can the authors provide additional justification for the choice of this model, given this demonstrated underestimate of vertical transport? And is this underestimate related to the findings that non-LTO emissions reduce ground-level NO2?

We appreciate the reviewer highlighting this point. The GEOS-Chem model has been used in previous studies evaluating air quality impacts of aircraft emissions [6-8]. Model intercomparisons have shown that GEOS-Chem's performance in replicating measured vertical profiles of ozone, as we briefly evaluated in Supplementary Note 2, is comparable to that of alternative, widely used, chemistry transport models [9-11]. GEOS-Chem has also been shown to yield comparable results to other models specifically when evaluating impacts of high altitude aircraft emissions [12]. Based on this, we consider GEOS-Chem one of the most appropriate and state-of-the-science tools available for the simulations needed for our study.

We do not have a definitive hypothesis about a potential link between the finding that non-LTO emissions reduce ground-level NO_2 and the modeled bias towards low background ozone in the free troposphere. Determining whether these two effects are related and identifying the mechanisms involved would require a dedicated study, ideally featuring multiple atmospheric models. We agree this is a compelling question for the community, but a thorough investigation of this specific mechanism falls outside the scope of the current work.

[1] Cumptsy et al. 2019, https://store.icao.int/en/independent-expert-integrated-technology-goals-assessment-and-review-for-engines-and-aircraft-english-printed

[2] Fleming and de Lépinay 2019, https://www.icao.int/sites/default/files/sp-files/environmental-protection/Documents/EnvironmentalReports/2019/ENVReport2019_pg17-23.pdf

[3] Brauer et al. 2024, https://doi.org/10.1016/S0140-6736(24)00933-4

[4] Kasdagli et al. 2024, https://doi.org/10.3389/ijph.2024.1607676

[5] Huangfu and Atkinson, https://doi.org/10.1016/j.envint.2020.105998

[6] Barrett et al. 2010, https://doi.org/10.1021/es101325r

[7] Eastham et al. 2024, https://doi.org/10.5194/acp-24-2687-2024

[8] Quadros et al. 2020, https://doi.org/10.1088/1748-9326/abb2c5

[9] Cohen et al. 2025, https://doi.org/10.5194/acp-25-5793-2025

[10] Fritz et al. 2022, https://doi.org/10.5194/gmd-15-8669-2022

[11] Lin et al. 2024, https://doi.org/10.5194/acp-24-8607-2024

[12] van ’t Hoff et al. 2025, https://doi.org/10.5194/acp-25-2515-2025

Dear editor, reviewers,

We thank the reviewer for taking the time to carefully read and provide constructive feedback to our manuscript. We have considered and addressed the reviewer's feedback and believe that this has considerably improved the quality of our work. In this document you will find our response to the reviewer's comments, answering their questions and specifying how their feedback is integrated into the revised manuscript. Reviewer comments are shown in *italics*, and our response is in **bold**. Line, figure and table numbers refer to the updated manuscript with tracked changes.

Thank you again for considering our submission to *Communications Earth & Environment*, we look forward to hearing from you in due course.

Sincerely,

Irene Dedoussi, on behalf of all authors

Reviewer #1 (Remarks to the Author):

Note that reference line numbers are according to the revised manuscript file.

Lines 56 to 66 describing other studies is hard to read and could be simplified here. The question that springs to mind when reading this section is why such different results from the Eastham et al (2024) study which also uses the GEOS-Chem model and 2018 aircraft data and would be expected to be comparable. One must read on to page 9 (lines 154 to 156) to find the explanation lies in the different CRF for O3 used - illustrating the impact and uncertainty of the particular CRFs used.

We have simplified the text in question and added a sentence (lines 57–68) to note that the wide range of magnitudes is due to the factors listed earlier in the paragraph, such as choice of CRF. As the reviewer mentions, we include a deeper discussion of these effects once our results are shown in the Results section (lines 155–174).

Line 67 refers to Ref 38 which is a study that also considered aviation's emissions and air quality impacts out to 2050. Interestingly, this study by Phoenix et al (2019) a very different interpretation to the modelled perturbations was made. They did not apply CRF approach to estimate the health impacts through mortality, they instead assessed the aviation impact on the frequency of extreme air quality events (the frequency of exceedances of the EPA NAAQS standard due to aviation emissions for O3 and PM2.5). They estimated surface O3 and PM2.5 aviation-induced perturbations at similar levels (although the PM2.5 was not so spatially dispersed as in this study). Overall, they concluded that "aviation has a very small impact on increasing the frequency of O3 extreme air quality events and has essentially no impact on the frequency of PM2.5 extreme air quality events". I find it hard to understand these very different interpretations as you would think that there should be some correlation with extreme air quality events and mortality. I think it would be useful for the authors to explain how the relatively small perturbations from aviation emissions could impact on air quality events as well as the straight application of a CRF. The CRF approach does seem to suggest that very low additional concentrations can be equally damaging irrespective of the total absolute concentrations including backgrounds?

Thank you for raising this point, as it highlights the different ways of quantifying air quality impacts. The differences between our study and that of Phoenix el al. (2019) are expected due to the different concepts behind each type of health impact metric. Extreme air quality events occur due to localized acute increases in pollutant concentration. Our metric of health impact is the expected increase in mortality rates due to (even slight) increases in long-term pollution exposure. The health impact quantification method used here is a reflection of epidemiological evidence suggesting that slightly higher long-term exposure to pollutants is associated with slightly worse health outcomes. There is also growing evidence that there is no minimum level of exposure below which there is no damage (counterfactual concentration of zero), although there is less certainty or consensus over this last assertion.

Non-LTO emissions, owing to their altitude and spatial distribution, have a particularly widespread impact on ground-level concentrations (as shown both here and by Phoenix et al.). Additionally, the method of using a global simulation with grid cells measuring hundreds of kilometers per side (Phoenix et al. resolution is 2° x 2.5°) and with aircraft emissions modeled as (small) constant emission rates applied uniformly over each cell, while adequate to estimate broad impacts at global or regional scale, is not able to resolve local air quality conditions or localized impacts from aviation.

The CRFs we use for ozone and NO_2 do not have a counterfactual concentration, while the CRF for $PM_{2.5}$ has a counterfactual value of 2.4 μg/m^3, lower than the annual average background concentration for 99.92% of the population (as modeled for 2019). This means that all the aircraft-attributable small increases in pollutant concentrations over background values worldwide count towards our mortality metrics. We have added this observation as a sentence in the Methods (lines 648–651). Conversely, small increases over background only count towards the number of extreme air quality events if the background was already marginally close to the threshold for such events – i.e., most of non-LTO impacts do not affect this metric. Therefore, the conclusions of Phoenix et al. are consistent with the results in both studies.

Lines 94 to 98: this could be condensed and the various % values are hard to read.

Since both area-weighted and population-weighted percentages convey the message that impacts are concentrated in the Northern Hemisphere, we have simplified the sentence by keeping only the population-weighted values (lines 98–103).

Lines 98 to 105: "Averaging within only populated areas of the Northern Hemisphere, full-flight emissions increase the PM2.5 concentration by 0.051 μg·m−3." The values here are different to the World values for population weighted concentrations given further down in Table 1 (0.121 ug/m3 of PM2.5) what am I missing? It seems a bit confusing.

The values here refer to area-weighted concentration changes considering only populated grid cells (i.e. excluding large bodies of water and remote regions). Due to the correlation between impacts and population density, these area-weighted averages are lower than the population-weighted averages presented in Table 1. We have clarified this in the text (lines 103–104).

Previously, values in Table 1 considered only the population aged 30 years or older. For consistency with the rest of the manuscript (such as Fig. 4), we replaced the values with the

averages using all-age population as weights. This has a very small impact on the numbers, and does not change the interpretation of the results.

Fig 1 is very useful and informative. It is clear from the figure that the largest contributions from aviation to PM2.5 coincide with very populated regions.

We thank the reviewer for the compliment.

Lines 137 to 146: the role of non-Nox emissions in the PM2.5 estimates in interesting and particularly the role of S. The study looks at how different the estimates are if non-Nox emissions are not included but how significant are the non-S/non-Nox emissions? This is particularly interesting in the case of future very low or zero S fuels including some SAFs.

Although the effects of a switch to SAF were not the focus of our study, we still modelled a scenario of NO_x-only aircraft emissions, as those are the emission species that will most certainly remain with SAF, even with a sulfur-free fuel. Additionally, the $PM_{2.5}$ speciation in our results provide some indication of the contribution of each emitted species to overall air quality impacts (on a mass basis); we have added a sentence in the Discussion summarizing this (lines 393–395). But the significance of non-S/non-NO_x emissions with SAF depends on the specific fuel and engine conditions, so modeling those effects would require additional considerations in establishing scenarios for the analysis. We have also added another sentence in the Discussion (lines 406–409), with three additional references, pointing to efforts addressing these questions.

Lines 179 to 184 and the figure 2 do not add much to the understanding without a better explanation of how much of the emissions of Nox come from these categories etc.

We have added a sentence including the share of emissions associated with each category (lines 184–185).

I found Figure 4 hard to understand. On the RHS as the average population exposure changes for 2019 didn't match up with the data in table 1. Should they? I liked the sensitivity approach though and the way that the different future scenarios impact the results.

Figure 4 shows the air quality response to emissions on the left-hand side, while the right-hand subplots show impacts incorporating the effects of population. The most prominent results on the right-hand side of Figure 4, represented by the filled horizontal bars, are the average population exposure changes normalized by the amount of emissions, using 2040 as the baseline. As described in the figure's caption, the non-normalized values are represented by black vertical dashes overlaid on the horizontal bars. As the reviewer correctly noticed, these non-normalized values are the same metric as those presented in Table 1, but in the case of Figure 4, they consider only the coarser resolution simulations, without the nested domain simulations at finer resolution, as mentioned in the caption. This is done to ensure that only results of the same modeled resolution are presented. The global average exposure changes for 2019 considering only the coarser resolution (with nested domain results in parentheses) are: 0.105 $\mu g.m^{-3}$ (0.112 $\mu g.m^{-3}$) for $PM_{2.5}$, 0.825 ppbv (0.918 ppbv) for ozone, and −6.3 pptv (+11.0 pptv) for NO_2. We have added a sentence in the Discussion mentioning how health impact quantification (which is dependent on exposure) can be affected by model resolution (lines 353–356).

Lines 260 to 294 Actual aviation emissions estimates used for each of the years (2005, 2019, and 2040 base/high/low) in a table would have helped understand this section. Even in the methods section there is no aviation emission estimate data.

We have added a new Table 2 to provide an overview of emissions across the years and scenarios.

Lines 329 to 426 Discussion section

The authors duly note that "Uncertainty in the CRF propagates to the health impacts of aviation, so care must be taken when considering the absolute magnitude of impacts". And I agree that the relative evolution of the impacts over time in this study would not be affected by CRF. However, the statement that "the absolute numbers presented reflect the atmospheric changes that could be expected if all aircraft emissions were to be eliminated" is only true based on this particular modelling study with all the caveats applied.

The reviewer is right in pointing out the potential caveats that need to accompany this sentence. However, this sentence was not meant to highlight the absolute numbers themselves, but to instead caveat the fact that atmospheric nonlinearities may mean that our results cannot necessarily be scaled (beyond first order) to scenarios where emissions are only partly removed. We have updated this and the following sentence to avoid any misinterpretation (lines 336–341).

Lines 394 to 401 Here reference is made to ultra-low S fuel and this would be a very useful link to the significance of Sox emissions in the PM2.5 aerosol predictions made earlier.

That's a nice idea. We have added a sentence here (lines 393–395) to better link our results in terms of PM2.5 composition and the discussion on low-sulfur fuels.

Lines 401 to 410 This is a very important point regarding the relative toxicity of BC. The local air quality impacts of PM2.5 are based on mass, the contribution of nvPM or BC to mass concentrations is likely to be very low due to their very small size (aviation nvPM are less that 30nm). The toxicity of the various components of PM2.5 are not generally considered in these large epidemiological studies. The measurement of UFP is at an early stage compared to the ambient measurement of mass concentrations and as knowledge progresses the nature of the PM may become more relevant rather than purely the mass concentration.

We'd like to thank the reviewer for appreciating the point we're making in the Discussion (lines 402-412). To improve clarity, we have added an explicit mention that the CRF adopted uses $PM_{2.5}$ mass (lines 402–404).

Methods section on aircraft emissions

This would really benefit from a table of emissions (either here or in the results section) rather than just a reference to another paper (I had to look it up). There is rather a lot of information that could be transferred to the SI too to improve readability. The input data on aviation emissions are pretty critical to the understanding of the paper. The data would also benefit from some contextual comparison to assess how the data compare with other studies.

We have added a new Table 2 immediately before Figure 3 to provide an overview of emissions across the years and scenarios. We agree that the input data is critical to the understanding of the paper, which is why we opted to include it in the main text instead of

the SI. To improve readability while keeping this important information, we have spilt the text of this subsection into smaller paragraphs.

While comparison to similar studies would be interesting, there is not much available in the literature for a good comparison. We do however have in the Supplementary Note 1 a longer discussion on the various parameters used in the projections, along with a brief sensitivity analysis. We have included a reference to this sensitivity analysis in the main text (lines 491–494).
