# Supplementary material for: Global air quality and human health impacts of growing aircraft emissions
Source: Commun Earth Environ. 2026 Jun 16;7(1):806. doi: 10.1038/s43247-026-03732-4 (PMC13630550; doi:10.1038/s43247-026-03732-4)
Supplement: Supplementary file 2 [file 43247_2026_3732_MOESM2_ESM.pdf]

Supplementary Information: Global air quality and human health impacts of growing aircraft emissions

Flávio D. A. Quadros[1], Rick Nelen[1], Mirjam Snellen[1], Irene C. Dedoussi[1,2]*

[1] Operations and Environment section, Faculty of Aerospace Engineering, Delft University of Technology, Delft 2629 HS, the Netherlands.

[2] Whittle Laboratory, Department of Engineering, University of Cambridge, Cambridge CB3 0DY, UK

* Corresponding author: icd23@cam.ac.uk

Supplementary Note 1: Detailed methodology of aircraft emissions projection

Projecting future aircraft fuel burn

Passenger air traffic is modeled by considering the revenue passenger-km (RPK) achieved by each aircraft type for each pair of origin and destination world regions. The definition of regions follows that used in the ICAO's long-term traffic forecast[1]. The OAG schedule data include available seats for each flight, allowing for the calculation of total available seat-km (ASK) for each aircraft type and regional pair. A global average passenger load factor of 81.7% is then estimated from the ratio of total ASK and the global RPK value published by ICAO[2]. This load factor is applied equally to all aircraft type and region combinations to estimate specific RPK values for 2018. Note that the choice of load factors (also for cargo, described ahead) do not impact fuel burn and emissions values, as everything is relative to the base year of 2018 and the factors are applied uniformly to all flights.

Likewise, cargo air traffic is modeled according to the cargo ton-km (CTK) specific to each aircraft type and flight origin regions. The OAG dataset used lists “typical” available freight ton-km (AFTK) for some flights, and for missing values a constant payload mass for each aircraft type is applied. These listed “freight” values are considered in this analysis as “cargo”, i.e. freight plus mail, so the available cargo ton-km (ACTK) is equal to the listed AFTK. A global average load factor of 49.3% is applied uniformly to calculate CTK, based on data from the International Air Transport Association (IATA)[3].

Calculations for passenger and cargo are done in parallel with the same approach, using RPK and CTK, respectively, as a metric of air traffic volume T (Equation 1). The 2018 values of traffic are projected forward to each year y by applying the regional-pair-specific compound annual growth rates ($r_{y,rgn}$) from the ICAO’s long-term forecast (ICAO lists values for freight, but those values are used here with no distinction being made between FTK and CTK) (Equation 2). Four forecast scenarios are considered: the medium traffic projection made prior to the COVID-19 pandemic (Pre-COVID)[4], and three projections made in July 2021 with different levels of recovery and growth (High, Mid, Low)[1]. Constant growth rates are given for the periods from 2018 to 2028, 2038, and 2048. For the three post-COVID cases, the pre-COVID forecast is applied between 2018 and 2019, and zero RPK growth is applied between 2019 and 2023 (High), 2024 (Mid), or 2027 (Low), with growth resuming at a pace that matches the forecast traffic for 2028 in each scenario. Likewise, CTK growth is set to zero between 2019 and 2021 (High), 2022 (Mid), or 2023 (Low). This is done to reflect in the model that aircraft were not scrapped en-mass during the pandemic and then replaced with newer aircraft once traffic returned; the lower traffic is not modeled and that period is thus disregarded in the results. Projections are made up to the year 2040. As a simplification, the extra length of leap years is not taken into account, effectively underestimating

traffic in those years by 0.3%. Since ICAO's air-traffic forecasts concern only total passenger and cargo traffic and there is no consensus on how this new demand will be spread in the various segments of the aircraft market, the proportion of traffic met by each class of aircraft is kept constant from 2018 onwards (Equation 3). Although the present trend of growing market share by low-cost carriers, if continued, might drive a shift in these proportions[5].

$$T = \{\mathrm{RPK}, \mathrm{CTK}\} \tag{1}$$

$$T_{y,rgn} = r_{y-1,rgn} \cdot T_{y-1,rgn} \tag{2}$$

$$T_{y,rgn,class} = T_{y,rgn} \cdot \frac{T_{y=2018,rgn,class}}{T_{y=2018,rgn}} \tag{3}$$

At each year, the projected traffic demand is met by a combination of aircraft that were already operating, of various types i, and aircraft were introduced that year, of each generic $class$ (Equation 4). The traffic demand fulfilled by already existing aircraft is calculated as the same as the previous year scaled to the expected fraction of aircraft that remain in service (Equation 5). A generic aircraft of each class, with up-to-date performance, is introduced every year to make up the difference between the total traffic projected and the traffic achieved by the aircraft that remain operating (Equation 6). The steps described by Equations 4–6 are applied to each regional pair of origin and destination.

$$T_y = \left(\sum T_y, i\right)_{old} + \left(\sum T_y, class\right)_{new} \tag{4}$$

$$T_{y,i,old} = T_{y-1,i,old} \cdot (1 - ret_i) \tag{5}$$

$$T_{y,class,new} = T_{y,class} - \left(T_{y,i,old}\right)_{class} \tag{6}$$

Fleet renewal is modeled by applying, each year, a class-specific retirement rate to the current age distribution of each aircraft type. The fraction of a type's fleet that retires in a year (ret_i) is calculated according to Equation 7, where $N_{i,t}$ denotes the number of aircraft of age t at year i. Retirement rates are modeled as a logistic function (Equation 8), using the parameters (a_1, a_2) given by ref. [6]. In these functions, the median retirement ages are 27.7, 28.9, 29.5, and 33.2 years for widebody, narrowbody, regional jet, and turboprop classes respectively. The initial aircraft age distribution (for 2018) is primarily estimated by applying the retirement model to historic annual delivery numbers from Boeing, Airbus, Embraer, and Bombardier. Additionally, the initial ages of some aircraft types are estimated based on the entries present in the national aircraft registries of the US, Brazil, and Spain. If age data for a type are still insufficient, online crowdsourced aircraft databases (such as https://www.planespotters.net/) are used, or a constant delivery rate during the years of production is assumed. Applying the same aircraft type-specific retirement rate across all regions assumes age distributions are also uniform across regions, which is a simplification. However, the starting state of the model represents different regional makeups in the amount of traffic achieved through each aircraft type.

$$\mathrm{ret}_i = \sum_t \left(\mathrm{ret}_{class,t} \frac{N_{i,t}}{N_i} \right) \tag{7}$$

$$\mathrm{ret}_{class,t} = \exp\left(-a_{1,class} - t \cdot a_{2,class}\right) \tag{8}$$

To evaluate the impact of retirement rate on the results, four alternative not necessarily realistic scenarios are considered: faster retirement, slower retirement, full fleet annual replacement, and no retirement whatsoever. For the faster and slower cases, the a_1 parameters were adjusted so that the medians retirement ages were reduced or delayed by 10 years (Supplementary Fig. 1).

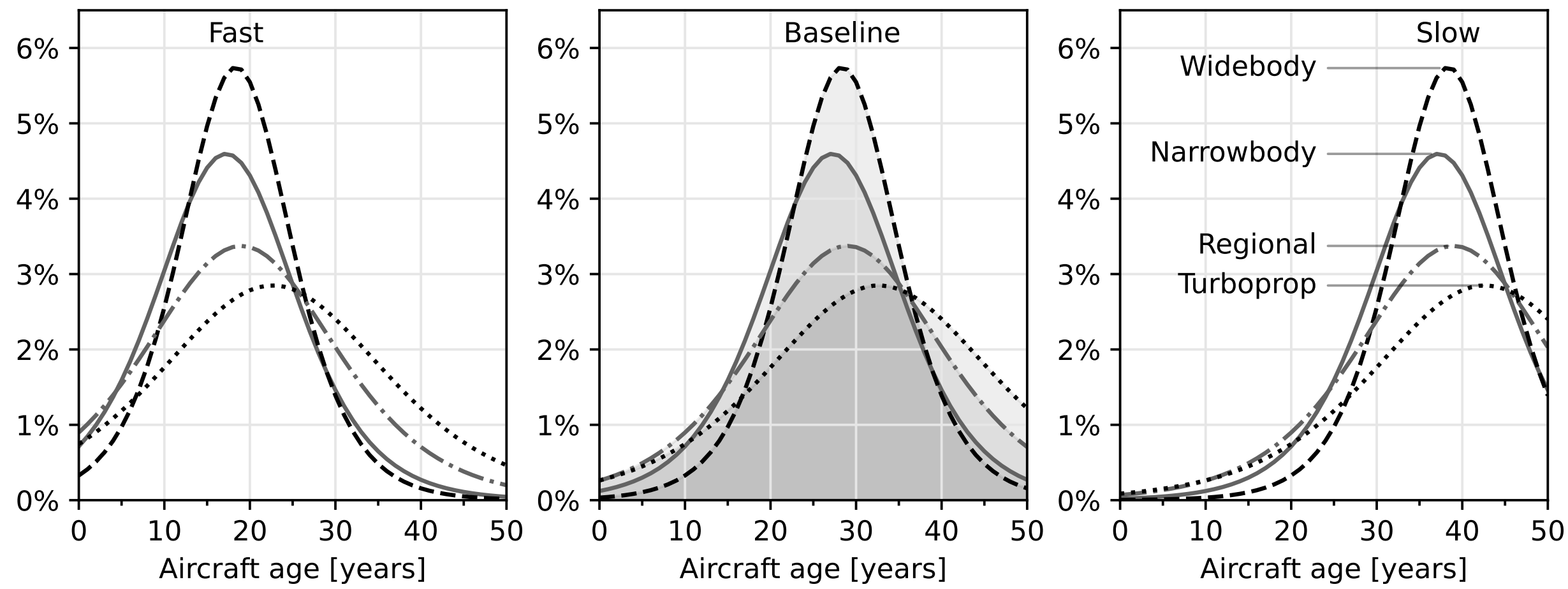

Supplementary Fig. 1: Probability mass function of retirement age. Rates are given for annual intervals (% · yr^{-1}). In addition to the baseline rates[6], a faster and slower alternative scenarios are presented.

Specific fuel consumption (c) is quantified for 2018 for each aircraft type by the ratio between fuel burn (C) and the traffic metric. These values are calculated globally, as some aircraft types might fly only a low number of routes for a given pair of regions which might not be representative of the aircraft performance if the routes were to change within the same regional pair. The traffic-specific fuel consumption values calculated for 2018 are then scaled every year by the expected average reduction due to operational improvements, k_{op} (Equation 9).

$$c_{y,i} = \begin{cases} \frac{C_{y,i}}{T_{y,i}} & y = 2018 \\ c_{y-1,i} \cdot \left(1 - k_{op,y-1}\right) & y > 2018 \end{cases} \tag{9}$$

k_{op} is calculated according to the two sets of goals set in 2014 by a panel of independent experts under the ICAO CAEP (Committee on Aviation Environmental Protection)[7]. The baseline goals are cumulative improvements relative to 2010 of 3.25% (2020), 6.75% (2030), and 9.00% (2040) as shown in Supplementary Fig. 2. The other set of goals established are referred by the panel as "lower limits of the confidence range", which are denominated here as the "low" scenario for

operational fuel consumption improvements. For both scenarios, no further improvements are considered beyond the year 2040, which is an assumption that all possible efficiency improvements in operations will have been realized by then.

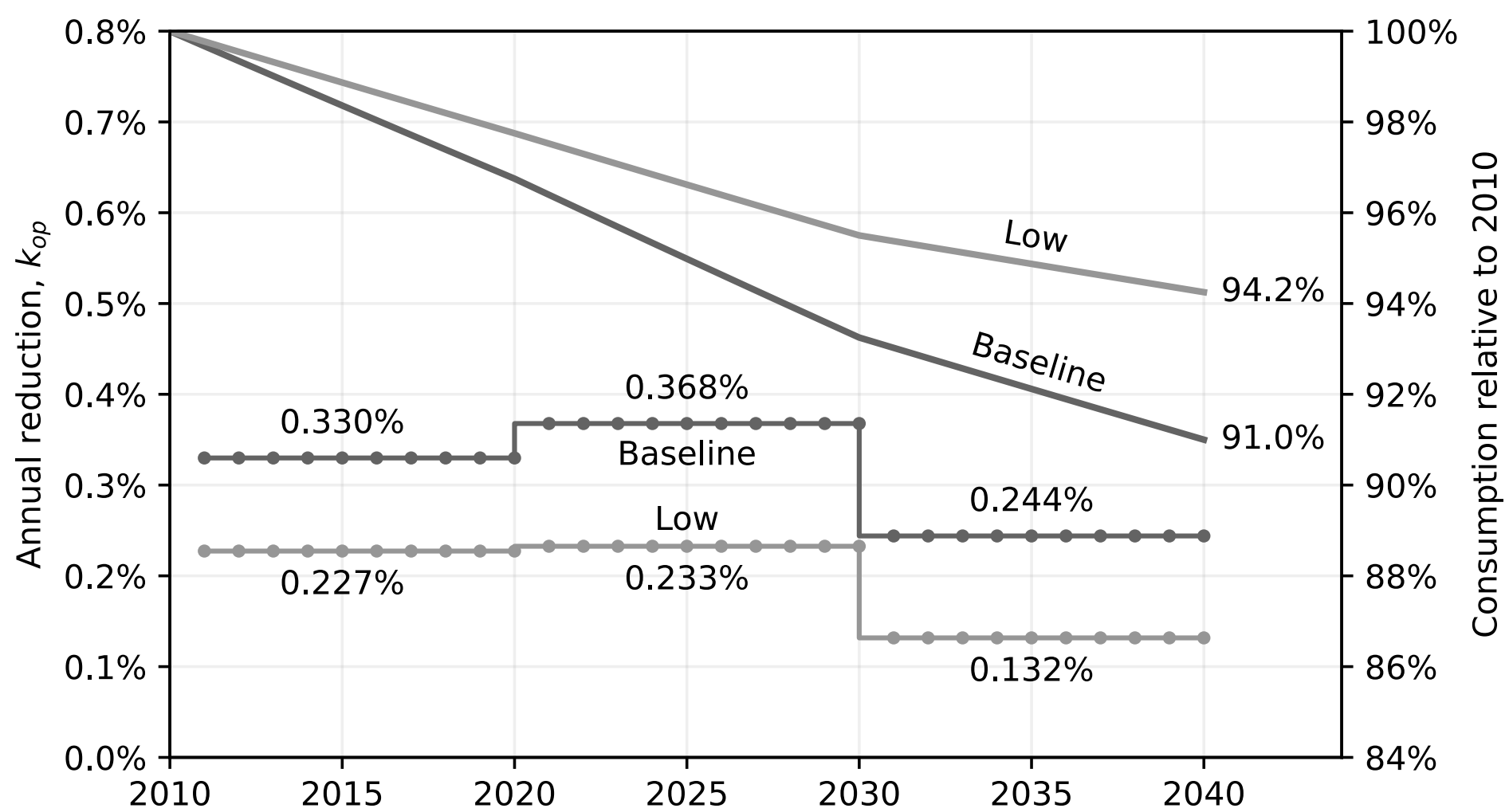

Supplementary Fig. 2: Scenarios of reduction in fuel consumption due to operational improvements. Annual values compound to the goals from ref. [7].

A set of reference aircraft are chosen to represent the present-day performance typical of new aircraft from each class, serving as the starting point for future projection (Supplementary Table 1). These values are taken as the mean of two representative aircraft types, for which the average performance values are calculated across all passenger or cargo flights in 2018. As an exception, only one reference turboprop type is used for cargo, due to the small number of flights available. Supplementary Table 1 lists the engine models used to calculate emissions for each reference aircraft type. Specific fuel consumption of future aircraft, in addition to being adjusted by k_{op}, is also adjusted by a factor to account for technological improvements, k_{tec} (Equation 10).

Supplementary Table 1: Reference aircraft types and engines used to represent present technology level. The engine models adopted for each type are listed, with the ICAO's Engine Emissions Databank unique identifier shown for the three classes of jet aircraft. For types with multiple engine families, the weights used for averaging fuel consumption and emissions are shown in parentheses. Engine assignments are based on ref. [8].

Class	Passenger		Cargo	
	Type code	Engine	Type code	Engine
Widebody	A359	01P18RR124	A332	01P14RR102 (68%)<br>4GE081 (17%)<br>9PW094 (15%)
	B78X	01P17GE214 (69%)<br>02P23RR132 (31%)	B77L	01P21GE216
Narrowbody	A20N	01P20CM128 (53%)<br>01P22PW163 (47%)	A320	01P08CM105 (58%)<br>01P10IA021 (42%)
	B38M	01P20CM136	B737	01P11CM114
Regional	E290	01P20PW187	n/a	
	BCS3	01P20PW182		
Turboprop	AT72	PW121	AT72	PW121
	DH8D	PW127E	n/a	

$$c_{y,class,new} = \begin{cases} \text{mean}\left(c_{y=2018,i}\right)_{i \in ref,class} & y = 2018 \\ c_{y-1,class,new} \cdot \left(1 - k_{op,y-1}\right) \cdot \left(1 - k_{tec,y-1}\right) & y > 2018 \end{cases} \quad (10)$$

For k_{tec}, the adopted values are the same as used by CAEP for the long-term trends analysis published in their Environmental Report 2019[9]. Four scenarios are considered, with a constant annual fuel reduction applicable to all aircraft classes: 0.57% (low), 0.96% (moderate), 1.16% (advanced), and 1.50% (optimistic). Another scenario adopted here, labeled "IE", consists of the aircraft class-specific values found by a panel of independent experts under CAEP from 2017, which were also published in 2019[10], shown in Supplementary Fig. 3. The source for this last scenario does not consider turboprop improvements, which is thus set to zero in this analysis. This

is not expected to have a large effect, as the fraction of total fuel burn attributed to turboprops is very small (~1–2%).

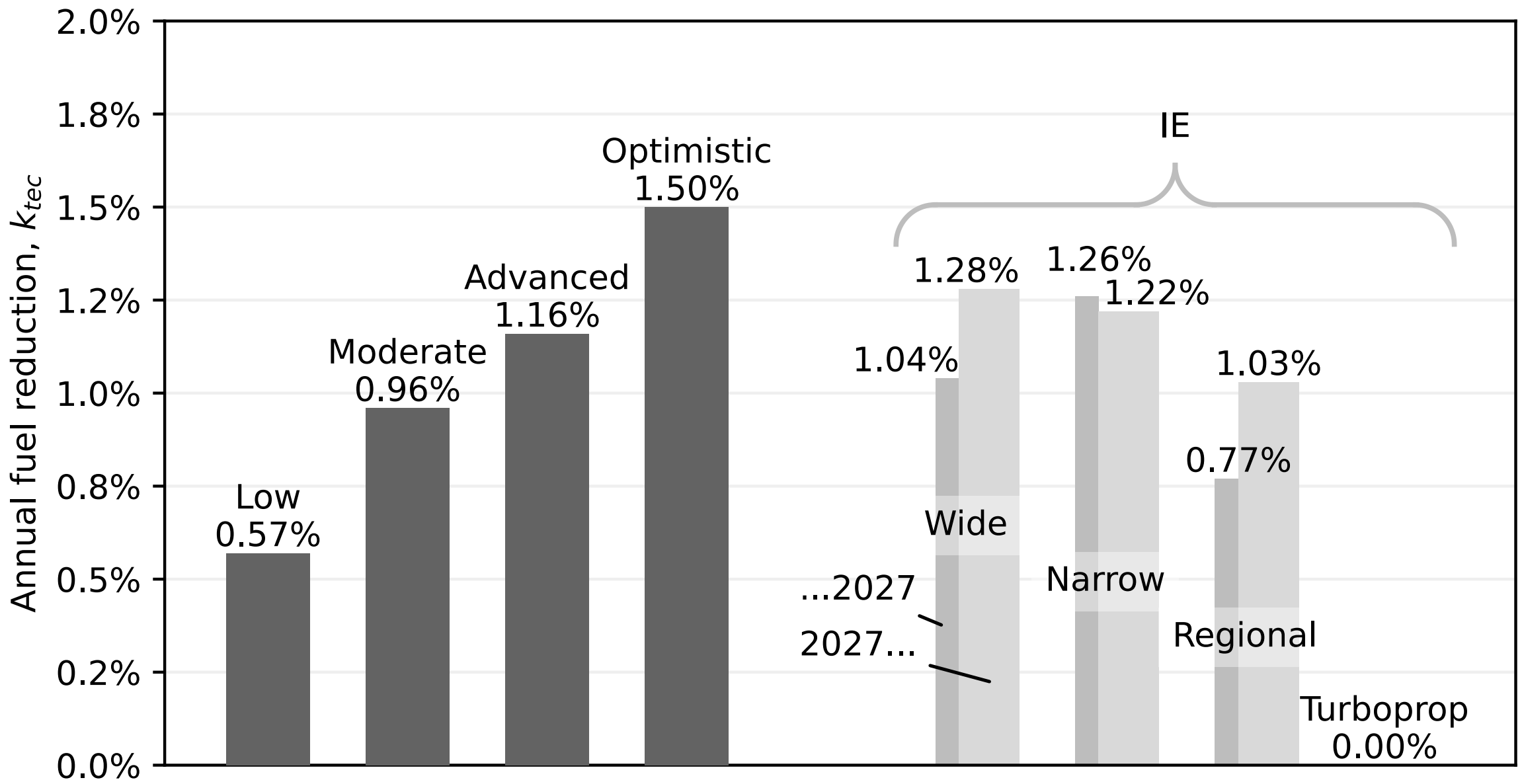

Supplementary Fig. 3: Scenarios of reduction in fuel consumption due to aircraft technological improvements. Values are based on ref. [9] (Low, Moderate, Advanced, Optimistic) and ref. [10] (IE).

Finally, the total amount of fuel burned in future years is determined by the combination of traffic and specific fuel consumption projections for each aircraft type, both existing and generic future types (Equation 11). Note that the fleet of generic aircraft introduced each year continues in the calculation for future years, being subjected to the retirement and operational improvement functions described previously.

$$C_y = \sum c_{y,i} \cdot T_{y,i} + \sum c_{y,class,new} \cdot T_{y,class,new} \qquad (11)$$

Projecting future aircraft NO_x emissions

The starting point of NO_x projections is the average emission indices (EI) and engine certification emissions results of the reference aircraft (Supplementary Table 1). These EI values are then projected forward according to technology improvements quantified as a ratio (k_{NO_x}) to the goal for the year 2027 proposed by CAEP's 2017 independent expert integrated review panel[10]. As the panel did not consider turboprops, emission indices for this class are frozen at the values of the reference aircraft in 2018, assuming no change in technology or engine pressure ratio. In the same format as the ICAO emission standards[11], the goal gives the mass of emissions in the standard certification cycle (D_p) normalized by engine rated thrust (F_{00}) as a function of the engine's overall pressure ratio (OPR, π_{00}). Equations (12) and (13). show these calculation steps.

$$\left(\frac{D_p}{F_{00}}\right)_{y,class} = \left(5.75 + 0.577 \cdot (\pi_{00})_{y,class}\right) \cdot \left(k_{\mathrm{NO}_x}\right)_{y,class} \tag{12}$$

$$\mathrm{EI}(\mathrm{NO}_x)_{y,class} = \mathrm{EI}(\mathrm{NO}_x)_{ref,class} \frac{\left(D_p/F_{00}\right)_{y,class}}{\left(D_p/F_{00}\right)_{ref,class}} \tag{13}$$

k_{NO_x} varies per year, representing technological improvement, with the values for 2018 chosen to match how far the modeled reference aircraft are to the 2027 goal. k_{NO_x} then linearly goes to unity in 2027 for all classes of aircraft. Three scenarios are considered in terms of what happens beyond: no further improvement (baseline), emissions cut by another 10% by 2037 (continued-conservative), and emissions cut by another 50% by 2047 (conservative-optimistic). A fourth (unrealistic) scenario consists of no improvements from 2018 (Supplementary Fig. 4).

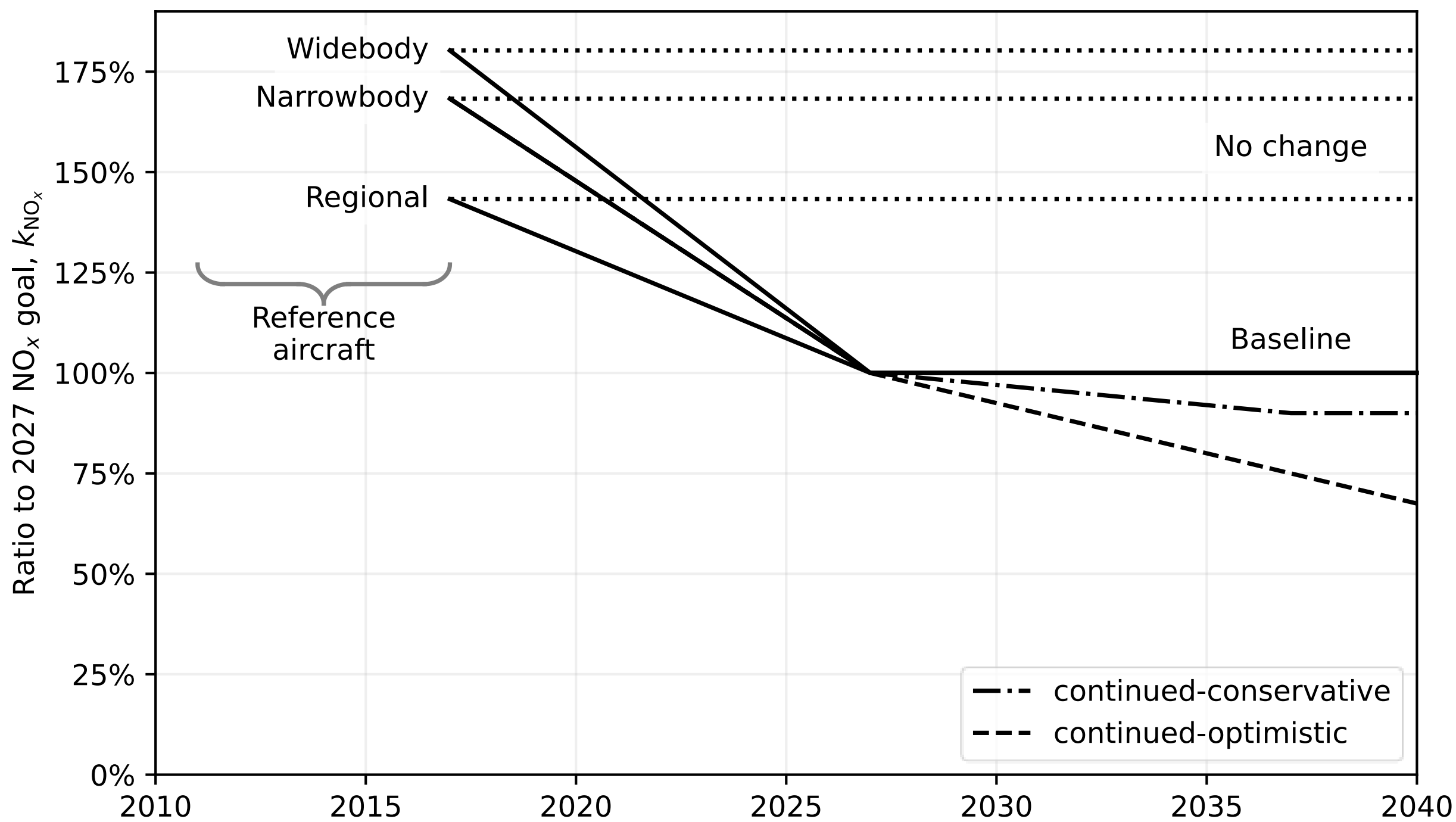

Supplementary Fig. 4: Scenarios of NO_x technology improvements. Values are shown as a ratio to the 2027 goal.

As the baseline estimate, π_{00} is considered to increase constantly up to the values of 60, 65, and 70 in 2027 for regional jets, narrowbodies, and widebodies, respectively, as shown in Supplementary Fig. 5. This scheme represents a new generation of turbofans with increased pressure ratios being introduced 10 years after the base year for the technology forecasts, with no further increases afterwards. Alternative scenarios considered include a delayed (to 2037) introduction of this new generation of engines (Low scenario), and a scenario in which OPR increases a second time from 2027 to 2037 (High), representing a second future generation of engines. While the OPR value affects the NO_x emissions, it also impacts the overall engine efficiency, and thus the resulting fuel burn. Note that this later effect is not captured in this modeling chain, and that aircraft and engine technological improvements are solely represented in terms of the overall fuel efficiency technology targets as stated in ICAO's 2017 independent expert integrated review panel[10]. This mismatch is not expected to introduce significant uncertainty in the

estimated EIs, but future work could focus on coupling projections of fuel efficiency improvements to OPR.

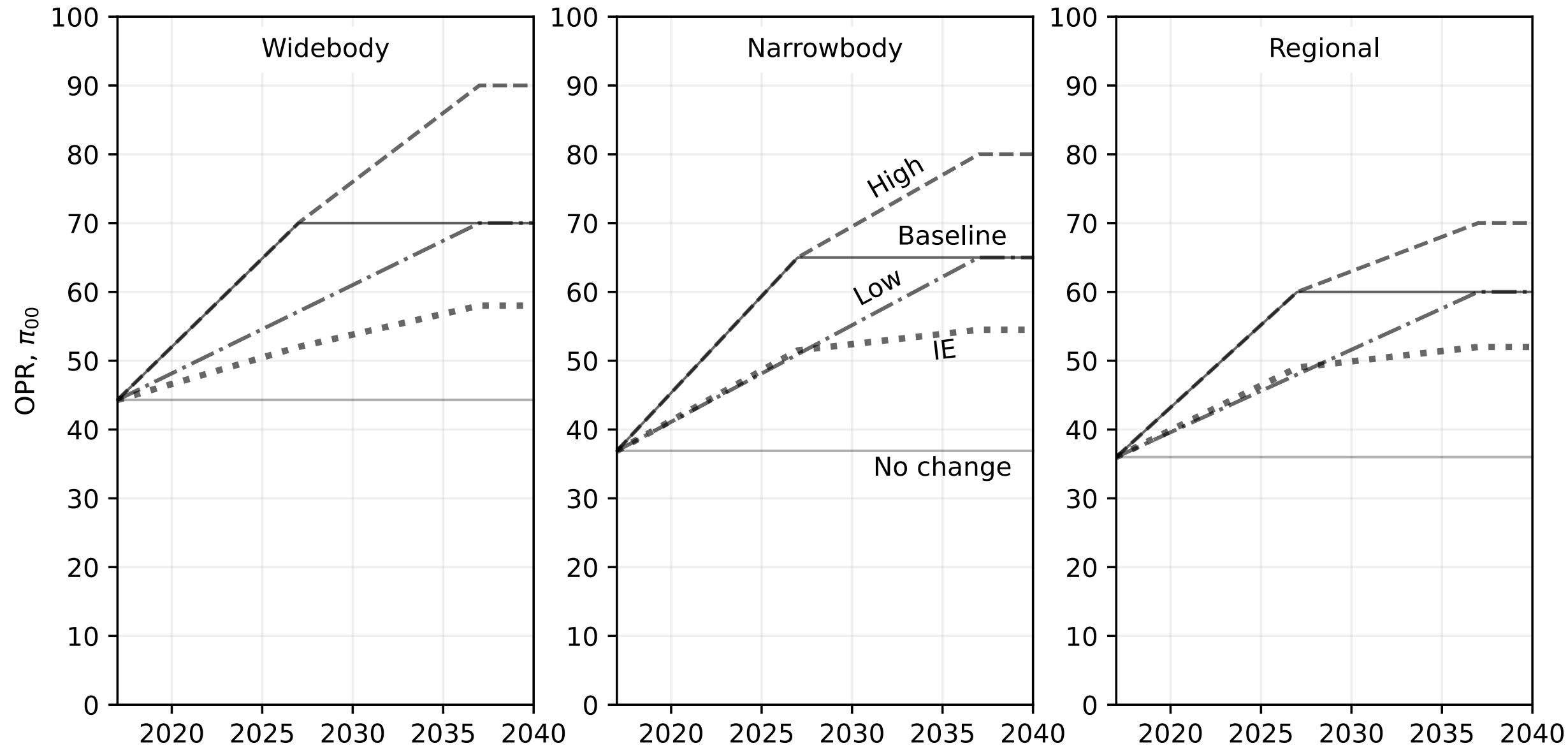

Supplementary Fig. 5: Scenarios of overall engine pressure ratio for future aircraft. "IE" scenario based on ref. [10].

Overall scenarios considered

Throughout this supplementary note, various scenarios for individual model parameters were described. From a baseline setting, each parameter is changed individually to evaluate the sensitivity of the results to the input changes. Additionally, two other overall scenarios are evaluated, consisting of different selections of input parameters as listed in Supplementary Table 2: Low and High (emissions). While subjective, these selections allow the production of some form of an overall realistic lower and upper boundary of future emissions.

Supplementary Table 2: Parameter selection for the overall scenarios of emissions projections.

Parameter	Overall scenario			Other parameter options
	Low	Baseline	High	
T	Low	Mid	High	Pre-COVID
ret	Baseline	Baseline	Baseline	Fast, Slow, Annual replacement, No retirement
k_{op}	Baseline	Baseline	Low	No change
k_{tech}	Advanced	Moderate	Low	Optimistic, IE, No change
k_{NO_x}	Continued-conservative	Baseline	Baseline	Continued-optimistic, No change
π_{00}	Low	Baseline	High	IE, No change

Sensitivity of future fuel burn and NO_x emissions to different forecast parameters

Supplementary Table 3 presents the relative changes in the aggregate fuel burn and NO_x emissions over the period of 2018 through 2040 as a result of different individual modeling choices, changing parameters one at a time from the overall baseline scenario (Supplementary Table 2). Overall, individual scenario choices can result in total fuel burn variations of −17% to +10%, total NO_x emissions variations of −18% to +36%, and the time-averaged EI (NO_x) and the

EI (NO_x) in 2040 varying by −18% to +36% and −25% to +51%, respectively. The two alternative traffic forecasts from ICAO result in −17% and +8% NO_x emissions by themselves.

While faster retirement rates would reduce emissions by accelerating the adoption of more efficient aircraft, the likely unrealistic reduction of median retirement age by 10 years leads only to a reduction of 5% of emissions over the 2027–2040 period. And the more extreme hypothetical of replacing all aircraft every year reduces NO_x by 17%. The baseline projected operational and technological improvements avoid 6% and 7% of fuel burn, respectively (compared to the "no change" scenarios). The optimistic technology forecast would reduce fuel burn by another 4% relative to the baseline.

The largest variations in NO_x emissions are introduced by the NO_x technology and OPR scenario selection, which directly affect EI (NO_x). The baseline scenario, considering OPRs of 70:1 (widebodies) and 65:1 (narrowbodies) in 2027, lead to a 33% higher EI (NO_x) in 2040 relative to not changing the OPR further from the present reference aircraft, which are in the 33:1 to 48:1 range. Higher pressure ratios are compensated by the NO_x technology improvement parameter, with the 2040 fleet average EI (NO_x) being 34% for the baseline case relative to considering no change in k_{NO_x}. The "continued-optimistic" scenario, which projects another halving of NO_x emissions for a given OPR from 2027 to 2047, would reduce the fleet average EI (NO_x) in 2040 by 11% relative to the baseline.

Supplementary Table 3: Effects of different modeling parameter choices on future NO_x and fuel burn projections. Effects of changing on parameter at a time from the baseline estimate are given as relative differences of total fuel burn and NO_x emissions between 2027 and 2040, as well as the estimated global fleet EI (NO_x) averaged over 2027–2040 and the estimated global fleet average EI (NO_x) for 2040.

Parameter (baseline)	Scenario	2027–2040 period [%]			2040 [%]
		Fuel	NO_x	EI (NO_x)	EI (NO_x)
Traffic (Mid)	Low	−16.8	−17.0	−0.3	−0.2
	High	+7.6	+7.9	+0.3	+0.2
	Pre-COVID	+9.6	+11.1	+1.3	+1.1
Retirement (Baseline)	No retirement	+8.3	+5.8	−2.3	−2.3
	Slow	+4.8	+3.4	−1.3	−1.0
	Fast	−5.7	−4.5	+1.2	+0.2
	Annual replacement	−16.3	−16.9	−0.7	−0.9
Operations (Baseline)	No change	+5.5	+5.5	0.0	0.0
	Low	+2.1	+2.1	0.0	0.0
Fuel burn technology (Moderate)	No change	+7.4	+7.4	0.0	0.0
	IE	−1.3	−1.3	0.0	−0.1
	Low	+2.9	+2.9	0.0	0.0
	Advanced	−1.4	−1.4	0.0	0.0
	Optimistic	−3.7	−3.7	0.0	0.0
NO_x technology (Baseline)	No change	0.0	+36.0	+36.0	+50.8
	Continued-conservative	0.0	−1.9	−1.9	−4.0
	Continued-optimistic	0.0	−5.0	−5.0	−10.7
OPR (Baseline)	No change	0.0	−17.9	−17.9	−24.6
	IE	0.0	−9.4	−9.4	−12.1
	Low	0.0	−5.6	−5.6	−5.4
	High	0.0	+4.3	+4.3	+8.9

Supplementary Note 2: Comparison of atmospheric model with observations

The GEOS-Chem model is an open-source toolset, continuously developed by the community of hundreds of research groups worldwide that use it for various applications. This development is managed by working groups with long-term priorities guided by an international steering committee. With every new major release a series of benchmark simulations are performed comparing the new and old versions of the model, as well as comparing model output with atmospheric measurements. The results from this benchmark for the model version used in this study was published on https://wiki.seas.harvard.edu/geos-chem/index.php/GEOS-Chem_13.3.0#1-year_full-chemistry_benchmarks, accessed 25 September 2024.

In complement to that, we present here comparisons of the modeled output used in this study against measurements from air quality ground stations and ozonesondes, all for the year 2019. Comparisons against aircraft measurement campaigns are also briefly discussed here. As these benchmarks compare background concentrations, they are indicative of GEOS-Chem's ability of producing a representative model of the atmosphere. A more direct validation of the model's response to perturbation from aircraft emissions is not possible as there is no such direct measurement available. Development of the GEOS-Chem model aims to improve over time the accuracy of both inputs (such as meteorological fields and emission rates) and outputs (such as species concentrations), such that good agreement to observed concentrations while emissions estimates change over time is at least an indirect indicator of the accuracy of the model's response to emissions.

Ozonesonde data from around the world are obtained from the World Ozone and Ultraviolet Radiation Data Centre (WOUDC)[12]. Ground-station data are obtained from multiple sources. For the United States, data come from the US Environmental Protection Agency's (EPA) Air Quality

System (AQS)[13]. For Canada, data come from the National Air Pollution Surveillance (NAPS) Program, managed by Environment and Climate Change Canada (ECCC)[14]. For Europe, data come from the AQ e-Reporting platform from the European Environment Agency (EEA)[15]. For India, data come from the Central Pollution Control Board (CPCB); both from a network of manual stations of the National Ambient Air Quality Monitoring Program (NAMP)[16], and a network of automated stations Continuous Ambient Air Quality Monitoring (CAAQM). Since only raw data is available from CAAQM, the dataset processed by Sharma and Mauzerall is used[17]. For China, data come from the China National Centre for Environmental Monitoring (CNEMC). As archival or validated data from CNEMC is not available, the dataset processed by Silver et al. is used[18]. For other countries in East and Southeast Asia, data come from the Acid Deposition Monitoring Network in East Asia (EANET)[19].

We find that GEOS-Chem underestimates background ozone mixing ratios relative to ozonesonde measurements. For surface and cruise altitudes, the average underestimation is between 3% and 28% (4). This is consistent with a previous comparison of a tropospheric simulation of GEOS-Chem (v12.7) against the KORUS-AQ airborne measurement campaign, which found an average normalized mean bias of −26% considering the entire flights and −28% considering only altitudes below 1.5 km[20]. As ref. [20] discusses, such underestimates might be due to several factors, such as low modeled ozone influx from the stratosphere and low ozone production in how VOC (volatile organic compounds) chemistry is modeled, including inadequate modeling of organic nitrogen species. The GEOS-Chem simulations we perform in this study include stratospheric chemistry, which yield stratospheric ozone mixing ratios similar to those from ozonesondes, as observed both in Supplementary Fig. 6 and in ref [21]. Since we observe most of the (ground-level) air quality impacts from aircraft being from cruise emissions, we expect that

an underestimate of vertical transport and mixing should lead to an underestimate of these impacts (increase in ground-level ozone and $PM_{2.5}$ and decrease in NO_2).

The comparison against KORUS-AQ showed a smaller (relative to ozone) underestimate of tropospheric NO_x, −5%[20]. If GEOS-Chem is underestimating ozone formation due to NO_x-VOC photochemistry in the free troposphere, we would expect that aircraft-attributed ground-level impacts would likewise be underestimated, based on the prominent role that formation of ozone and organic nitrogen compounds (such as PAN, peroxyacyl nitrates) from NO_x is believed to have in carrying effects from cruise emissions to the surface[22]. The complexities of how changes to the chemistry model affect overall concentrations of various species are illustrated by an update on GEOS-Chem chemistry model introduced after the version used in this study, with changes in reactions of reactive nitrogen species leading to net decreases of 1.7%, 0.7%, and 3.8% in NO_x, ozone, and PAN, respectively, along with a 7% net increase in stratospheric NO_x[23].

It should be noted that in this section we compare modeled background concentrations with observations, and differences can be due any combination of errors in sources and sinks of pollutants, including emissions, chemistry, and transport. Therefore, while this comparison quantifies how the model is performing in simulating the total composition of the atmosphere, it does not explicitly quantify the accuracy of changes in concentrations attributable to marginal emissions from aircraft.

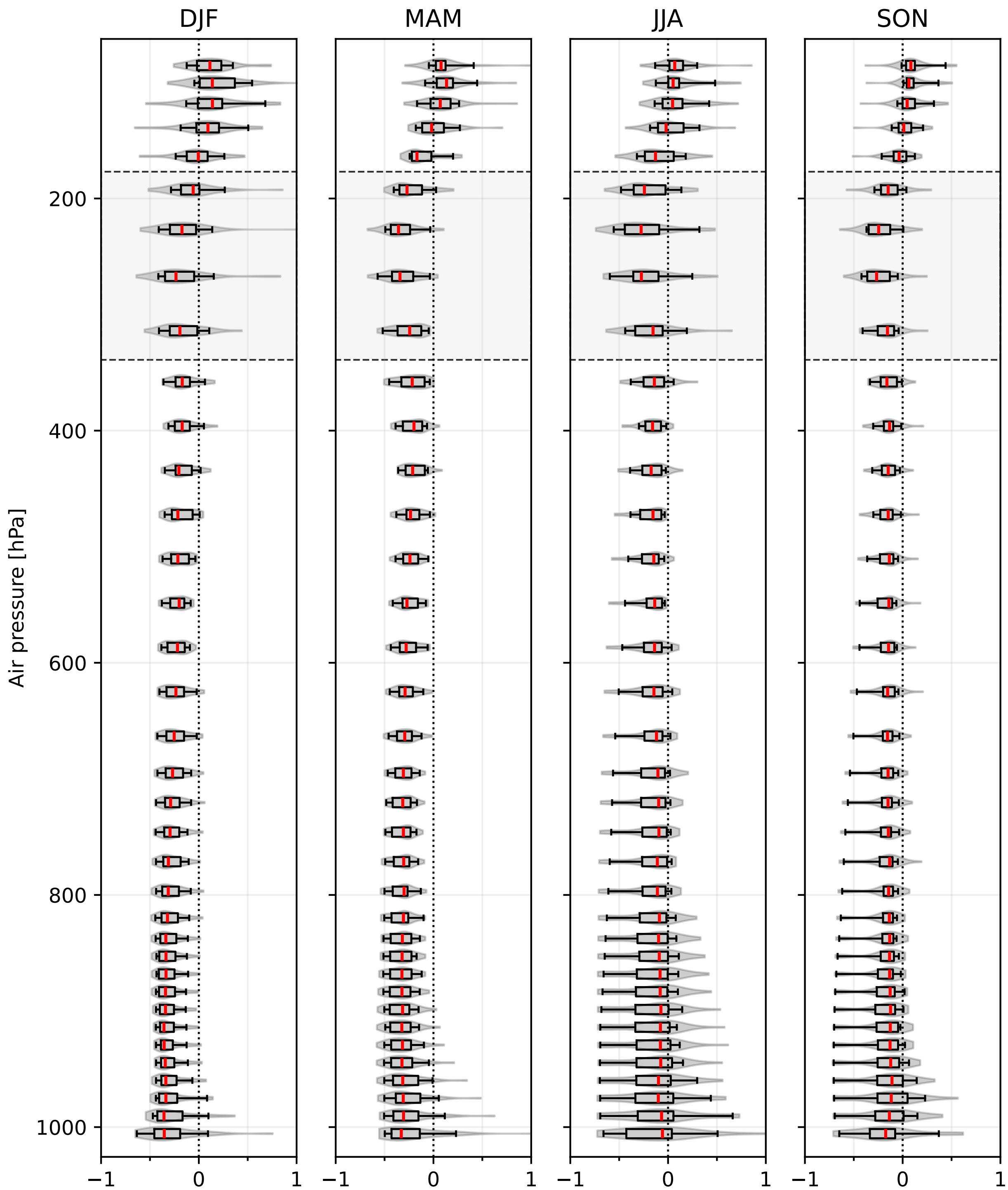

Supplementary Fig. 6: Comparison of modeled and observed ozone vertical profile. Distribution of relative difference of seasonal average ozone dry mixing ratio between the model and measurements from ozonesondes in 2019[12]. DJF = December-January-February, MAM = March-April-May, etc. Box plots indicate 5th, 25th, 50th, and 95th percentiles. Shaded pressure range indicate typical cruise altitudes, where ~70% of modeled aircraft NO_x are released.

Supplementary Table 4: Average relative differences in ozone mixing ratio between model and ozonesondes. Average relative differences at the lowest vertical level and at cruise altitude, defined as the 4 vertical levels with the highest aircraft NO_x emissions. Ozonesonde data from 2019[12].

Season	Surface	Cruise
December-January-February	−28%	−12%
March-April-May	−20%	−28%
June-July-August	−3%	−20%
September-October-November	−18%	−19%

Compared to ground-based observations, we find that GEOS-Chem has smaller variations between different locations (Supplementary Fig. 7–8). This is consistent with the fact that each modeled grid cell is has an area on the order of thousands of square kilometers, over which a single "average" concentration value is considered. Over Europe and North America, the model has median biases of −8% to +9% for ozone, 0% to +28% for $PM_{2.5}$, and −62% to −44% for NO_2 (Supplementary Fig. 9). Over Asia, median biases are −2% to +147% for ozone, +3% to +40% for $PM_{2.5}$, and −65% to −44% for NO_2 (Supplementary Fig. 10). Significantly higher ozone concentrations are found in the model relative to the automated measurements in India, although we note that this observation dataset from CAAQM is based on raw data reported, unlike the other sets which underwent data quality and validation by the responsible institutions (except also the CNEMC dataset). The larger discrepancies in Asia outside China are consistent with GEOS-Chem development having been historically done by researchers in Europe, North America, and China, and with air quality measurement data in Asia being having worse availability and being less open[17,18]. The spatial distribution of measurements in these comparisons is shown in Supplementary Fig. 11–13. Supplementary Table 5–7 list additional statistical metrics comparing ground-based observations with modeled values.

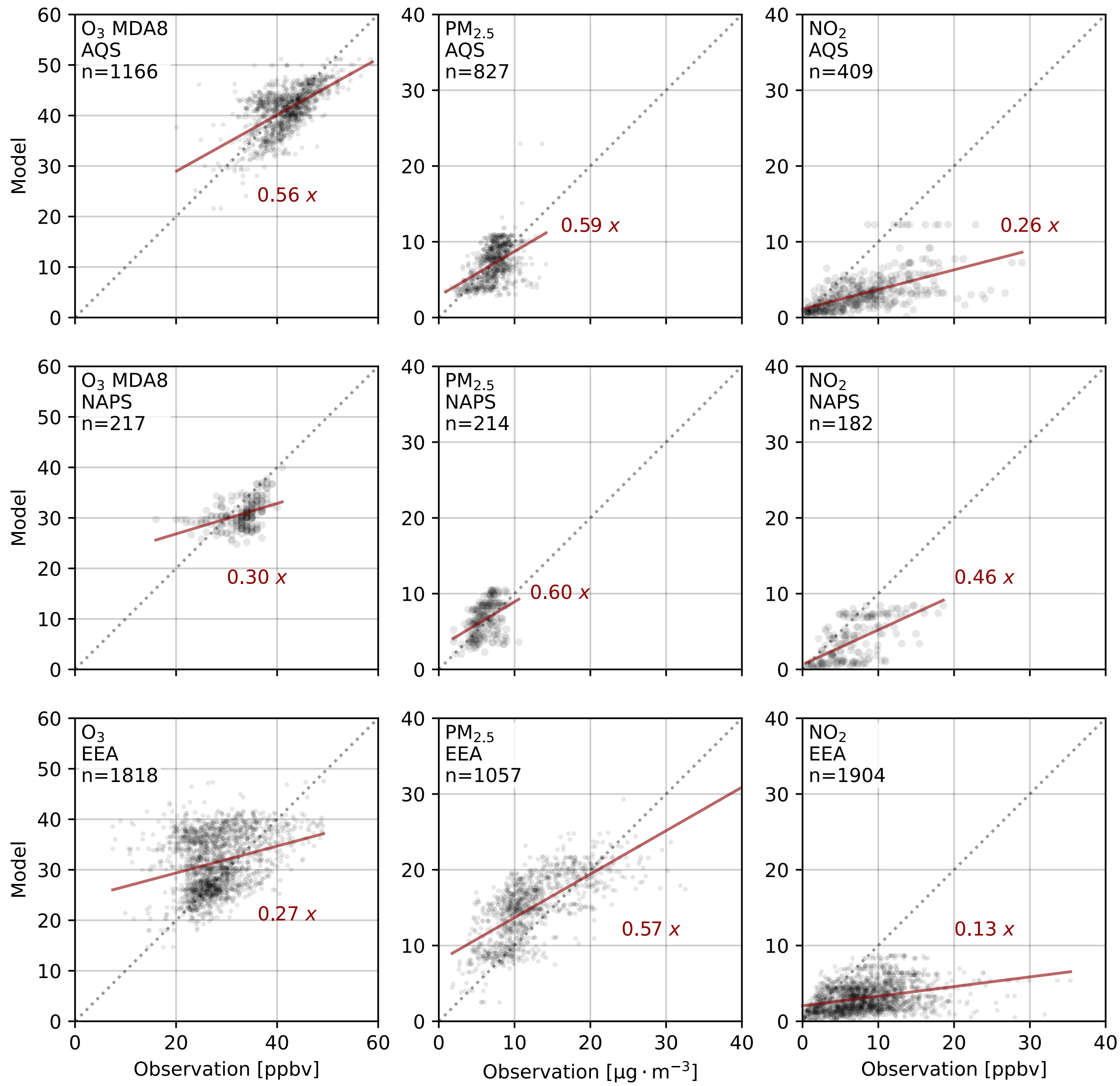

Supplementary Fig. 7: Scatter plot of modeled and observed air quality metrics in Europe and North America. Values are 2019 annual averages. Each point is a measurement station. Slopes of linear regressions are indicated in maroon. Datasets from AQS (US)[13], NAPS (Canada)[14], and EEA (Europe)[15]

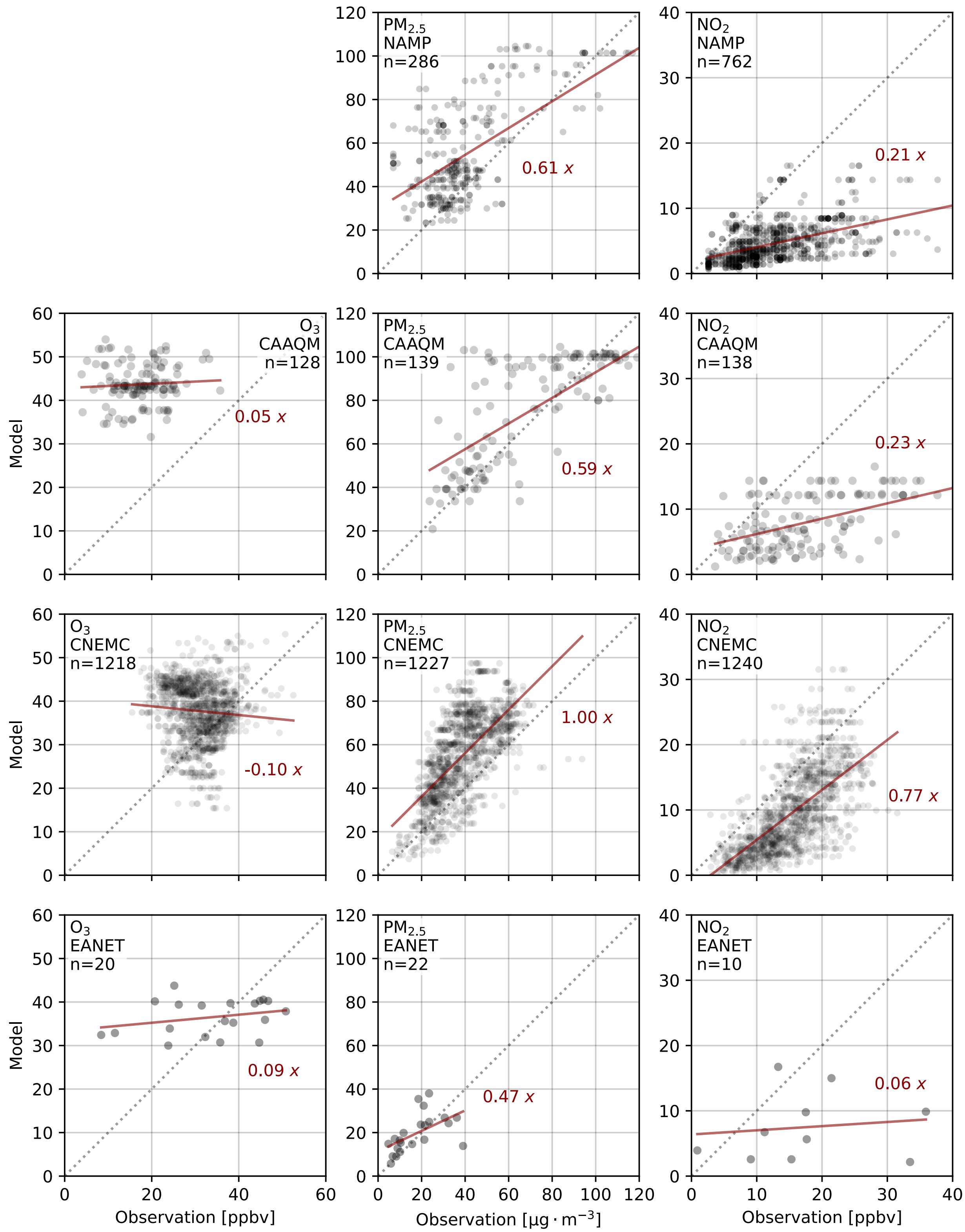

Supplementary Fig. 8: Scatter plot of modeled and observed air quality metrics in Asia. Values are 2019 annual averages. Each point is a measurement station. Slopes of linear regressions are indicated in maroon. Datasets from NAMP (India)[16], CAAQM (India)[17], CNEMC (China)[18], and EANET (East and Southeast Asia)[19].

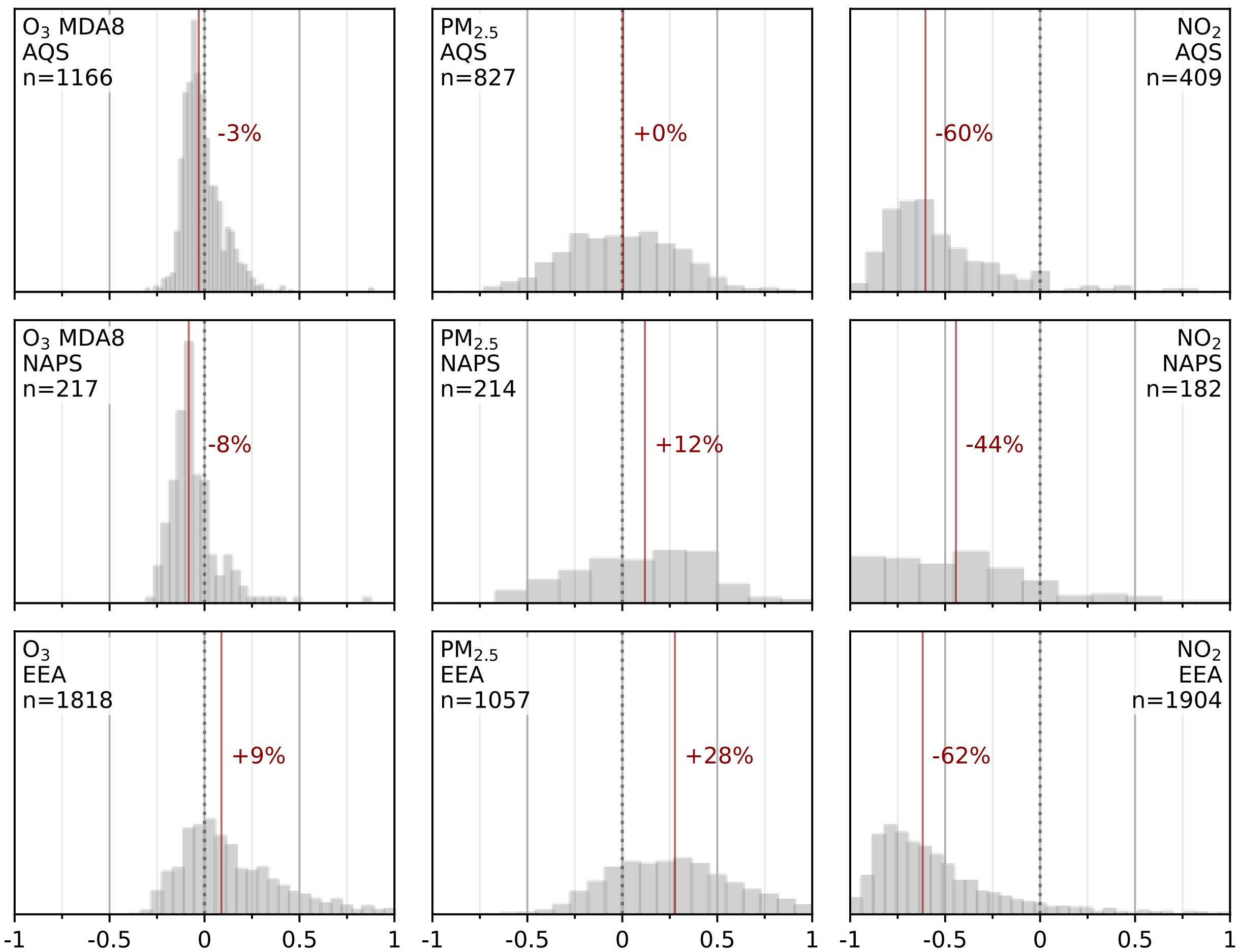

Supplementary Fig. 9: Histogram of relative difference between modeled and observed air quality metrics in Europe and North America. Values are 2019 annual averages. Each point is a measurement station. Medians are indicated in maroon. Datasets from AQS (US)[13], NAPS (Canada)[14], and EEA (Europe)[15].

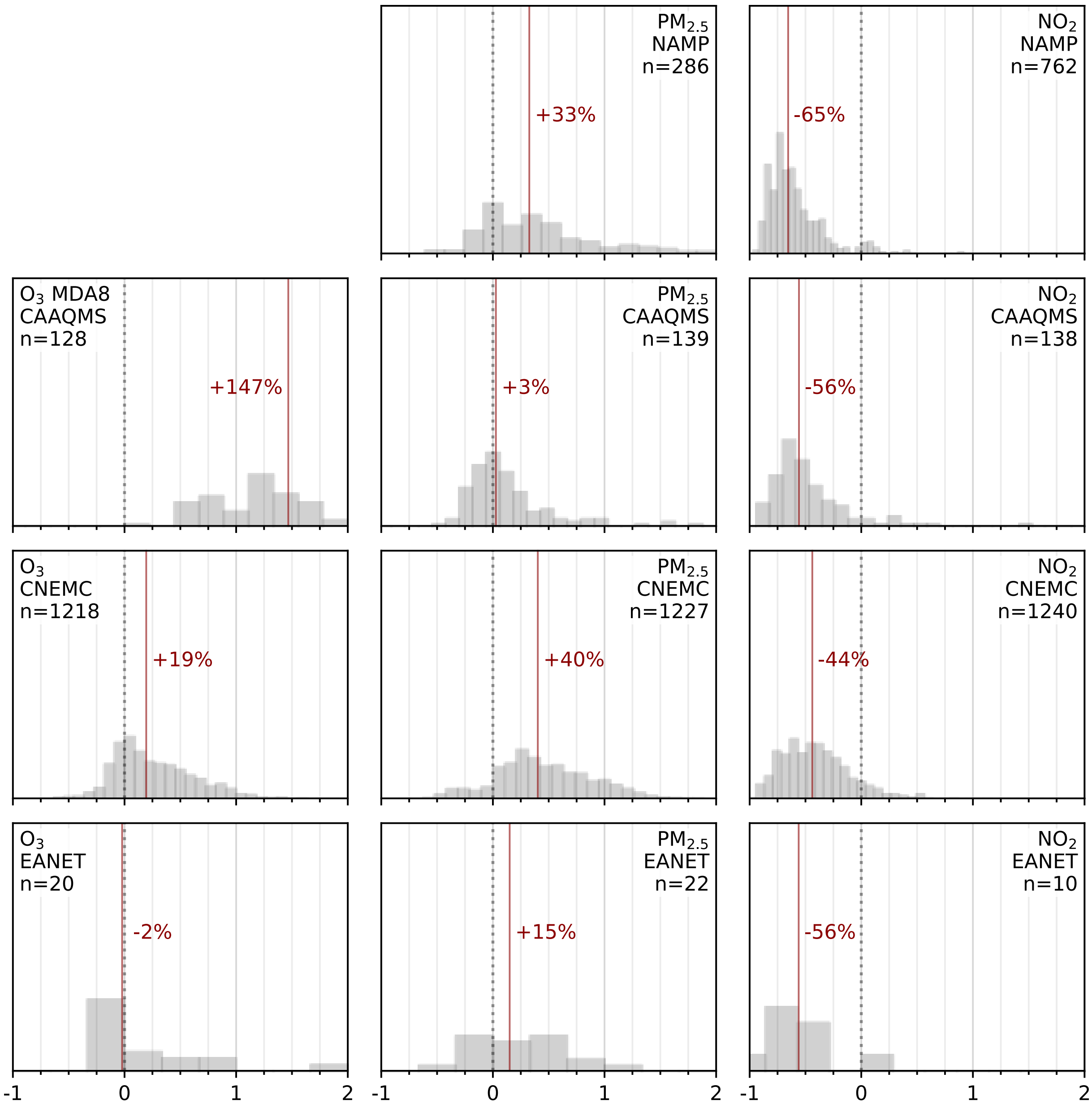

Supplementary Fig. 10: Histogram of relative difference between modeled and observed air quality metrics in Asia. Values are 2019 annual averages. Each point is a measurement station. Medians are indicated in maroon. Datasets from NAMP (India)[16], CAAQM (India)[17], CNEMC (China)[18], and EANET (East and Southeast Asia)[19].

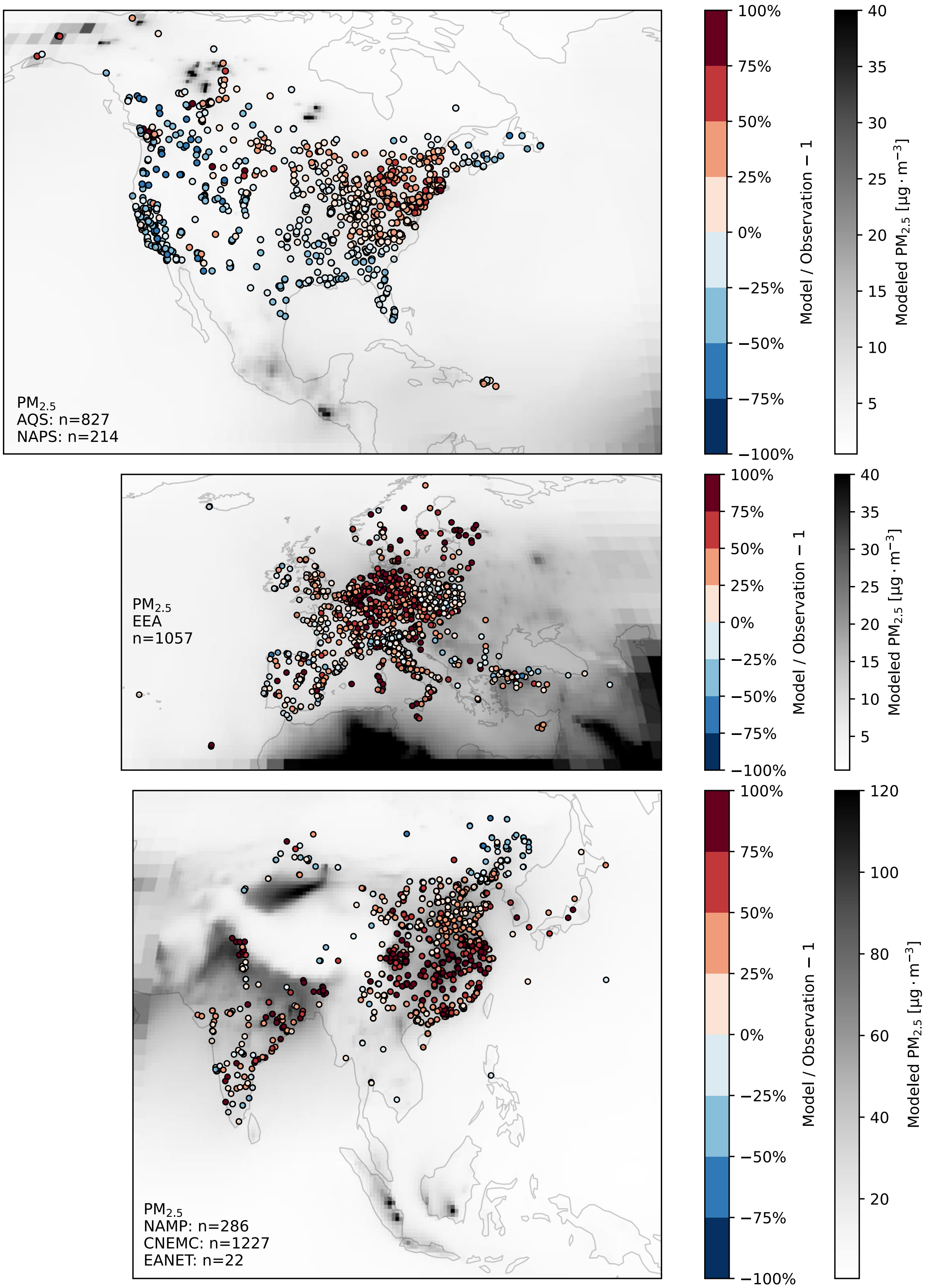

Supplementary Fig. 11 Spatial representation of relative difference between modeled and observed ground-level $PM_{2.5}$. Values are 2019 annual average mass concentrations. Markers indicate measurement stations.

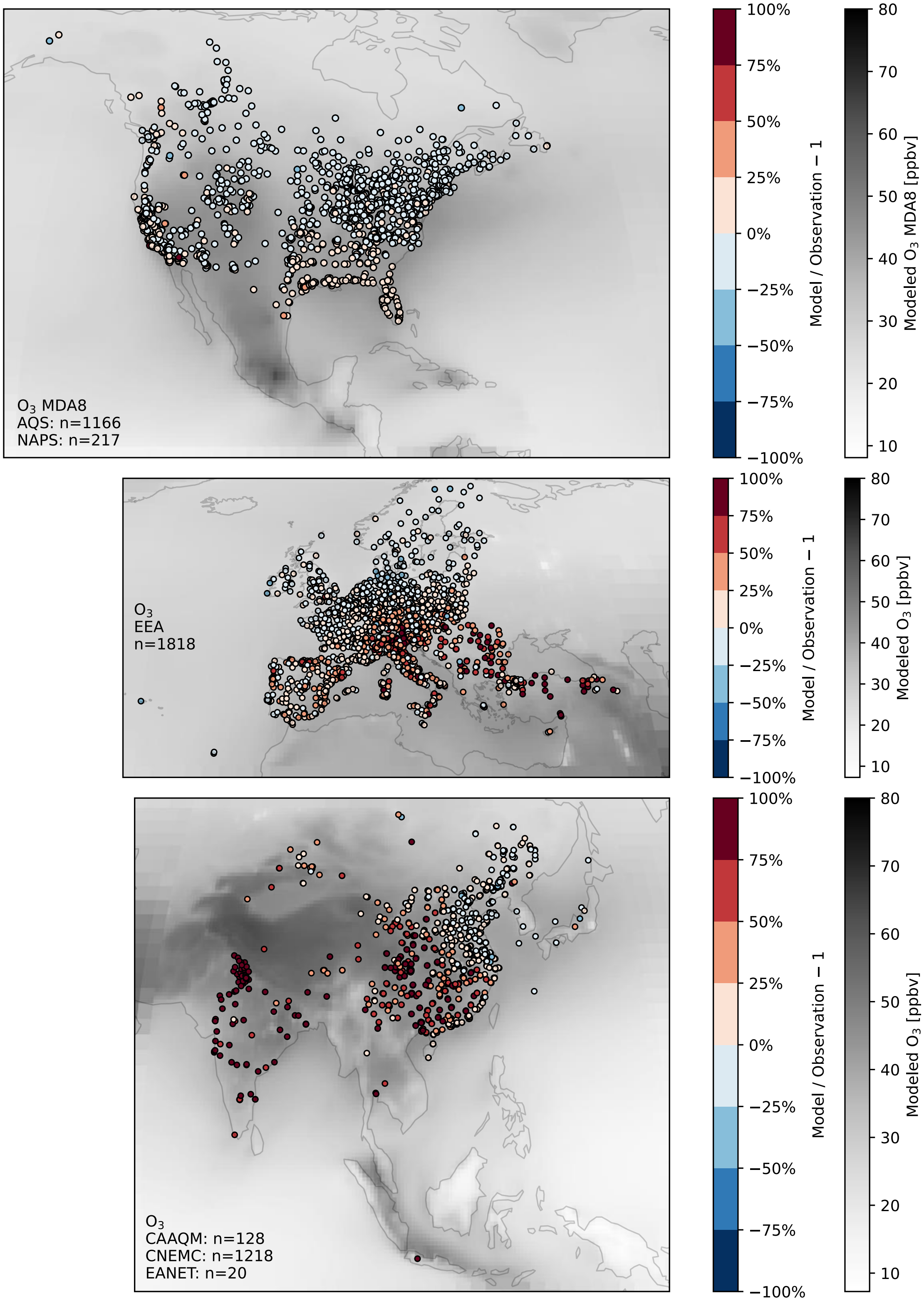

Supplementary Fig. 12: Spatial representation of relative difference between modeled and observed ground-level ozone. Values are 2019 annual average volume mixing ratios. Markers indicate measurement stations.

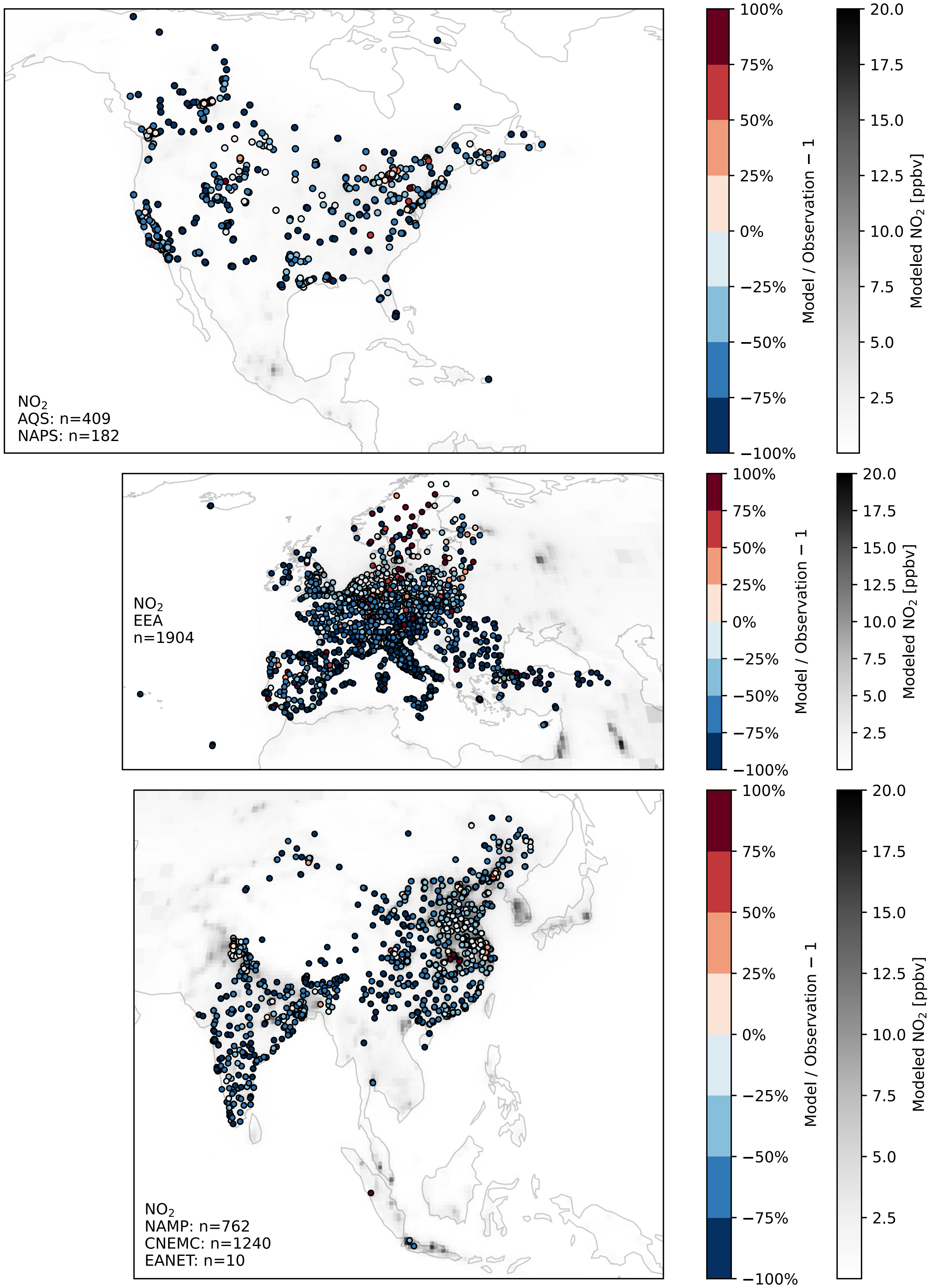

Supplementary Fig. 13: Spatial representation of relative difference between modeled and observed ground-level NO_2. Values are 2019 annual average volume mixing ratios. Markers indicate measurement stations.

Supplementary Table 5: Comparison of modeled (M) and observed (O) ground-level $PM_{2.5}$. Values are 2019 annual average mass concentrations in $\mu g \cdot m^{-3}$.

	U.S.	Canada	Europe
Measurement source	AQS[13]	NAPS[14]	EEA[15]
Number of stations	827	214	1057
Mean (M)	7.1	6.6	15
Mean (O)	7.3	6.1	12
Standard deviation (M)	2.4	2.2	4.3
Standard deviation (O)	1.9	1.5	5.1
Mean bias	−0.15	+0.48	+2.7
Mean error	1.8	1.8	3.9
RMS error	2.3	2.2	4.7
Normalized mean bias	−2%	+8%	+22%
Normalized mean error	24%	29%	31%
Correlation coefficient	0.47	0.41	0.68
Index of agreement	0.69	0.63	0.76

	India	India	China	East and Southeast Asia
Measurement source	NAMP[16]	CAAQM[17]	CNEMC[18]	EANET[19]
Number of stations	286	139	1227	22
Mean (M)	55	78	55	20
Mean (O)	41	75	39	18
Standard deviation (M)	23	25	19	8.7
Standard deviation (O)	24	31	13	10
Mean bias	+14	+3.2	+16	+2.0
Mean error	18	16	18	6.8
RMS error	24	21	22	9.1
Normalized mean bias	+34%	+4%	+42%	+12%
Normalized mean error	44%	21%	47%	38%
Correlation coefficient	0.65	0.75	0.65	0.55
Index of agreement	0.75	0.85	0.62	0.71

Supplementary Table 6: Comparison of modeled (M) and observed (O) ground-level ozone. Values are 2019 annual average volumetric mixing ratios in ppb.

	U.S.	Canada	Europe
Measurement source	AQS[13]	NAPS[14]	EEA[15]
Averaging	MDA8	MDA8	Daily
Number of stations	1166	217	1818
Mean (M)	41	31	31
Mean (O)	42	33	28
Standard deviation (M)	4.3	2.7	5.7
Standard deviation (O)	4.8	3.9	6.0
Mean bias	−0.67	−2.2	+3.5
Mean error	3.3	3.6	5.9
RMS error	4.1	4.3	7.8
Normalized mean bias	−2%	−7%	+12%
Normalized mean error	8%	11%	21%
Correlation coefficient	0.61	0.43	0.28
Index of agreement	0.77	0.60	0.56

	India	China	East and Southeast Asia
Measurement source	CAAQM[17]	CNEMC[18]	EANET[19]
Averaging	Daily	Daily	Daily
Number of stations	128	1218	20
Mean (M)	44	38	37
Mean (O)	18	31	34
Standard deviation (M)	4.5	6.8	4.1
Standard deviation (O)	6.0	5.3	12
Mean bias	+26	+6.8	+2.7
Mean error	26	8.9	9.5
RMS error	27	11	12
Normalized mean bias	+150%	+22%	+8%
Normalized mean error	150%	29%	28%
Correlation coefficient	0.07	−0.08	0.27
Index of agreement	0.26	0.36	0.43

Supplementary Table 7: Comparison of modeled (M) and observed (O) ground-level NO_2. Values are 2019 annual average volumetric mixing ratios in ppb.

	U.S.	Canada	Europe
Measurement source	AQS[13]	NAPS[14]	EEA[15]
Number of stations	409	182	1904
Mean (M)	3.2	3.7	3.1
Mean (O)	8.1	6.7	8.3
Standard deviation (M)	2.4	2.7	1.8
Standard deviation (O)	5.3	3.7	4.7
Mean bias	−4.9	−3.0	−5.2
Mean error	5.0	3.3	5.3
RMS error	6.6	4.2	6.8
Normalized mean bias	−60%	−45%	−63%
Normalized mean error	62%	49%	65%
Correlation coefficient	0.58	0.62	0.33
Index of agreement	0.57	0.65	0.48

	India	India	China	East and Southeast Asia
Measurement source	NAMP[16]	CAAQM[17]	CNEMC[18]	EANET[19]
Number of stations	762	138	1240	10
Mean (M)	4.6	8.0	9.5	7.5
Mean (O)	13	18	15	18
Standard deviation (M)	3.0	4.2	6.2	5.2
Standard deviation (O)	7.4	8.9	5.4	11
Mean bias	−8.2	−9.7	−5.8	−10
Mean error	8.3	10	6.5	11
RMS error	10	12	7.5	15
Normalized mean bias	−64%	−55%	−38%	−57%
Normalized mean error	65%	57%	42%	65%
Correlation coefficient	0.52	0.50	0.67	0.13
Index of agreement	0.51	0.52	0.67	0.46

Supplementary Tables 8 – 14

Supplementary Table 8: Modeled composition of aircraft-attributable $PM_{2.5}$. Share of individual components of modeled ground-level $PM_{2.5}$ attributed to aircraft emissions in 2019, per mass, for different perturbation scenarios. The values represent annual global area-weighted averages.

	Full-flight All-species	Full-flight NO_x-only	LTO-only All-species
Nitrate aerosol	52.4%	78.4%	11.8%
Sulfate aerosol	23.2%	2.5%	58.2%
Ammonium aerosol	19.3%	19.1%	20.7%
Organic aerosol	4.5%	0.0%	8.2%
Black carbon	0.6%	0.0%	1.1%

Supplementary Table 9: $PM_{2.5}$ health impacts in 2019 estimated with different CRFs. Global excess mortality attributed to aircraft emissions estimated with different concentration-response functions. Only central or nominal estimates are indicated. The CRF used in the main text is shown in bold and italics, and values in parentheses indicate the difference to it. NCD = non-communicable diseases. LRI = lower respiratory infections. HR = hazard ratio for a 10 $\mu g.m^{-3}$ increase in $PM_{2.5}$, with reported confidence interval in parentheses. * Hazard ratios reported by Crouse et al. and Strak et al. for 5 $\mu g.m^{-3}$ increments were converted to equivalent HRs for 10 $\mu g.m^{-3}$.

	CRF			Deaths, thousands	
Reference	End-point	HR	Notes	LTO	Full-flight
Burnett et al. (2018)[24]	***NCD + LRI***	***Non-linear, 1.096 at 10 $\mu g.m^{-3}$***	***Ensemble model, Table S2 in ref.***	***4.2***	***33.9***
Chen and Hoek (2020)[25]	All-Cause	1.08 (1.06–1.09)	Meta-analysis, Table 2 in ref.	4.6 (+8%)	40.0 (+18%)
Chen and Hoek (2020)[25]	Circulatory	1.11 (1.09–1.14)	Meta-analysis, Table 2 in ref.	2.4 (−44%)	21.7 (−36%)
Crouse et al. (2015)[26]	All-cause	1.022* (1.003–1.040)	CanCHEC cohort, multi-pollutant model, Table 1 in ref.	1.3 (−69%)	11.4 (−66%)
Crouse et al. (2015)[26]	All-cause	1.071* (1.059–1.084)	CanCHEC cohort, single-pollutant model, Table 1 in ref.	4.1 (−3%)	35.7 (+5%)
Hoek et al. (2013)[27]	Circulatory	1.11 (1.05–1.16)	Meta-analysis, Fig. 2 in ref.	2.4 (−44%)	21.7 (−36%)
Jerrett et al. (2017)[28]	Circulatory	1.14 (1.11–1.17)	ACS CPS-II cohort, Table 4 in ref.	3.0 (−30%)	27.3 (−19%)
Pope et al. (2019)[29]	All-cause	1.12 (1.08–1.15)	NHIS cohort, Table 2 in ref.	6.8 (+59%)	58.8 (+74%)
Strak et al. (2021)[30]	All-cause	1.277* (1.223–1.334)	ELAPSE cohort, Table 2 in ref.	14.6 (+243%)	126.7 (+274%)
Turner et al. (2016)[31]	All-cause	1.04 (1.02–1.06)	ACS CPS-II cohort, COX multi-pollutant model, Table 2 in ref.	2.3 (−45%)	20.4 (−40%)
Turner et al. (2016)[31]	All-cause	1.06 (1.04–1.08)	ACS CPS-II cohort, COX single pollutant model, Table E10 in ref.	3.5 (−18%)	30.3 (−11%)
Vodonos et al. (2018)[32]	All-cause	Non-linear, 1.137 at 10 $\mu g.m^{-3}$	Meta-analysis, penalized spline model, Fig. 2 in ref.	5.7 (+33%)	44.8 (+32%)
Wu et al. (2020)[33]	All-cause	1.066 (1.058–1.074)	Medicare cohort, Table S3 in ref.	3.8 (−10%)	33.2 (−2%)

Supplementary Table 10: Ozone health impacts in 2019 estimated with different CRFs. Global excess mortality attributed to aircraft emissions estimated with different concentration-response functions. Only central or nominal estimates are indicated. The CRF used in the main text is shown in bold and italics, and values in parentheses indicate the difference to it. HR = hazard ratio for a 10 ppbv increase in ozone, with reported confidence interval in parentheses. MDA8/1 = maximum daily 8/1-hour average. * Hazard ratios reported by Huangfu and Atkinson and Kasdagli et al. for 10 µg.m^{-3}, Crouse et al. for 9.5 ppbv and Brauer et al. for 1 ppbv increments were converted to equivalent HRs for 10 ppbv.

	CRF				Deaths, thousands	
Reference	Metric	End-point	HR	Notes	LTO	Full-flight
Brauer et al. (2024)[34]	***Peak-season MDA8***	***COPD***	***1.074* (1.046–1.104)***	***Meta-analysis, p. 79 of appendix 1 in ref.***	***0.8***	***24.6***
Cakmak et al. (2018)[35]	Peak-season MDA8	All-cause	1.05 (0.99–1.11)	CanCHEC cohort, multi-pollutant model, Fig. 3 in ref.	7.9 (+865%)	194.9 (+692%)
Crouse et al. (2015)[26]	Peak-season MDA8	All-cause	1.019* (1.011–1.027)	CanCHEC cohort, multi-pollutant model, Table 1 in ref.	3.0 (+271%)	75.1 (+205%)
Crouse et al. (2015)[26]	Peak-season MDA8	All-cause	1.033* (1.027–1.038)	CanCHEC cohort, single-pollutant model, Table 1 in ref.	5.2 (+536%)	128.5 (+422%)
Di et al. (2017)[36]	Annual mean	All-cause	1.011 (1.010–1.012)	Medicare cohort, Table 2 in ref.	0.2 (−78%)	39.0 (+58%)
Huangfu and Atkinson (2020)[37]	Peak-season MDA8	All-cause	1.020* (1.000–1.040)	Meta-analysis, Fig. 8 in ref.	3.2 (+293%)	79.4 (+223%)
Huangfu and Atkinson (2020)[37]	Peak-season MDA8	Respiratory	1.040* (0.980–1.102)	Meta-analysis, Fig. 9 in ref.	0.8 (−4%)	20.9 (−15%)
Kasdagli et al. (2024)[38]	Annual mean	Respiratory	1.102* (1.040–1.166)	Meta-analysis, Table 2 in ref.	1.1 (+39%)	51.1 (+108%)
Kasdagli et al. (2024)[38]	Peak-season MDA8	All-cause	1.020* (0.941–1.081)	Meta-analysis, Table 2 in ref.	3.2 (+293%)	79.5 (+223%)
Kasdagli et al. (2024)[38]	Peak-season MDA8	Respiratory	1.020* (0.960–1.061	Meta-analysis, Table 2 in ref.	0.4 (−52%)	10.5 (−57%)
Jerrett et al. (2009)[39]	Peak-season MDA1	Respiratory	1.040 (1.013–1.067)	ACS CPS-II cohort, COX multi-pollutant model, Table 3 in ref.	0.9 (+13%)	21.3 (−13%)
Murray et al. (2020)[40]	Peak-season MDA8	COPD	1.063 (1.029–1.098)	Meta-analysis, p. 134 of appendix 1 in ref.	0.7 (−14%)	21.1 (−14%)
Turner et al. (2016)[31]	Annual mean MDA8	All-cause	1.02 (1.01–1.04)	ACS CPS-II cohort, COX multi-pollutant model, Table 2 in ref.	1.9 (+128%)	80.0 (+225%)
Turner et al. (2016)[31]	Annual mean MDA8	COPD	1.14 (1.08–1.21)	ACS CPS-II cohort, COX multi-pollutant model, Table 2 in ref.	0.9 (+8%)	44.4 (+81%)
Turner et al. (2016)[31]	Annual mean MDA8	Respiratory	1.12 (1.08–1.16)	ACS CPS-II cohort, COX multi-pollutant model, Table 2 in ref.	1.3 (+62%)	59.4 (+142%)
Turner et al. (2016)[31]	Peak-season MDA8	Respiratory	1.08 (1.06–1.11)	ACS CPS-II cohort, COX multi-pollutant model, Table E9 in ref.	1.5 (+86%)	40.6 (+65%)

Supplementary Table 11: NO_2 health impacts in 2019 estimated with different CRFs. Global excess mortality attributed to aircraft emissions estimated with different concentration-response functions. Only central or nominal estimates are indicated. The CRF used in the main text is shown in bold, and values in parentheses indicate the difference to it. HR = hazard ratio for a 10 ppbv increase in NO_2, with reported confidence interval in parentheses. * Hazard ratios reported by Kasdagli e al., Huangfu and Atkinson, and Strak et al. for 10 µg.m^{-3}, Crouse et al. for 8.1 ppbv increments were converted to equivalent HRs for 10 ppbv.

CRF				Deaths, thousands	
Reference	End-point	HR	Notes	LTO	Full-flight
Kasdagli et al. (2024)[38]	***All-cause***	***1.098* (1.058–1.138)***	***Meta-analysis, Table 1 in ref.***	***8.7***	***6.7***
Crouse et al. (2015)[26]	All-cause	1.056* (1.046–1.065)	CanCHEC cohort, multi-pollutant model, Table 1 in ref.	5.1 (−42%)	3.9 (−42%)
Crouse et al. (2015)[26]	All-cause	1.065* (1.056–1.073)	CanCHEC cohort, single-pollutant model, Table 1 in ref.	5.8 (−33%)	4.5 (−33%)
Huangfu and Atkinson (2020)[37]	All-cause	1.039* (1.019–1.078)	Meta-analysis, Fig. 2	3.5 (−59%)	2.7 (−59%)
Huangfu and Atkinson (2020)[37]	Respiratory	1.058* (1.019–1.098)	Meta-analysis, Fig. 3	0.7 (−92%)	0.5 (−93%)
Huangfu and Atkinson (2020)[37]	COPD	1.058* (1.019–1.078)	Meta-analysis, Fig. 4	0.4 (−95%)	0.3 (−96%)
Kasdagli et al. (2024)[38]	Circulatory	1.098* (1.058–1.159)	Meta-analysis, Table 1 in ref.	3.1 (−64%)	2.5 (−63%)
Kasdagli et al. (2024)[38]	Respiratory	1.098* (1.058–1.138)	Meta-analysis, Table 1 in ref.	1.1 (−87%)	0.8 (−88%)
Kasdagli et al. (2024)[38]	COPD	1.078* (1.039–1.118)	Meta-analysis, Table 1 in ref.	0.5 (−94%)	0.3 (−95%)
Strak et al. (2021)[30]	All-cause	1.171* (1.138–1.204)	ELAPSE cohort, Table 2 in ref.	14.7 (+69%)	11.4 (+69%)
Turner et al. (2016)[31]	All-cause	1.01 (1.00–1.03)	ACS CPS-II cohort, COX multi-pollutant model, Table 2 in ref.	0.9 (−89%)	0.7 (−89%)

Supplementary Table 12: Annual global aircraft-attributable premature mortality with and without non-NO_x emissions. Negative values indicate higher mortality when aircraft emissions are excluded. 2019 results consider only the same 2.0° × 2.5° resolution as the 2040 simulations, excluding nested domain simulations at finer resolution. HC = hydrocarbons, nvPM = nonvolatile particulate matter.

Population and emissions	Aircraft emissions					Early deaths (×10^3)		
	NO_x	SO_x	HC	CO	nvPM	$PM_{2.5}$	Ozone	NO_2
2019	✓	✓	✓	✓	✓	31.1	21.8	−1.9
	✓	—	—	—	—	26.8 (−14%)	22.0 (+0.8%)	−1.9 (−0.3%)
2040 SSP2-4.5	✓	✓	✓	✓	✓	68.0	54.1	−0.9
	✓	—	—	—	—	58.7 (−14%)	54.2 (+0.1%)	-0.8 (−5.6%)

Supplementary Table 13: Health impacts of aircraft emissions in 2040 estimated with baseline mortality rates from 2019. Global excess mortality attributed to full-flight (FF) or LTO-only emissions, estimated using baseline mortality rates from GHE 2019[41]. The percentages in parentheses indicate the difference to the estimates using baseline mortality rates projected to 2040 by the International Futures (IFs) model[42], as used in the main text.

Population and non-aviation emissions	Aircraft emissions	Early deaths (×10³)					
		$PM_{2.5}$		Ozone		NO_2	
		GHE 2019	IFs 2040	GHE 2019	IFs 2040	GHE 2019	IFs 2040
SSP1-1.9	FF	52.3 (−30%)	74.4	38.4 (−38%)	61.9	0.5 (−79%)	2.3
SSP1-2.6	FF	58.4 (−29%)	82.7	35.5 (−38%)	56.9	1.6 (−67%)	4.8
SSP2-4.5	FF	53.6 (−21%)	68.0	37.1 (−32%)	54.1	−2.3 (+165%)	−0.9
SSP3-7.0	FF	50.4 (−25%)	66.8	36.8 (−31%)	53.0	3.2 (−58%)	7.6
SSP4-3.4	FF	52.1 (−18%)	63.8	36.7 (−30%)	52.7	−3.2 (+44%)	−2.2
SSP4-6.0	FF	53.6 (−18%)	65.6	35.8 (−30%)	51.2	0.8 (−79%)	3.7
SSP5-8.5	FF	70.8 (−28%)	97.9	33.4 (−37%)	53.4	4.8 (−44%)	8.5
SSP1-1.9	LTO	12.2 (−32%)	18.0	3.6 (−41%)	6.1	7.4 (−31%)	10.7
SSP2-4.5	LTO	8.6 (−25%)	11.5	2.4 (−33%)	3.6	6.8 (−23%)	8.9
SSP3-7.0	LTO	12.8 (−26%)	17.4	1.6 (−28%)	2.3	6.2 (−26%)	8.4

Supplementary Table 14: Health impacts of aircraft emissions in SSP2-4.5 2040 estimated with different projections of baseline mortality rate. Global excess mortality attributed to full-flight (FF) and LTO-only emissions, estimated using baseline mortality rate projections for 2040 from GBD Foresight[43,44]. The percentages in parentheses indicate the difference to the estimates using baseline mortality rates projected to 2040 by the International Futures (IFs) model, as used in the main text, which are shown in bold and italic. Unlike the IFs projections, those from GBD Foresight are independent of SSP, which is why only results for SSP2-4.5 are shown here. In addition to a "reference" scenario, GBD Foresight has three scenarios that consider additional health improvements, and a scenario that considers all three types of improvements combined.

GBD Foresight scenario[43,44]	Early deaths (×10³)					
	$PM_{2.5}$		Ozone		NO_2	
	FF	LTO	FF	LTO	FF	LTO
(IFs)	***68.0***	***11.5***	***54.1***	***3.6***	***−0.9***	***8.9***
Reference	68.7 (+1%)	11.4 (0%)	48.1 (−11%)	3.5 (−5%)	−0.5 (−48%)	8.9 (0%)
Safer Environment	67.7 (0%)	11.3 (−1%)	46.0 (−15%)	3.4 (−8%)	−0.3 (−66%)	8.8 (−2%)
Improved Behavioral and Metabolic Risks	54.2 (−20%)	9.2 (−19%)	46.8 (−13%)	3.4 (−7%)	0.1 (−112%)	7.3 (−18%)
Improved Childhood Nutrition and Vaccination	68.7 (+1%)	11.4 (−1%)	48.1 (−11%)	3.5 (−5%)	−0.5 (−47%)	8.9 (0%)
Combined	53.4 (−21%)	9.2 (−20%)	44.7 (−17%)	3.3 (−10%)	0.2 (−127%)	7.2 (−19%)

Supplementary Figures 14 – 29

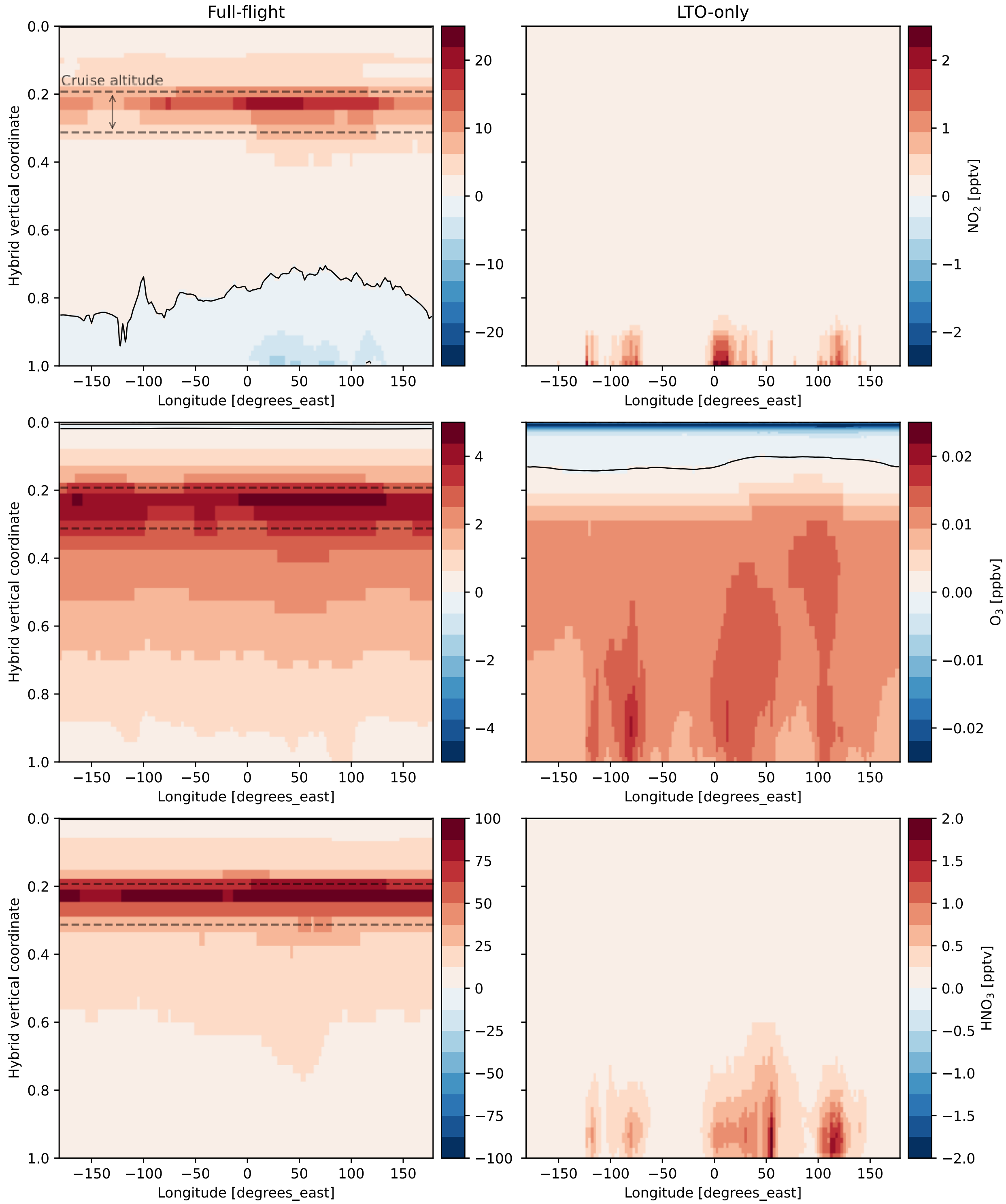

Supplementary Fig. 14: Longitudinal means of aircraft-attributable concentration changes related to NO_x in 2019. Annual average changes in concentration of NO_2 (top), O_3 (middle) and HNO_3 (bottom). Impacts attributed to full-flight (left), and LTO-only emissions (right), using smaller scales on the LTO-only plots. Thin solid black lines highlight changes of sign. Horizontal dashed lines labeled as "cruise altitude" indicate the altitude range where roughly 70% of full-flight aircraft NO_x emissions are released.

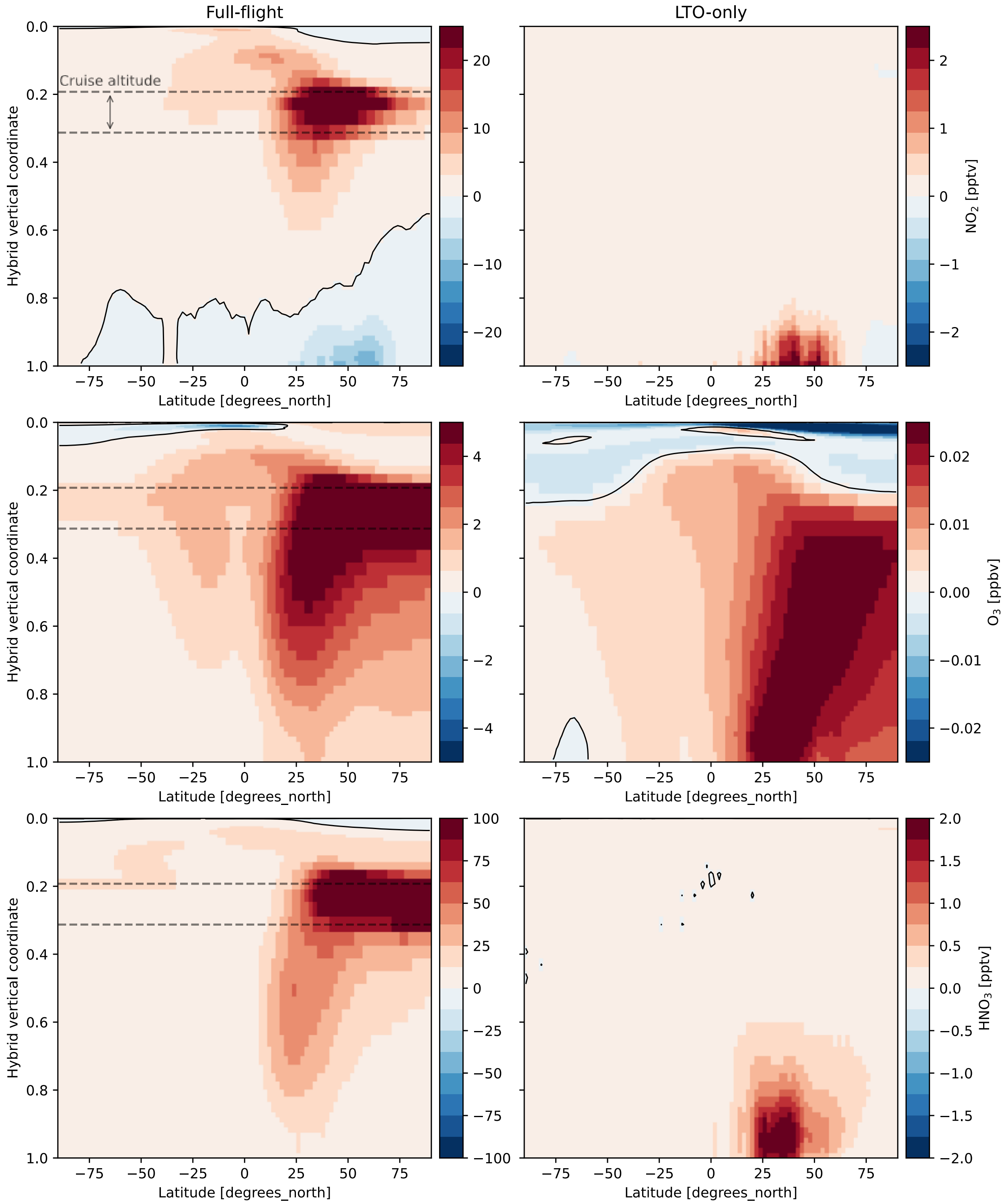

Supplementary Fig. 15: Zonal means of aircraft-attributable concentration changes related to NO_x in 2019. Annual average changes in concentration of NO_2 (top), O_3 (middle) and HNO_3 (bottom). Impacts attributed to full-flight (left), and LTO-only emissions (right), using smaller scales on the LTO-only plots. Thin solid black lines highlight changes of sign. Horizontal dashed lines labeled as "cruise altitude" indicate the altitude range where roughly 70% of full-flight aircraft NO_x emissions are released.

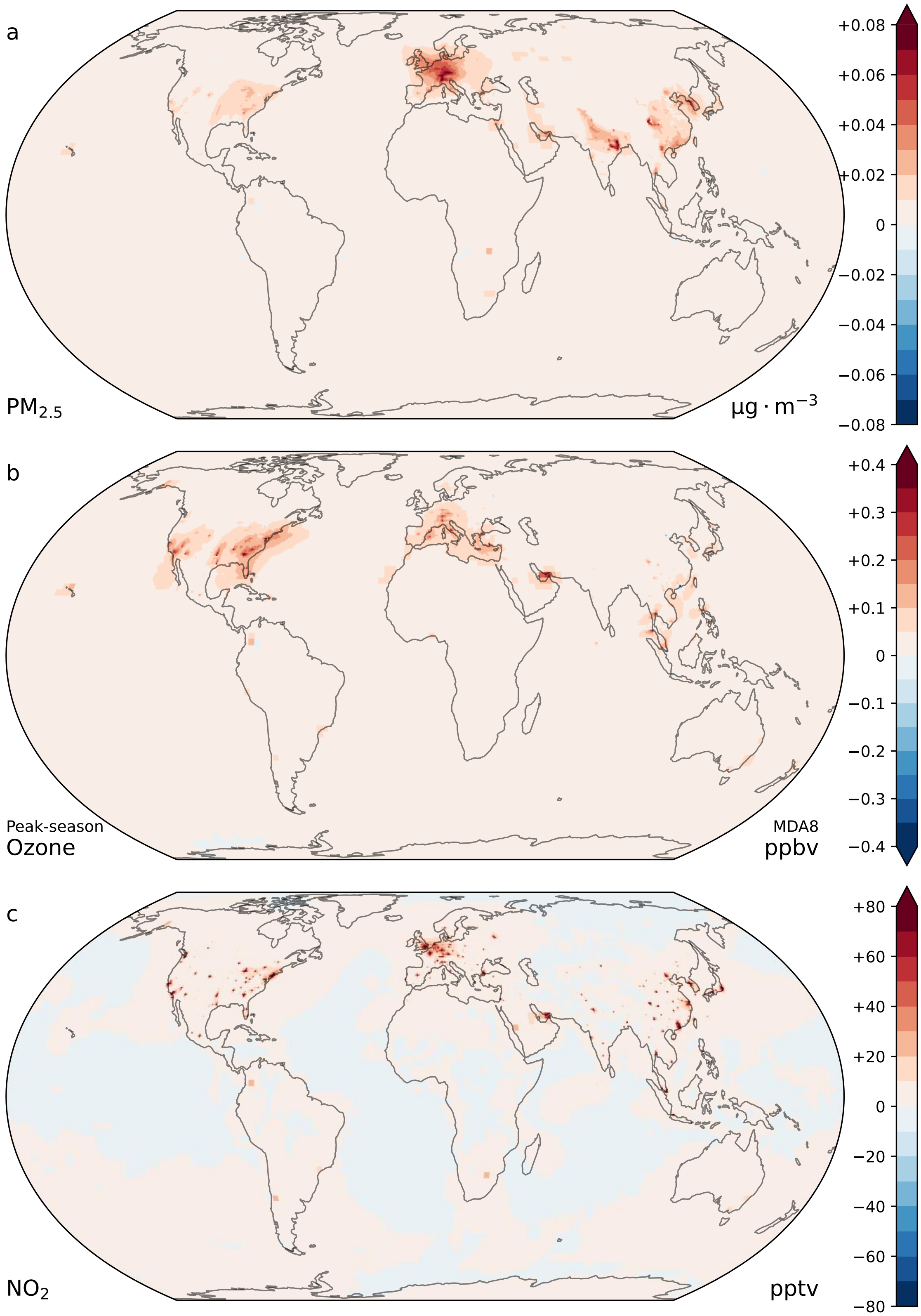

Supplementary Fig. 16: Ground-level changes in 2019 attributed to LTO emissions. a, $PM_{2.5}$ mass concentration. **b**, peak-season ozone mixing ratio MDA8. **c**, NO_2 mixing ratio.

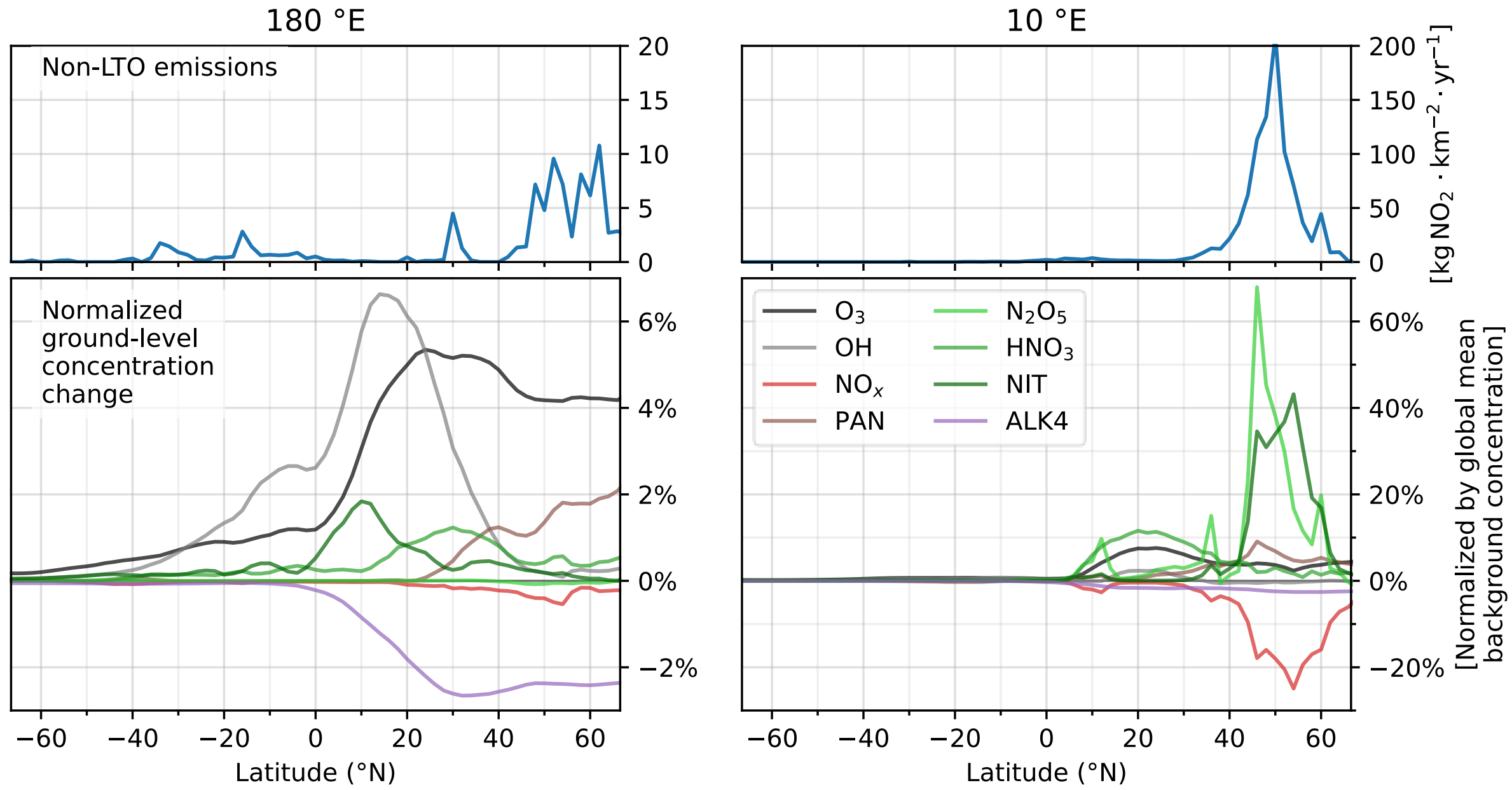

Supplementary Fig. 17: Ground-level annual mean concentration changes due to non-LTO emissions at two meridians in 2019. Top panels indicate NO_x emission rates summed vertically. Bottom panels present concentration values taken as a difference between a simulation with full-flight aircraft emissions and one with only LTO emissions, normalized by the global mean background surface concentration of the species. Note different unit scales between left panels at a longitude with low aircraft emissions (180 °E) and right panels at a longitude with high emissions (10 °E). PAN = peroxyacyl nitrates, NIT = inorganic nitrates, ALK4 = lumped C4+ alkanes.

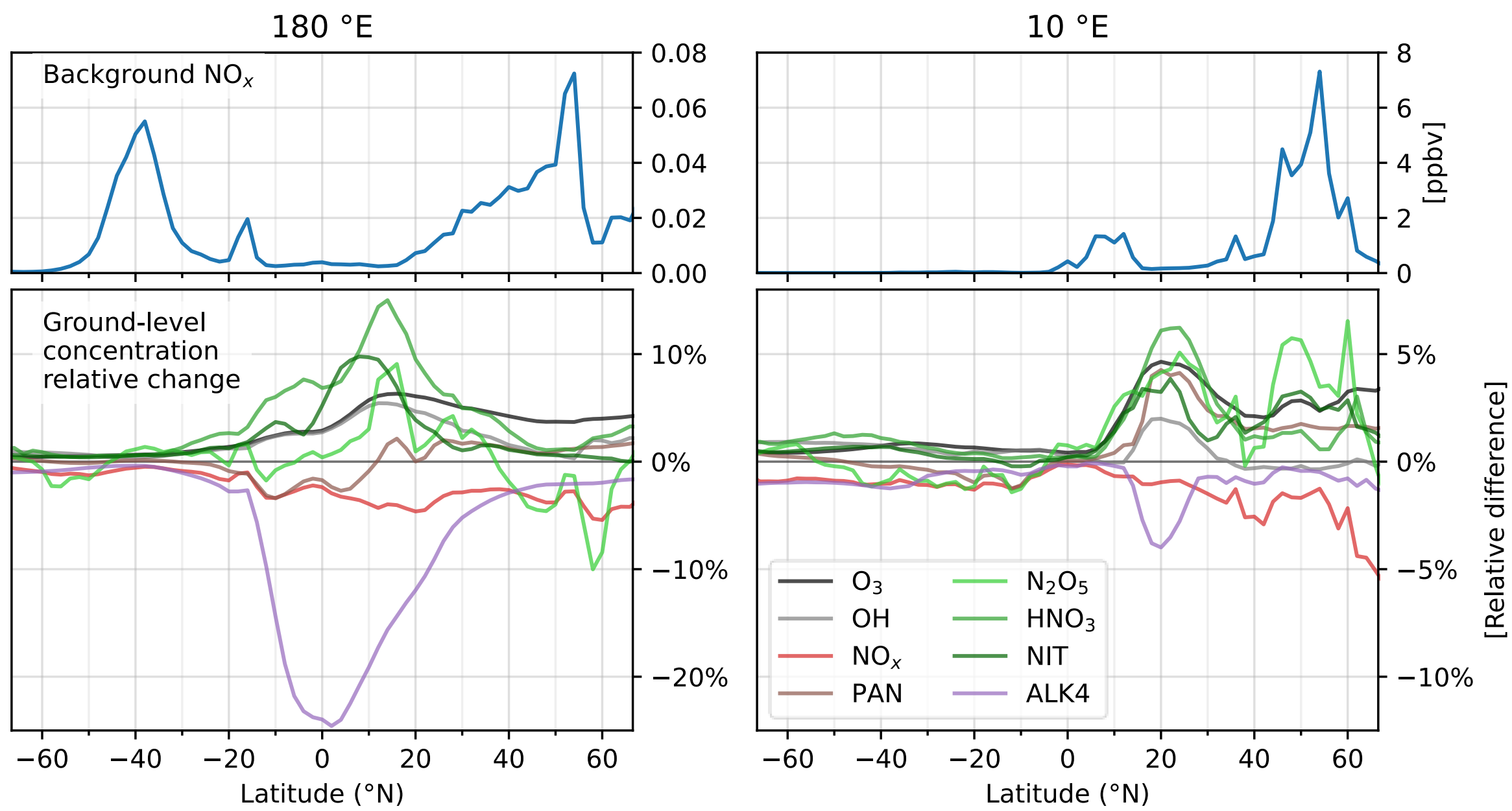

Supplementary Fig. 18: Ground-level annual mean concentration relative changes due to non-LTO emissions at two meridians in 2019. Top panels indicate background NO_x concentrations. Bottom panels present concentration values taken as a difference between a simulation with full-flight aircraft emissions and one with only LTO emissions, relative to the local background surface concentration of the species. Note different unit scales between left panels at a longitude with low NO_x concentrations (180 °E) and right panels at a longitude with high concentrations (10 °E). PAN = peroxyacyl nitrates, NIT = inorganic nitrates, ALK4 = lumped C4+ alkanes.

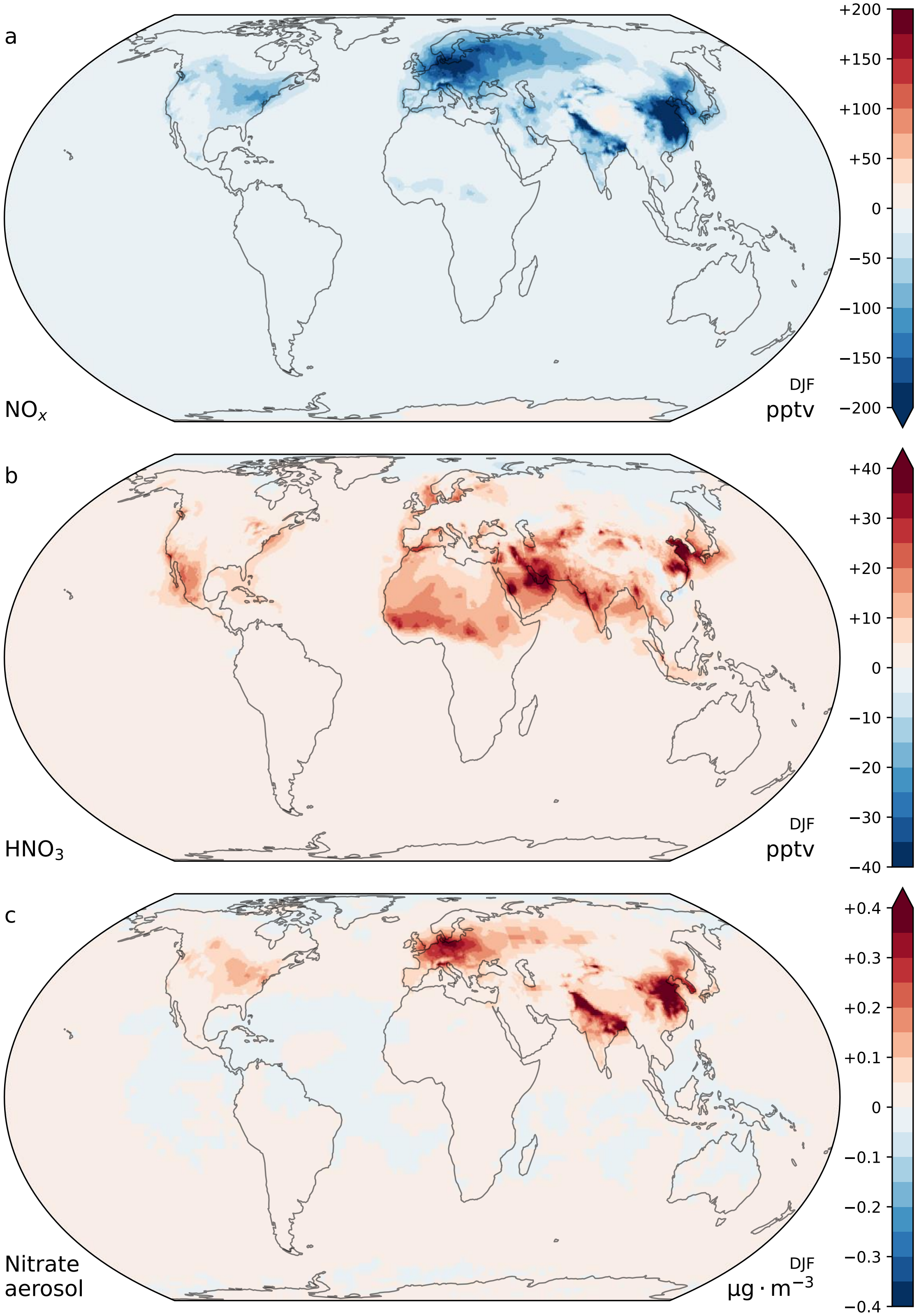

Supplementary Fig. 19: Boreal winter ground-level changes attributed to non-LTO emissions. a, NO_x mixing ratio. **b**, HNO_3 mixing ratio. **c**, Inorganic nitrate aerosol mass concentration. All values averaged over December, January, and February 2019 (DJF).

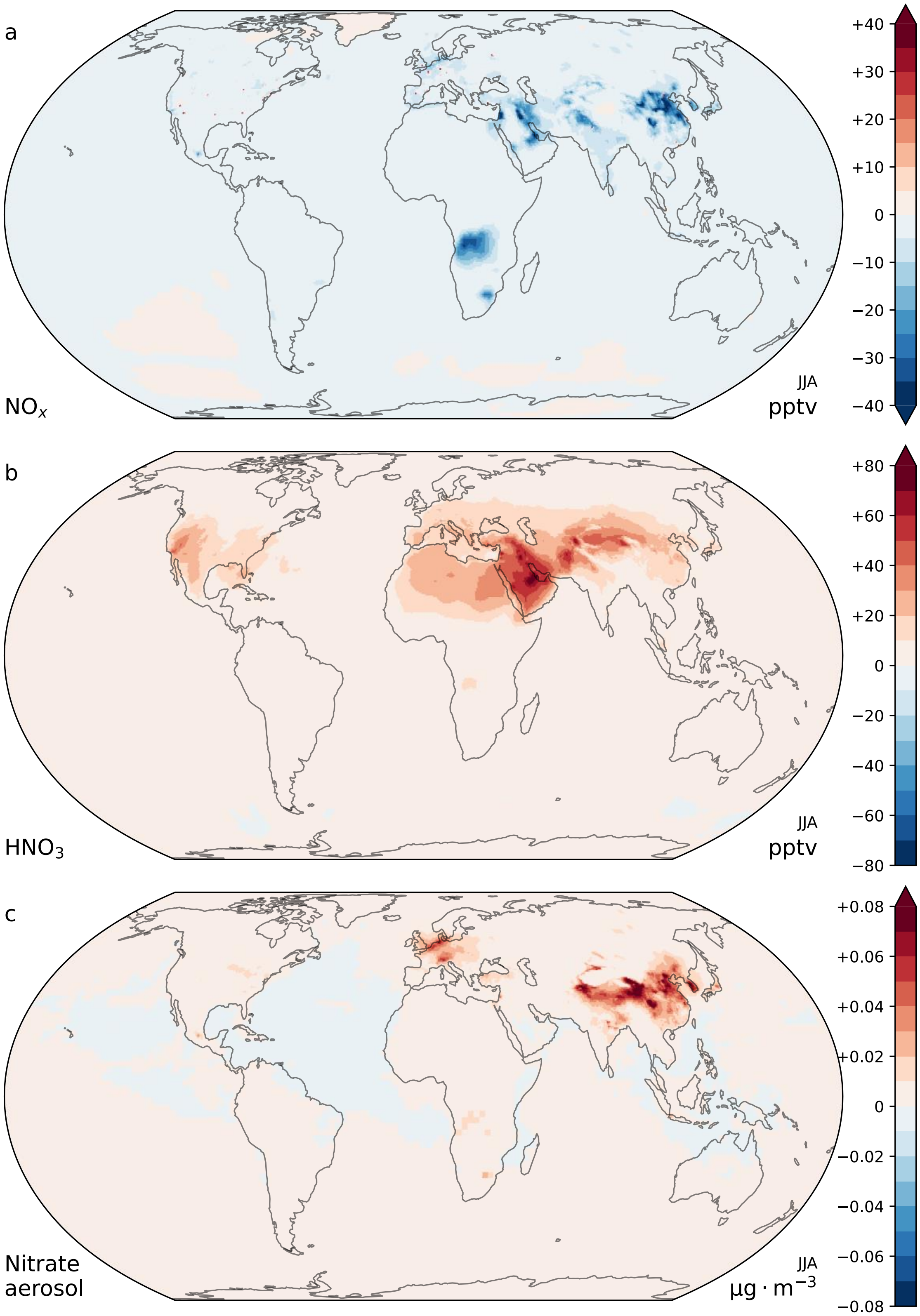

Supplementary Fig. 20: Boreal summer ground-level changes attributed to non-LTO emissions. a, NO_x mixing ratio. **b**, HNO_3 mixing ratio. **c**, Inorganic nitrate aerosol mass concentration. All values averaged over June, July, and August 2019 (JJA).

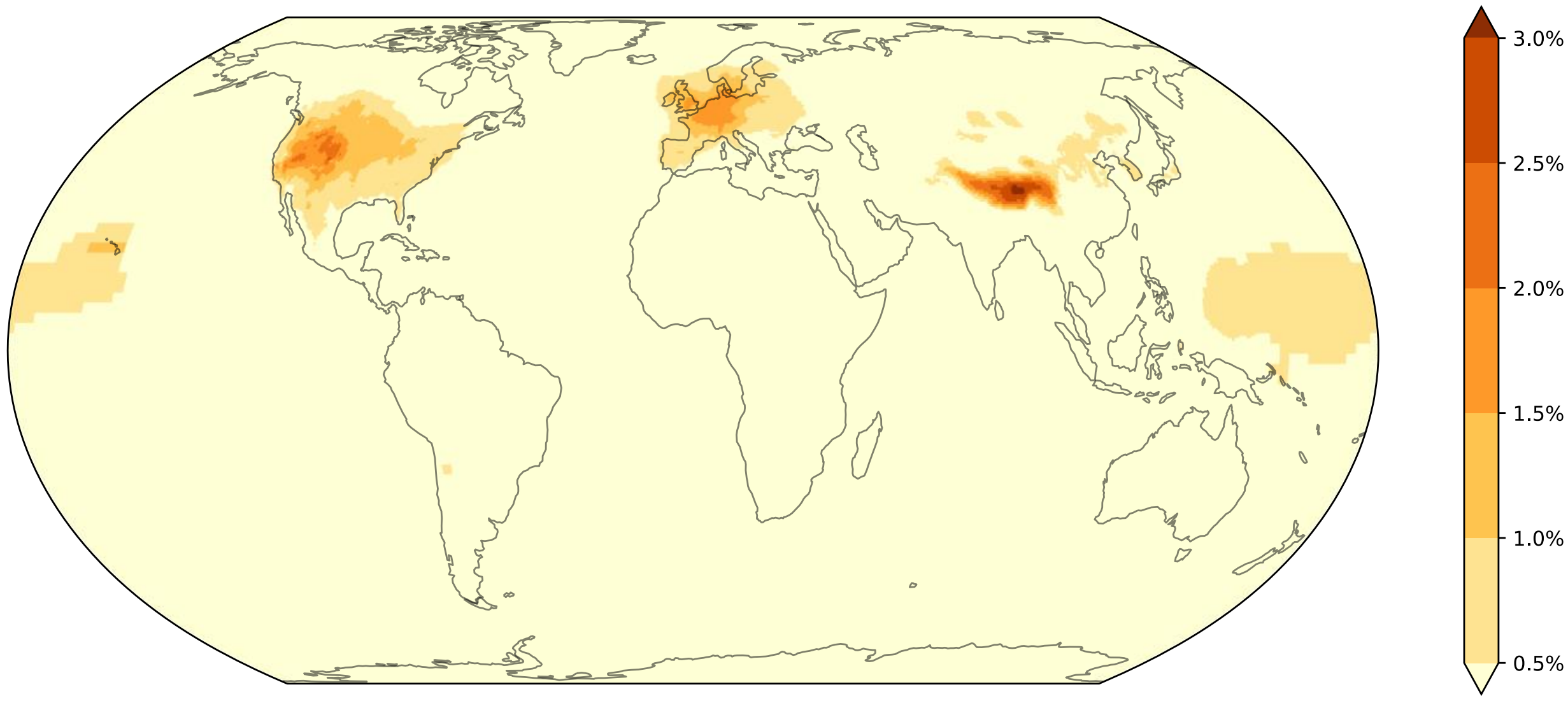

Supplementary Fig. 21: Contribution of aircraft to ground-level $PM_{2.5}$. Relative change in 2019 average concentrations attributed to full-flight emissions.

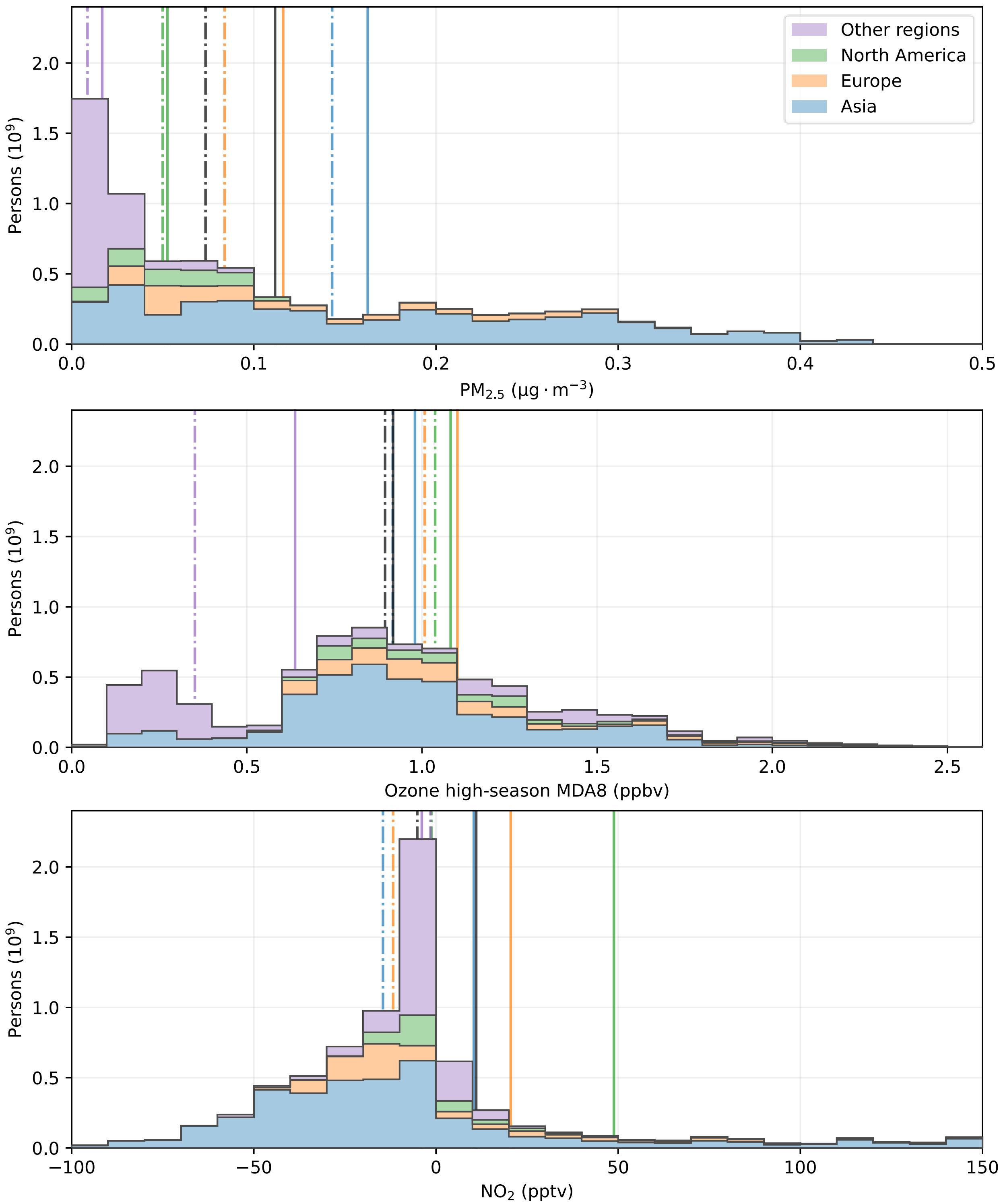

Supplementary Fig. 22: Population size subjected to different levels of aircraft-attributable pollution. Histogram of population exposure attributed to 2019 full-flight emissions, per region. Vertical lines indicate mean (solid lines) and median (dash-dotted lines) population exposure for each area, and globally (black lines).

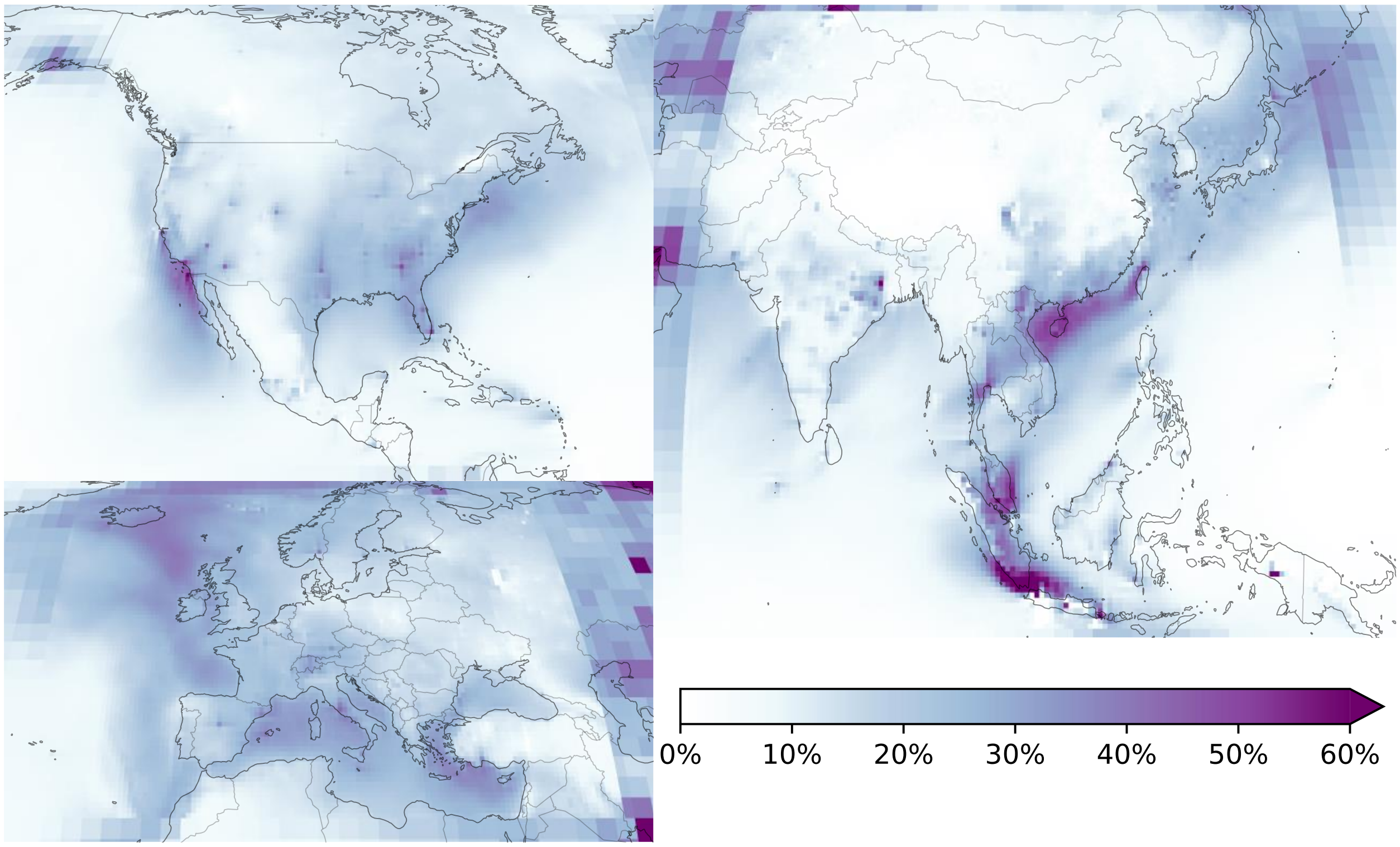

Supplementary Fig. 23: Contribution of LTO emissions to aircraft-attributable $PM_{2.5}$. Ratio between 2019 average ground-level concentration attributed to LTO and full-flight emissions over North America, Europe, and Asia.

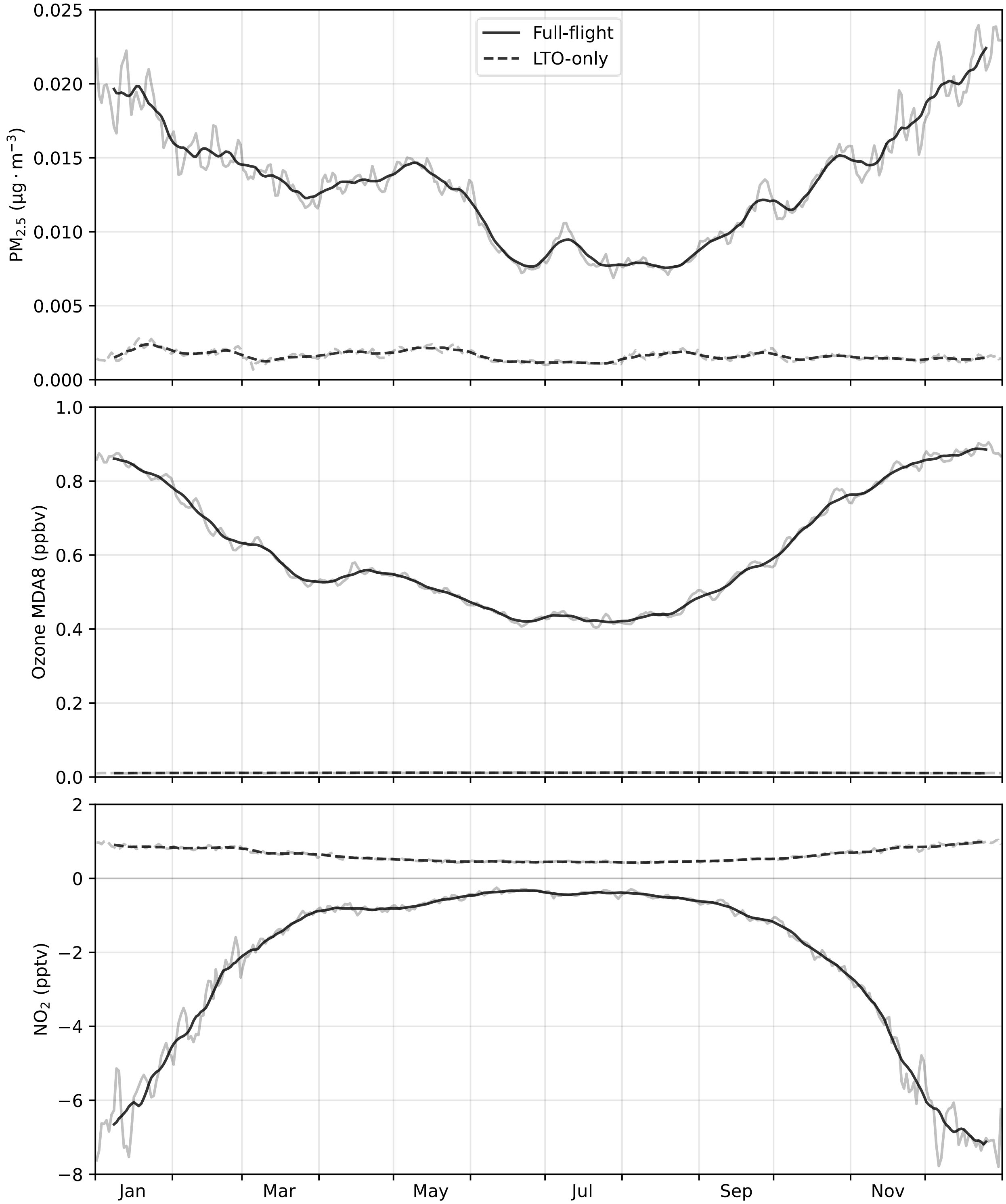

Supplementary Fig. 24: Seasonality of aircraft-attributable ground-level pollution. Daily and 14-day area-weighted global average concentration changes attributed to 2019 full-flight and LTO-only emissions.

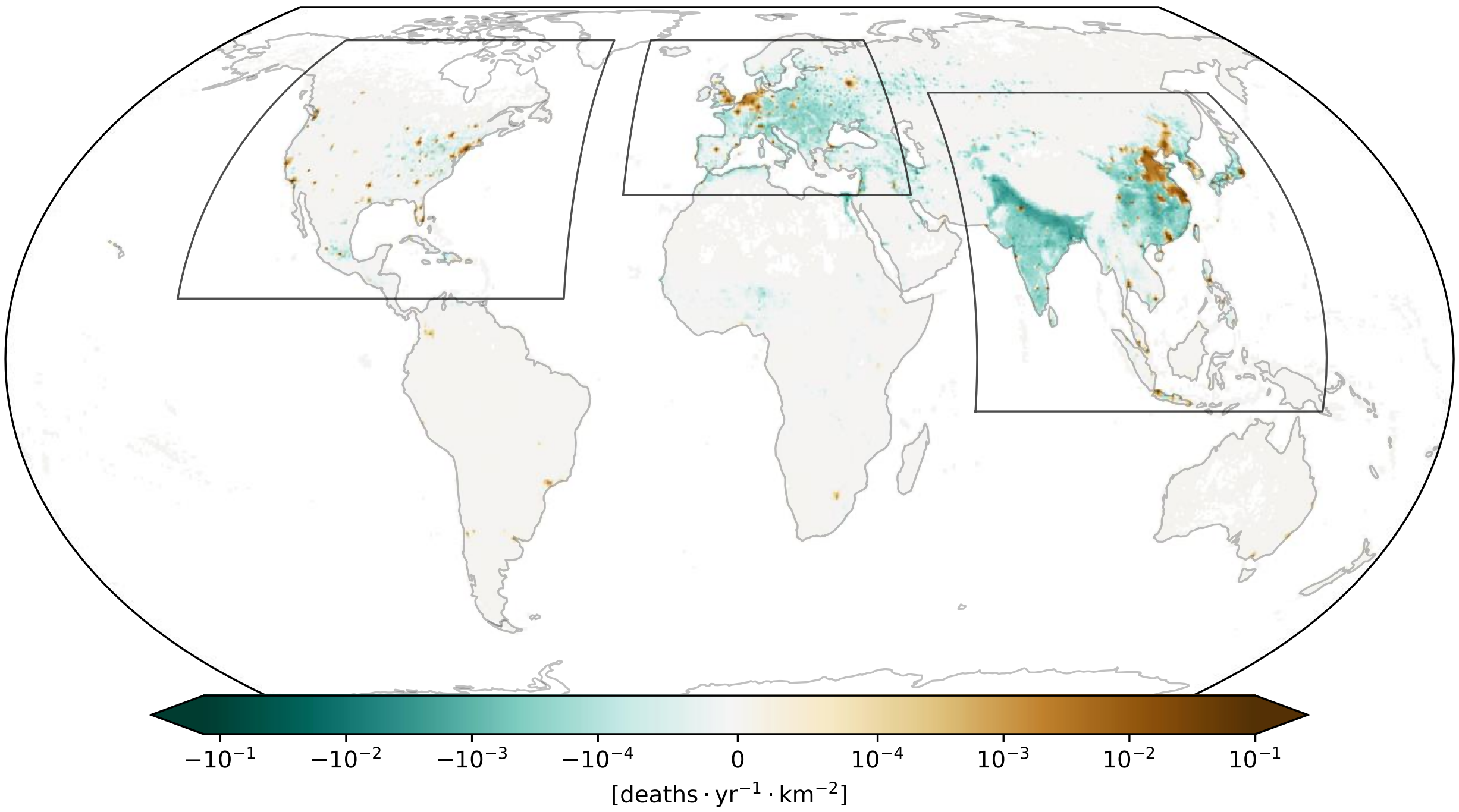

Supplementary Fig. 25: Modeled excess mortality from NO_2 attributed to aircraft full-flight emissions in 2019. Negative values indicate lower mortality in the simulation with aircraft emissions.

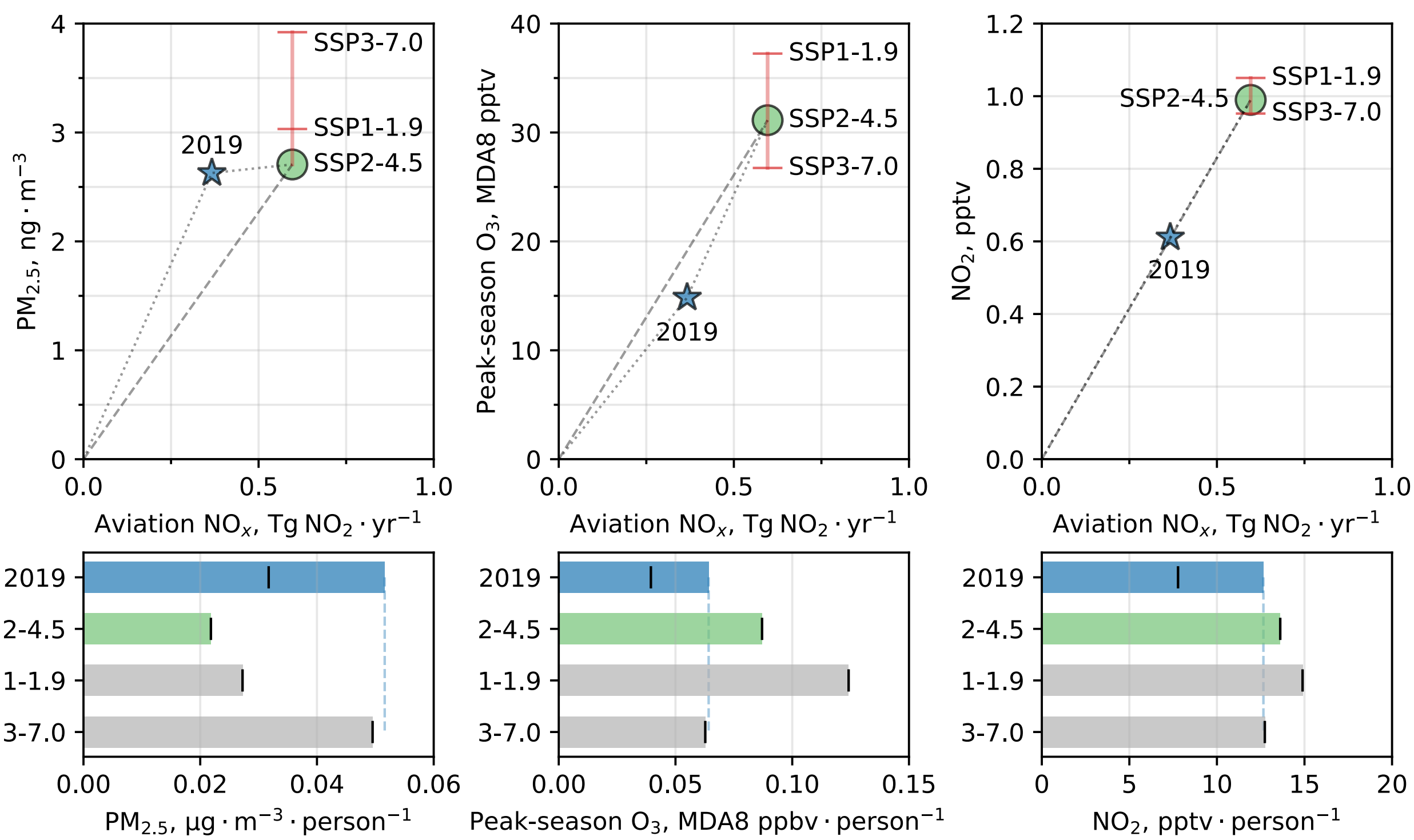

Supplementary Fig. 26: Sensitivity of global air quality to LTO emissions. Top: global annual mean LTO-attributable pollutant levels versus NO_x emitted. The gray lines connecting the points are shown to highlight changes in sensitivity. Bottom: global annual average population exposure attributable to LTO emissions for different scenarios. Filled horizontal bars indicate values normalized according to the amount of LTO NO_x to 2040 levels; unnormalized values are indicated by black vertical dashes. Note the different $PM_{2.5}$ and ozone units across top and bottom plots.

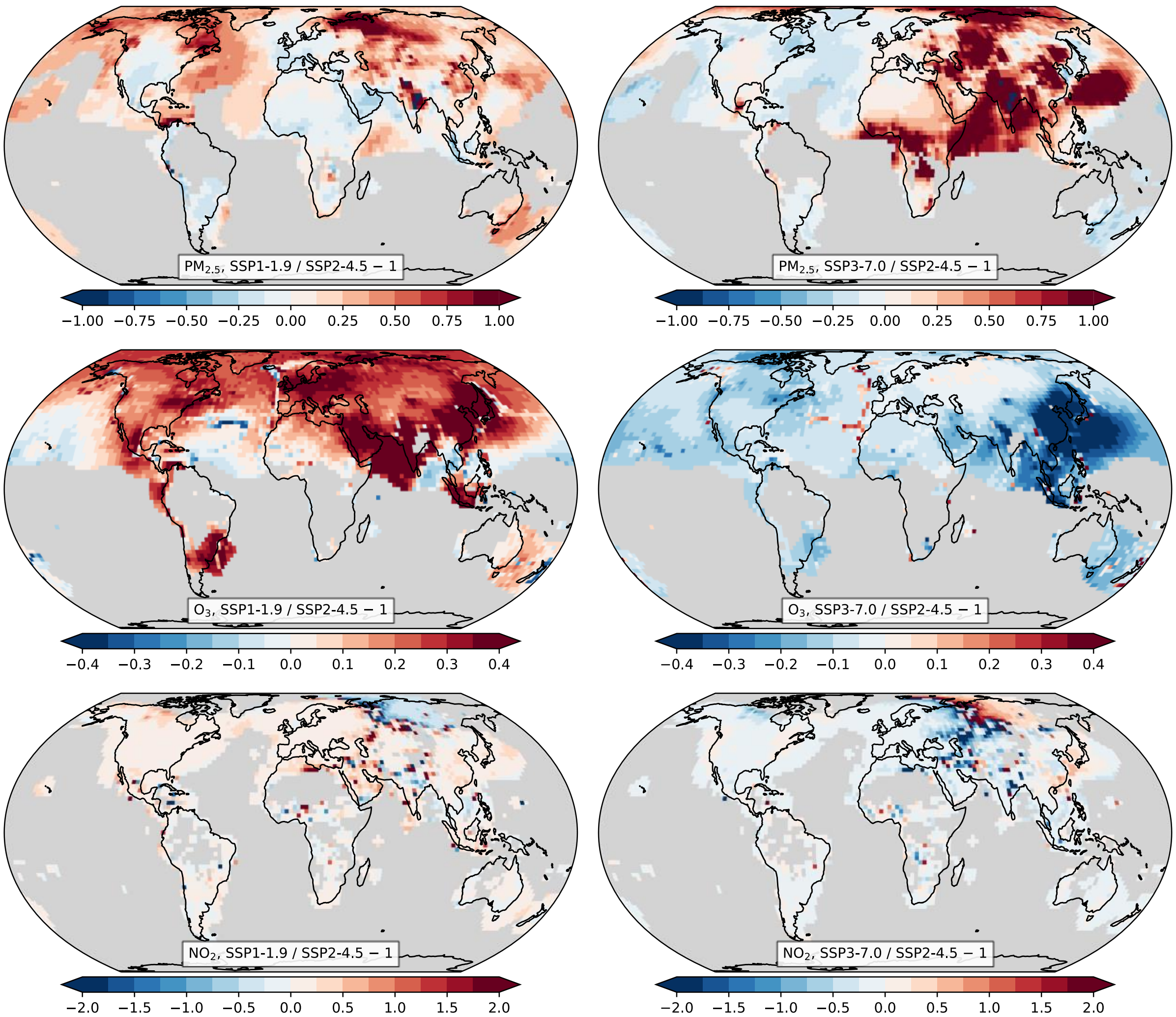

Supplementary Fig. 27: Sensitivity of modeled air quality impacts of LTO emissions to future non-aviation emissions scenarios. Relative difference in ground-level concentrations attributable to LTO emissions in 2040 between a less polluted scenario (SSP1-1.9) or a more polluted scenario (SSP3-7.0) and a more likely scenario (SSP2-4.5). Grid cells where values for SSP2-4.5 are below the 50th percentile are grayed out.

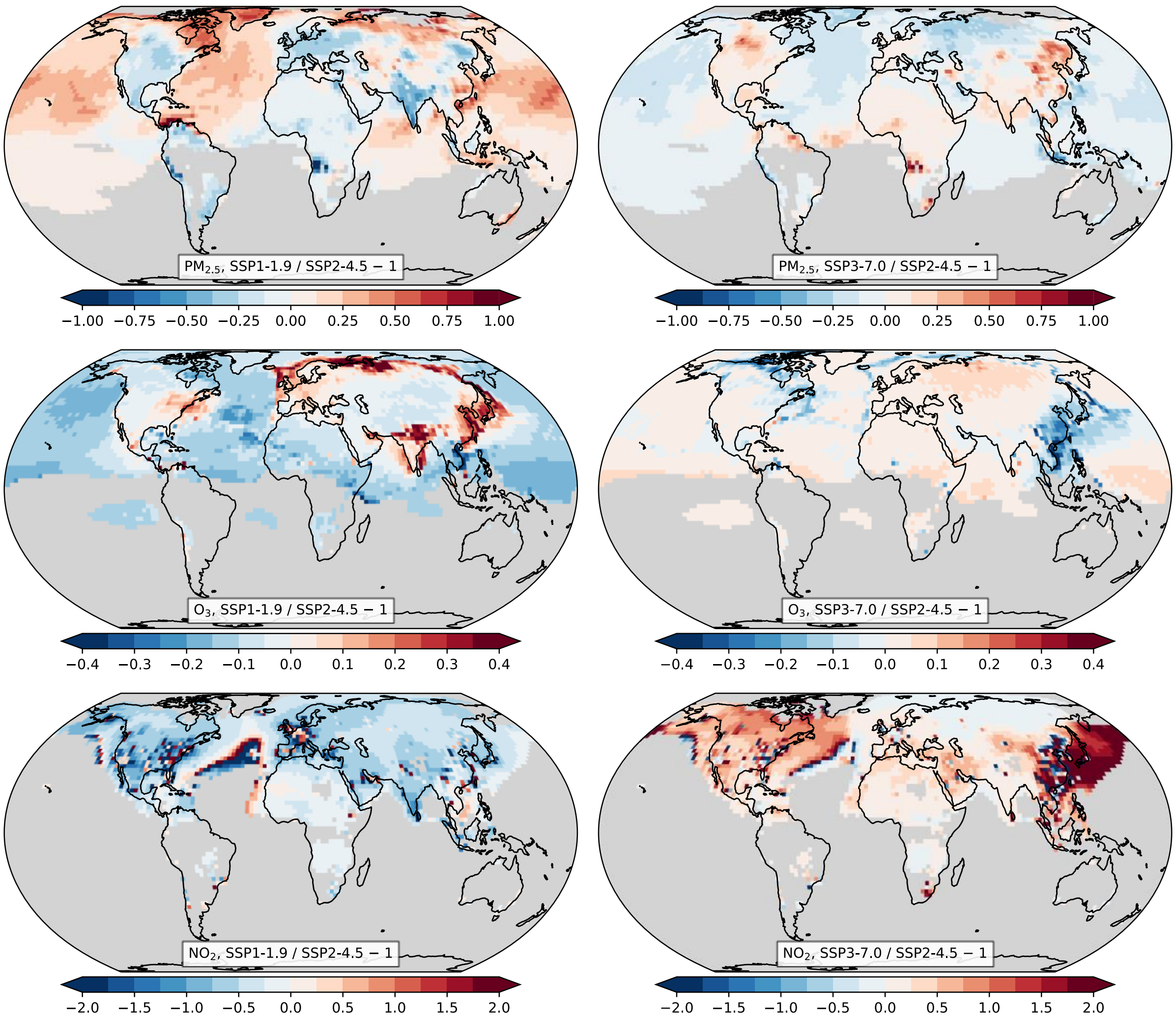

Supplementary Fig. 28: Sensitivity of modeled air quality impacts of full-flight emissions to future non-aviation emissions scenarios. Relative difference in ground-level concentrations attributable to full-flight aircraft emissions in 2040 between a less polluted scenario (SSP1-1.9) or a more polluted scenario (SSP3-7.0) and a more likely scenario (SSP2-4.5). Grid cells where values for SSP2-4.5 are below the 50th percentile are grayed out.

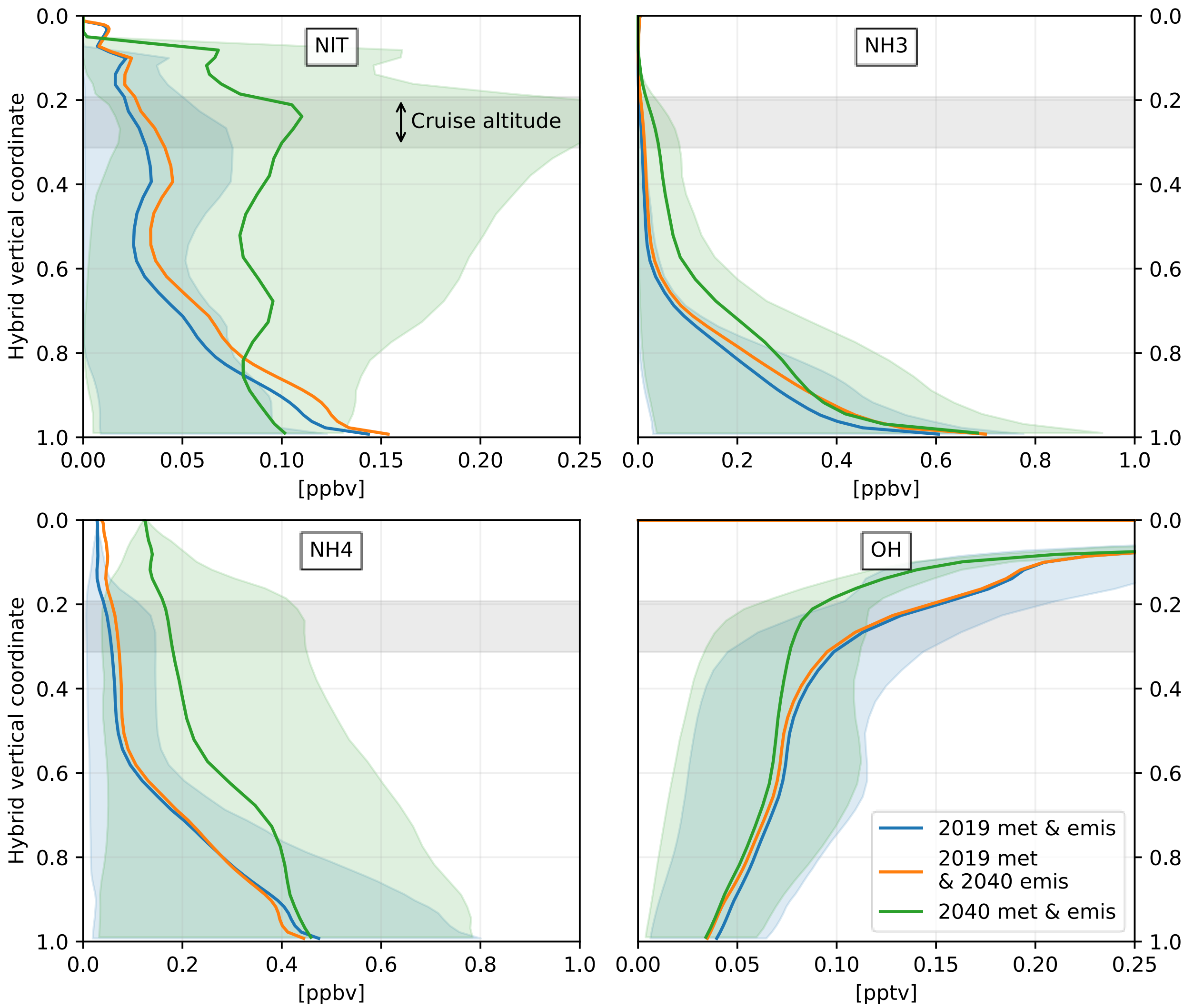

Supplementary Fig. 29: Modeled vertical concentration profiles with different emissions and meteorology fields. Global annual average mixing ratio (dry air basis) of model tracers for inorganic nitrates (NIT), NH_3, NH_4^+, and OH per hybrid pressure vertical coordinate, which is roughly equal to the fraction of atmospheric column above that point. Comparison of 2019 meteorology from MERRA-2 against 2040 emissions and meteorology from GCAP 2.0. Shaded areas indicate the 68% range of values (1 σ) at each vertical level for two of the three simulations. The band of "cruise altitude" indicates the altitude range where roughly 70% of full-flight aircraft NO_x emissions are released.

Supplementary References

1. International Civil Aviation Organization. *Post-COVID-19 Forecasts Scenarios*. https://www.icao.int/sustainability/Pages/Post-Covid-Forecasts-Scenarios.aspx (2021).
2. International Civil Aviation Organization. Presentation of 2019 Air Transport Statistical Results. (2020).
3. International Air Transport Association. Airline Industry Economic Performance - December 2019 - Report. (2019).
4. International Civil Aviation Organization. ICAO Long-Term Traffic Forecasts. (2018).
5. Graver, B. & Rutherford, D. *Low-Cost Carriers and U.S. Aviation Emissions Growth, 2005 to 2019*. (2021).
6. Dray, L. An analysis of the impact of aircraft lifecycles on aviation emissions mitigation policies. *Journal of Air Transport Management* **28**, 62–69 (2013).
7. International Civil Aviation Organization. *Final Report of the Independent Expert Operational Goals Group (IEOGG)*. (2014).
8. Quadros, F. D. A., Snellen, M., Sun, J. & Dedoussi, I. C. Global Civil Aviation Emissions Estimates for 2017–2020 Using ADS-B Data. *Journal of Aircraft* **59**, 1394–1405 (2022).
9. Fleming, G. G. & de Lépinay, I. Environmental Trends in Aviation to 2050. in *ICAO Environmental Report 2019: Aviation and Environment – Destination Green the Next Chapter* 17–23 (International Civil Aviation Organization, Montréal, 2019).
10. Cumpsty, N. *et al. Doc 10127, Independent Expert Integrated Technology Goals Assessment and Review for Engines and Aircraft*. (International Civil Aviation Organization, Montréal, 2019).
11. International Civil Aviation Organization. *ICAO Annex 16: Environmental Protection, Volume II -- Aircraft Engine Emissions*. (International Civil Aviation Organization, 2017).

12. World Ozone and Ultraviolet Radiation Data Center. Ozonesonde. https://doi.org/10.14287/10000008.

13. US Environmental Protection Agency. Air Quality Data Mart [internet database].

14. Environment and Climate Change Canada (ECCC). National Air Pollution Surveillance (NAPS) Program.

15. European Environment Agency. Air Quality Download Service - Primary Verified Data (E1a). https://eeadmz1-downloads-webapp.azurewebsites.net/.

16. Central Pollution Control Board. *National Ambient Air Quality Status & Trends 2019*. (2020).

17. Sharma, D. & Mauzerall, D. Analysis of Air Pollution Data in India between 2015 and 2019. *Aerosol Air Qual. Res.* **22**, 210204 (2022).

18. Silver, B., Reddington, C. L., Chen, Y. & Arnold, S. R. A decade of China's air quality monitoring data suggests health impacts are no longer declining. *Environment International* **197**, 109318 (2025).

19. Network Center for EANET. *Data Report 2019*. 332 https://monitoring.eanet.asia/document/public/index (2020).

20. Park, R. J. *et al.* Multi-model intercomparisons of air quality simulations for the KORUS-AQ campaign. *Elementa: Science of the Anthropocene* **9**, 00139 (2021).

21. Eastham, S. D., Weisenstein, D. K. & Barrett, S. R. H. Development and evaluation of the unified tropospheric–stratospheric chemistry extension (UCX) for the global chemistry-transport model GEOS-Chem. *Atmospheric Environment* **89**, 52–63 (2014).

22. Eastham, S. D. & Barrett, S. R. H. Aviation-attributable ozone as a driver for changes in mortality related to air quality and skin cancer. *Atmospheric Environment* **144**, 17–23 (2016).

23. Bates, K. H., Evans, M. J., Henderson, B. H. & Jacob, D. J. Impacts of updated reaction kinetics on the global GEOS-Chem simulation of atmospheric chemistry. *Geoscientific Model Development* **17**, 1511–1524 (2024).

24. Burnett, R. *et al.* Global estimates of mortality associated with long-term exposure to outdoor fine particulate matter. *Proc Natl Acad Sci USA* **115**, 9592–9597 (2018).

25. Chen, J. & Hoek, G. Long-term exposure to PM and all-cause and cause-specific mortality: A systematic review and meta-analysis. *Environment International* **143**, 105974 (2020).

26. Crouse, D. L. *et al.* Ambient $PM_{2.5}$, O_3, and NO_2 Exposures and Associations with Mortality over 16 Years of Follow-Up in the Canadian Census Health and Environment Cohort (CanCHEC). *Environ Health Perspect* **123**, 1180–1186 (2015).

27. Hoek, G. *et al.* Long-term air pollution exposure and cardio- respiratory mortality: a review. *Environ Health* **12**, 43 (2013).

28. Jerrett, M. *et al.* Comparing the Health Effects of Ambient Particulate Matter Estimated Using Ground-Based versus Remote Sensing Exposure Estimates. *Environ Health Perspect* **125**, 552–559 (2017).

29. Pope, C. A. *et al.* Mortality Risk and Fine Particulate Air Pollution in a Large, Representative Cohort of U.S. Adults. *Environ Health Perspect* **127**, 077007 (2019).

30. Strak, M. *et al.* Long term exposure to low level air pollution and mortality in eight European cohorts within the ELAPSE project: pooled analysis. *BMJ* **374**, n1904 (2021).

31. Turner, M. C. *et al.* Long-Term Ozone Exposure and Mortality in a Large Prospective Study. *Am J Respir Crit Care Med* **193**, 1134–1142 (2016).

32. Vodonos, A., Awad, Y. A. & Schwartz, J. The concentration-response between long-term $PM_{2.5}$ exposure and mortality; A meta-regression approach. *Environmental Research* **166**, 677–689 (2018).

33. Wu, X., Braun, D., Schwartz, J., Kioumourtzoglou, M. A. & Dominici, F. Evaluating the impact of long-term exposure to fine particulate matter on mortality among the elderly. *Sci. Adv.* **6**, eaba5692 (2020).

34. Brauer, M. *et al.* Global burden and strength of evidence for 88 risk factors in 204 countries and 811 subnational locations, 1990–2021: a systematic analysis for the Global Burden of Disease Study 2021. *The Lancet* **403**, 2162–2203 (2024).

35. Cakmak, S. *et al.* Associations between long-term $PM_{2.5}$ and ozone exposure and mortality in the Canadian Census Health and Environment Cohort (CANCHEC), by spatial synoptic classification zone. *Environment International* **111**, 200–211 (2018).

36. Di, Q. *et al.* Air Pollution and Mortality in the Medicare Population. *N Engl J Med* **376**, 2513–2522 (2017).

37. Huangfu, P. & Atkinson, R. Long-term exposure to NO_2 and O_3 and all-cause and respiratory mortality: A systematic review and meta-analysis. *Environment International* **144**, 105998 (2020).

38. Kasdagli, M.-I., Orellano, P., Pérez Velasco, R. & Samoli, E. Long-Term Exposure to Nitrogen Dioxide and Ozone and Mortality: Update of the WHO Air Quality Guidelines Systematic Review and Meta-Analysis. *Int. J. Public Health* **69**, 1607676 (2024).

39. Jerrett, M. *et al.* Long-Term Ozone Exposure and Mortality. *N Engl J Med* **360**, 1085–1095 (2009).

40. Murray, C. J. L. *et al.* Global burden of 87 risk factors in 204 countries and territories, 1990–2019: a systematic analysis for the Global Burden of Disease Study 2019. *The Lancet* **396**, 1223–1249 (2020).

41. World Health Organization. *Global Health Estimates 2019: Deaths by Cuase, Age, Sex, by Country and by Region, 2000-2019*. (World Health Organization, Geneva, 2020).

42. Hughes, B. B. *et al.* Projections of global health outcomes from 2005 to 2060 using the International Futures integrated forecasting model. *Bull. World Health Organ.* **89**, 478–486 (2011).

43. Vollset, S. E. *et al.* Burden of disease scenarios for 204 countries and territories, 2022–2050: a forecasting analysis for the Global Burden of Disease Study 2021. *The Lancet* **403**, 2204–2256 (2024).

44. Institute for Health Metrics and Evaluation (IHME). GBD Foresight Visualization. https://vizhub.healthdata.org/gbd-foresight (2024).
